# Revisiting the Estimation of Dinosaur Growth Rates

**DOI:** 10.1371/journal.pone.0081917

**Published:** 2013-12-16

**Authors:** Nathan P. Myhrvold

**Affiliations:** Intellectual Ventures, Bellevue, Washington, United States of America; Monash University, Australia

## Abstract

Previous growth-rate studies covering 14 dinosaur taxa, as represented by 31 data sets, are critically examined and reanalyzed by using improved statistical techniques. The examination reveals that some previously reported results cannot be replicated by using the methods originally reported; results from new methods are in many cases different, in both the quantitative rates and the qualitative nature of the growth, from results in the prior literature. Asymptotic growth curves, which have been hypothesized to be ubiquitous, are shown to provide best fits for only four of the 14 taxa. Possible reasons for non-asymptotic growth patterns are discussed; they include systematic errors in the age-estimation process and, more likely, a bias toward younger ages among the specimens analyzed. Analysis of the data sets finds that only three taxa include specimens that could be considered skeletally mature (*i.e.*, having attained 90% of maximum body size predicted by asymptotic curve fits), and eleven taxa are quite immature, with the largest specimen having attained less than 62% of predicted asymptotic size. The three taxa that include skeletally mature specimens are included in the four taxa that are best fit by asymptotic curves. The totality of results presented here suggests that previous estimates of both maximum dinosaur growth rates and maximum dinosaur sizes have little statistical support. Suggestions for future research are presented.

## Introduction

Knowledge of the life histories of extinct species has increased enormously in recent decades, advanced by bone histology [1]–[16] and the measurement of ontogenetic growth rates for many species [17]–[19]. Histological estimation of age depends primarily on analysis of features known as lines of arrested growth (LAGs) [20], which are seen in thin sections of fossilized bone [6]. Strong evidence from extant amphibians, reptiles, birds and mammals suggests that, in many dinosaur taxa, LAGs were deposited annually while the animal was alive. Even in bones in which LAGs are not visible, “polish lines” sometimes appear, and Sander has argued [21] that these also represent markers for annual growth. Although the inference that each LAG represents one year of growth is still a subject of debate [22] and the number and distribution of LAGs varies in some specimens from one bone to another or is obscured by inter-element remodeling [10], [11], [13]–[15], [21]–[23], the analysis presented here adopts the common assumption in paleobiology that LAGs and polish lines are indeed annual markers.

Many of the other assumptions on which studies of dinosaur growth have routinely depended are more questionable, however, as are some of the statistical methodologies used. Insufficient attention has been given to problematic issues in estimating the ages and masses that dinosaurs achieved during their lives, in fitting growth curves to the data sets available, and in interpreting the results of curve fits.

Two distinct analytical approaches, the whole-bone method and the longitudinal method, have been used to gather age/size data sets, from which biological growth parameters can be calculated. Each approach involves similar steps – age estimation, mass estimation and growth curve fitting – but they differ in their details between approaches, and between studies. Both approaches require careful handling of the uncertainties involved in the estimation of ages and masses, and of the assumptions and statistical methods used to fit growth curves to observed and derived data.

### Whole-bone Method

Chinsamy-Turan [6] appears to have been the first to use the “whole-bone” method to fit growth curves for dinosaurs by using the linear dimension of a whole bone as the size metric and a count of LAGs in a bone to estimate age at time of death. Erickson and Tumanova [24] extended the method to estimate the mass of an animal from the length of a long bone, and the approach was extended further in later studies [16]–[18], [24]–[29]. The method has been applied to 11 dinosaur taxa by Erickson and coworkers and to three additional taxa by Bybee *et al.*, Lehman, and Lee and Werning (Table 1). Werning [30] and Lee and Werning [31] applied a variation of the whole-bone method to the ornithopod *Tenontosaurus* (without the developmental mass extrapolation (DME) step, discussed below), although bone dimensions have not been published. Bybee *et al.* [32] presented data that can be used for the method.

**Table 1:** Dinosaur growth rate studies.

Taxon	Steps	Reference	Priorstudy
*Albertosaurus sarcophagus*	W/1a,1b, 2, 3	[25]	
*Albertosaurus sarcophagus*	W/1a,1b, 2, 3	[17]	[25]
*Apatosaurus excelsus* *	W/1a, 1b, 2, 3	[20]	[98]
*Daspletosaurus torosus* *	W/1a,1b, 2, 3	[25]	
*Gorgosaurus libratus*	W/1a,1b, 2, 3	[25]	
*Maiasaura peeblesorum* *	W/2,3	[20]	[11]
*Massospondylus carinatus*	W/3	[20]	[6]
*Psittacosaurus lujiatunensis*	W/1a, 1b, 1c, 2, 3	[18]	
*Psittacosaurus mongoliensis*	W/1a, 1b, 2, 3	[24]	
*Psittacosaurus mongoliensis*	W/1a, 1b, 2, 3	[32]	[24]
*Shuvuuia deserti* *	W/2, 3	[20]	[117]
*Syntarsus rhodesiensis*	W/3	[20]	[5]
*Tyrannosaurus rex*	W/1a,1b, 2, 3	[25]	
*Tyrannosaurus rex*	W/1a,1b, 2, 3	[17]	[25]
*Allosaurus fragilis*	W/1a, 2b, 3a, 3b, 4c	[20]	
*Saurornitholestes*	W/2, 3	[33]	[3]
*Tenontosaurus* *	W/2,3	[31]	[30]
*Apatosaurus excelsus*	L/1b, 2a, 3a,3b, 4c	[35]	[98]
*Alamosaurus sanjuanensis*	L/1b, 3a,3b, 4c	[35]	
*Allosaurus fragilis*	L/1a, 2b, 3b, 4c	[32]	
*Allosaurus fragilis*	L/1a, 2b, 3b, 4c	[31]	[32]
*Hypacrosaurus stebingeri*	L/1a, 4a	[38]	
Northampton sauropod	L/1a, 3a,3b, 4c	[37]	[3]
*Citipati osmolskae* *	L/1?, 2, 3b, 4c	[25]	
*Deinonychus antirrhopus* *	L/1?, 2, 3b, 4c	[25]	
*Oviraptor philoceratops* *	L/1?, 2, 3b, 4c	[25]	
*Pachyrhinosaurus sp.* *	L/1?, 2, 3b, 4c	[29]	
*Troodon formosus* *	L/1?, 2, 3b, 4c	[25]	[7]
*Troodontidae nov. sp.* *	L/1?, 2, 3b, 4c	[25]	
*Mamenchisaurus sp.* *	not reported	[37]	

Steps indicate the method performed in the referenced studies and refer to the steps in the whole-bone (W) and longitudinal (L) methods described in the text. In some cases, age estimation (step 1) or mass estimation (step 2) was performed in a prior study, as indicated in the rightmost column.

Cases in which the published data or details about the analytic method used are insufficient to replicate the work.

Although details of the whole-bone method vary slightly in the literature, it generally includes two or more of the following steps:

1. **Estimate ages.** LAGs are counted by microscopic examination of thin sections of bone, typically cut from the femur but sometimes from other bones. Age at the time of death is then estimated by adjusting or supplementing the raw LAG count:
  - missing LAGs are estimated or “retrocalculated” by using one of several techniques [24], [25], [27], [32];
  - once ages have been estimated for several specimens, linear regression is used to fit a line relating bone dimension to age [18]. The regression results are then used to estimate the ages of any specimens for which LAGs could not be counted directly.
2. **Estimate masses.** Two previous studies [31], [33] used the formula of Anderson *et al.* [34] that relates the body mass to the circumference of the limb bones with a scaling exponent of 2.73 (see Discussion). Other studies that estimate masses, however, have typically reported using DME [24], which typically involves two steps:
  - the length 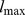 and midpoint circumference of the longest femur available for the study are recorded, and the technique of Anderson *et al.* [34] or some other method is then used to estimate the mass 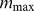 of the specimen;
  - “extrapolation” (*sic*; it is actually interpolation) of an estimated mass for each of the specimens is done by using the length 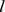 of the specimen femur in the equation, with a scaling exponent of 3 (see Discussion).
3. **Fit a growth curve.** An asymptotic growth curve–frequently a logistic, Gompertz or von Bertalanffy curve–is fit to the age–mass data from multiple specimens and the parameters of the fit curve are used to derive the growth rate and other parameters of biological interest (such as the maximum asymptotic size for the taxon).

### Longitudinal Method

The longitudinal method for growth determination was first published by Woodward [35] in studies of *Apatosaurus excelsus* and *Alamosaurus sanjuanensis*. The method was subsequently applied by Bybee *et al.* [32] to study *Allosaurus fragilis*; by Lee [36] in papers on several taxa; by Lehman and Woodward [37] in work on the sauropod *Janenschia robustus* and an unidentified sauropod from Northampton, UK; and by Cooper *et al.* [38] in studies of *Hypacrosaurus stebingeri.* Lehman [33] applied a variation of the method in a paper on *Saurornitholestes.* Table 1 summarizes the use of the longitudinal method in the studies referenced above.

**Figure 1:**
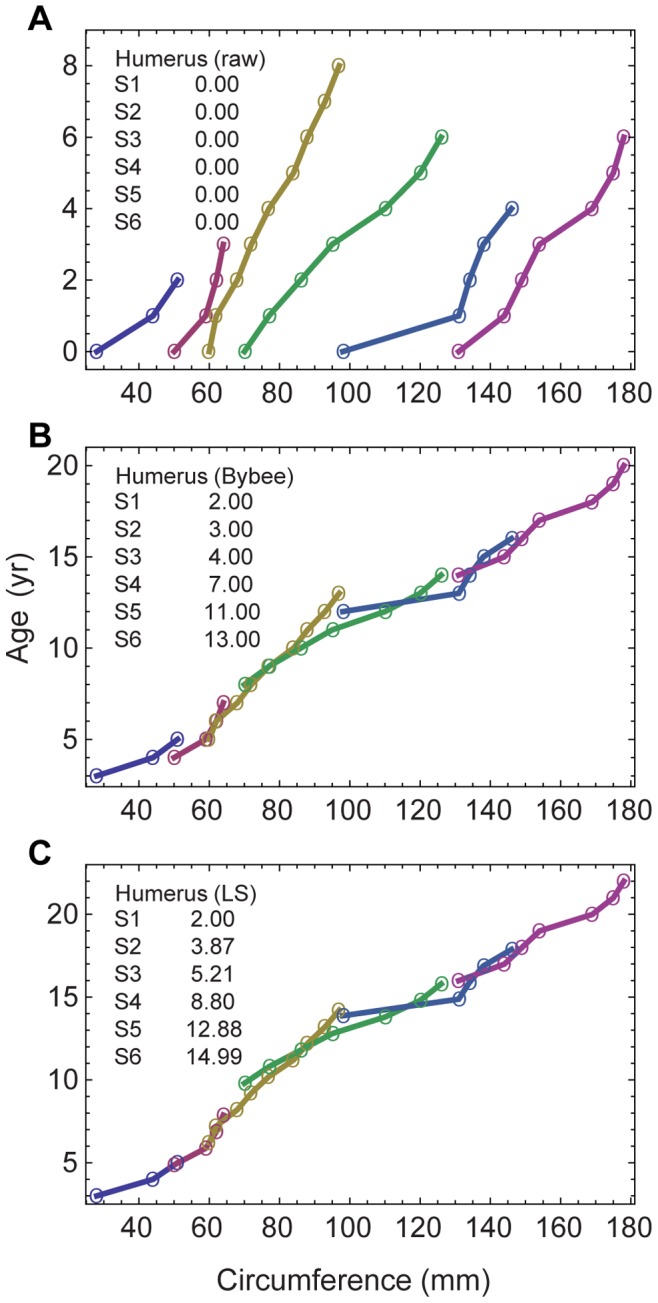
Longitudinal time series for *Allosaurus fragilis* humeri. **A**, the raw time series from Bybee *et al.* [32]. Each series starts at a time offset of 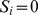. **B**, the result of applying offsets 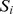 as assigned in [32] based on matching the curves by eye. **C**, the result of calculating 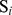 by a least-squares matching algorithm that uses non-integer offsets.

The longitudinal method was also used by Lee [36] and by Wings *et al.* [39] in a study of the sauropod *Mamenchisaurus*; although data and growth parameters were not included in that report, they are in preparation (Wings personal communication 2013). Two relatively recent studies by Erickson *et al.* [26], [29] appear to use the longitudinal method, but the papers do not specify the steps used in the analyses and do not include sufficient data to enable reconstruction or replication of the method used. Fig. 1 illustrates the application of the longitudinal process to the *Allosaurus* humerus data from multiple specimens spanning a wide range of sizes by Bybee *et al.* [32]. In the whole-bone method, the age of each specimen is determined by retrocalculation rather than alignment, and each specimen would be represented only by its oldest (right most) data point.

The longitudinal method typically involves four steps:

1. **Measuring LAGs.** LAGs are not merely counted, but also measured:
  - a LAG is traced on digital micrograph, and its circumference is measured;
  - alternatively, the radial distance from one LAG to the next, or from the center of the bone to the LAG, is measured.
  Multiple measurements can be compiled for a single specimen to yield a time series of ages and LAG sizes–a growth history–for that specimen, much like the data sets available in longitudinal studies in medicine or social science.
2. **Correlating multiple time series.** In cases where multiple LAG time series have been obtained from the corresponding bone in different specimens, a composite time series can be constructed to span a wider range of sizes and ages. To make the composite, time series are matched by adding a time offset:
  - digital micrographs are overlaid and scaled to matching sizes;
  - alternatively, graphs of the time series are aligned, generally as judged by sight, by adjusting the offset.
3. **Estimating masses or scaling LAG sizes.** Raw LAG series measurements are usually rescaled or processed before further analysis:
  - mass is estimated by using DME or another scaling relationship of bone dimension to body mass;
  - alternatively, the LAG size measurements are rescaled linearly relative to either the outermost LAG (if the data set comprises a single specimen), to the largest dimension known for the specimens within the study, or to the largest dimension known for the taxon as a whole.
4. **Fitting a growth curve.** A growth curve is fit to the rescaled data and biological parameters are then derived from the fit curve and its parameters. The fit is made either:
  - directly to the time series of LAG dimensions;
  - to the body mass estimated in step (3a) above; or
  - to the fraction of largest dimension as obtained in step (3b) above.

### Objectives

I conducted a systematic reanalysis of prior studies of the growth of 14 dinosaur taxa, based on both the whole-bone and longitudinal method, with a particular focus on identifying and correcting underlying assumptions that may be unjustified or statistical methodologies that increase expected errors. I attempted to replicate the derivation of previously published growth curves and, in cases where replication failed, possible reasons for the disparity were examined. An improved statistical approach was used to obtain new estimates of skeletal growth rates for the 14 taxa and to determine whether statistical support was strongest for asymptotic growth or some other growth pattern. The resulting growth rates were compared to prior analysis. Monte Carlo and bootstrap error analysis was performed to estimate sensitivity of these results to population sampling and errors in age estimation or growth variation.

## Methods

An extensive review of the literature on dinosaur growth identified 31 data sets of sufficient detail for reanalysis, covering 14 taxa of dinosaurs (Table 2). Each data set was recorded from a single prior study except for *Tyrannosaurus* 2, which combines specimen data from three reports [25], [40], [41]. Note that *Syntarsus rhodesiensis* was renamed *Megapnosaurus rhodesiensis* subsequent to the referenced prior growth studies [42]. Here I will refer to the taxon as *Syntarsus* for consistency with prior work.

**Table 2:** Dinosaur growth data sets used for model fitting.

Data set name	Taxon	Bone	Dimension	N	M	Type	Reference
*Albertosaurus*	*Albertosaurus sarcophagus*	femur	length	5	5	W	[25]
*Daspletosaurus*	*Daspletosaurus torosus*	femur	length	3	3	W	[25]
*Gorgosaurus*	*Gorgosaurus libratus*	femur	length	5	5	W	[25]
*Massospondylus*	*Massospondylus carinatus*	femur	length	14	14	W	[20]
*Psittacosaurus l*1	*Psittacosaurus lujiatunensis*	femur	length	39	39	W	[18]
*Psittacosaurus l*2	*Psittacosaurus lujiatunensis*	femur	length	80	80	W	[18]
*Psittacosaurus l*3	*Psittacosaurus lujiatunensis*	femur	length	80	80	W	[18]
*Psittacosaurus l*4	*Psittacosaurus lujiatunensis*	femur	length	80	80	W	[18]
*Psittacosaurus m*1	*Psittacosaurus mongoliensis*	femur	length	7	7	W	[24]
*Syntarsus*	*Syntarsus rhodesiensis*	femur	length	10	10	W	[5], [118]
*Tyrannosaurus* 1	*Tyrannosaurus rex*	femur	length	7	7	W	[25]
*Tyrannosaurus* 2	*Tyrannosaurus rex*	femur	length	9	9	W	[25], [40], [41]
*Saurornitholestes*	*Saurornitholestes*	femur	length	9	9	W	[33]
*Allosaurus* hl	*Allosaurus fragilis*	humerus	length	6	6	W	[32]
*Allosaurus* ul	*Allosaurus fragilis*	ulna	length	5	5	W	[32]
*Allosaurus* fl	*Allosaurus fragilis*	femur	length	6	6	W	[32]
*Apatosaurus*	*Apatosaurus excelsus*	pubis	radial	13	2	L	[35]
*Alamosaurus*	*Alamosaurus sanjuanensis*	humerus	radial	9	1	L	[35]
Northampton	Northampton sauropod	pubis	radial	21	1	L	[37]
*Janenschia*	*Janenschia robustus*	femur	radial	16	1	L	[37]
*Allosaurus* fc1	*Allosaurus fragilis*	femur	circumference	38	6	L	[32]
*Allosaurus* fc2	*Allosaurus fragilis*	femur	circumference	38	6	L	[32]
*Allosaurus* fc3	*Allosaurus fragilis*	femur	circumference	19	3	L	[32]
*Allosaurus* fc4	*Allosaurus fragilis*	femur	circumference	19	3	L	[32]
*Allosaurus* hc1	*Allosaurus fragilis*	humerus	circumference	35	5	L	[32]
*Allosaurus* hc2	*Allosaurus fragilis*	humerus	circumference	35	5	L	[32]
*Allosaurus* tc1	*Allosaurus fragilis*	tibia	circumference	20	3	L	[32]
*Allosaurus* tc2	*Allosaurus fragilis*	tibia	circumference	20	3	L	[32]
*Allosaurus* uc1	*Allosaurus fragilis*	ulna	circumference	30	5	L	[32]
*Allosaurus* uc2	*Allosaurus fragilis*	ulna	circumference	30	5	L	[32]
*Hypacrosaurus* fc	*Hypacrosaurus stebingeri*	femur	circumference	7	1	L	[38]
*Hypacrosaurus* tc	*Hypacrosaurus stebingeri*	tibia	circumference	8	1	L	[38]

N is the number of data points in the data set; M is the number of specimens from which the data were obtained. Type indicates the kind of data set: whole bone (W) or longitudinal (L). See Table S6 for the data included in each set.

In data sets comprising longitudinal measurements on multiple specimens, least-squares optimization was used to minimize the difference between every pair of series in the regions of overlap (Fig. 1C). This computational matching process, which is more objective than aligning the offsets by sight, does not fully specify the age unless the smallest specimen is known to be a neonate. In all other cases, retrocalculation is required to set the youngest age. The offsets derived by the least-squares method are, in general, non-integer values (*i.e.*, this method does not assume that each specimen had the same birthday).

**Figure 2:**
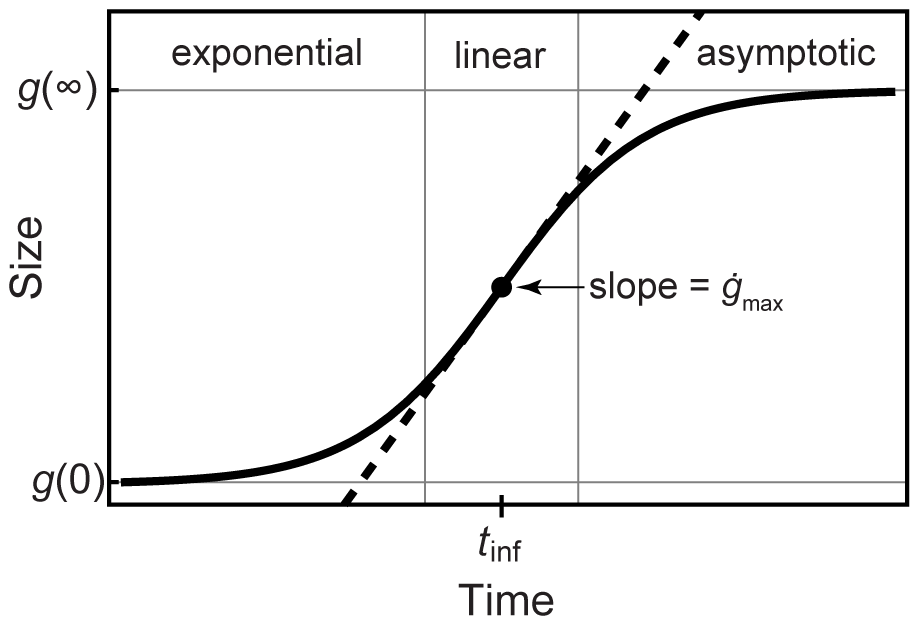
Three phases of growth in a typical sigmoidal curve. A sigmoidal curve (solid black line) typically includes an initial exponential phase, an approximately linear phase (which contains the inflection point at which the growth rate is maximal) and finally an asymptotic phase, in which the curve approaches a constant asymptote 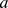 as 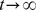. In some cases, the initial exponential phase is so short as to be imperceptible, but the linear and asymptotic regions are features of all sigmoidal curves. Attenuating curves are similar but lack the initial exponential phase, much like a sigmoidal curve that starts with its inflection point at 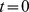.

Seventy seven growth functions (Table S1 and Table S2) were fit to the 31 data sets by using nonlinear least-squares regression functions in commercial mathematical software (Mathematica 9.01, Wolfram Research). Fifteen of the 77 functions used are increasing functions; the remaining 62 are asymptotic (48 sigmoidal and 14 attenuating). Sigmoidal curves are illustrated in schematic form in Fig. 2. Sigmoidal growth includes three phases: an initial exponential phase, a phase in which growth is approximately linear with time, and finally, an asymptotic phase. Two, three and four-parameter models were included in the analysis. Bone dimension was used as the independent variable in performing the regressions. Text S7 presents additional details about the fitting methods used. Biological parameters of interest, such as growth rate and maximum asymptotic size, were then derived from the parameters of the best fit growth curves.

Age–mass data sets were also collected from prior studies (Table S7). In several cases, digital scans of published figures were used with image-processing functions to verify the data sets and graphs. These data sets were used to attempt to replicate the original analyses (Table 3). As in the original studies, time was used as the independent variable for the replication regressions.

**Table 3:** Attempted replication of results from references [18], [20], [24], [25] and [37].

		Maximum asymptotic size, *a* (kg)			Peak growth rate, 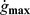 (kg/yr)		
Taxon	Ref.	Reported	Best fit	Ratio	Reported	Best fit	Ratio
*Tyrannosaurus rex* A	25	5556	5859	0.948	791	467	1.695*
*Gorgosaurus libratus* A	25	1239	1748561	0.001*	117	39286	0.003*
*Albertosaurus sarcophagus* A	25	1223	1229	0.995	131	126	1.040
*Massospondylus carinatus* A	20	281	4521733	0.000*	35.0	223477	0.000*
*Syntarsus rhodesiensis* A	20	18.8	19.0	0.991	10.5	5.60	1.876*
*Psittacosaurus mongoliensis* A	20	22.7	24.0	0.948	5.50	5.77	0.953
*Psittacosaurus mongoliensis* C	24	25.2	26.8	0.942	4.66	5.30	0.879*
*Psittacosaurus lujiatunensis* 1A	18	37.4	50.9	0.734*	5.14	5.25	0.979
*Apatosaurus*	37	25952	28618	0.907	519	538	0.964
*Alamosaurus*	37	32663	29326	1.114*	1089	1017	1.071
*Janenschia*	37	14029	16428	0.854*	624	658	0.948
Northampton	37	9000	9130	0.986	260	242	1.074

“Reported” parameter values were derived from regression equations published in the cited references. In some cases this differs from parameters (particularly maximum growth rate) quoted in the same papers–see Text S1. “Best fit” parameter values are the results from attempted replication by using the same data set, growth function and methodology as the cited reference. Note that analysis of these data sets with the methods described in this paper gives different results in many cases (see Table 4, Table 5 and Table S8). Two important biological parameters–maximum asymptotic size and peak growth rate–are shown here; see Text S1 and Table S4 for further details.

^*^Cases in which the replicated (Best fit) results differ from reported results by 10% or more. Four of the 11 taxa have published results for both asymptotic size and growth rate that can be replicated within that tolerance.

For each regression performed, the quality of fit was evaluated by calculating the relative corrected Akaike information criterion, 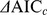 [43], and 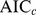 using the same software responsible for the curve fitting. For each data set, those regressions yielding 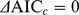 were selected as best fits, and those having 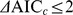 were interpreted as having strong statistical support. Because 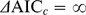 when the number of data points available is insufficient to support the number of parameters used in the model, this method prevents the selection of models that overfit the data.

To quantify the impact of the choice of independent variable, a Monte Carlo simulation was performed. The simulation drew 500 sets of data from the logistic equation (Text S2 and Fig. S8). Each synthetic data set sampled 20 points from a logistic curve having homoscedastic errors in age estimation, consistent with published estimates for dinosaur growth (Text S2). A homoscedastic error model assumes age estimation errors of equal magnitude, independent of age. Another 500 data sets were created from a logistic curve having heteroscedastic errors, where the error is assumed to be a percentage of the age. The maximum asymptotic value was estimated for each data set by using two alternative regression approaches: age as the independent variable, and age as the dependent variable.

To quantify the impact of the choice of model, the 62 asymptotic curves in this study were fit to synthetic data drawn from linear and cubic curves. Monte Carlo simulation was also used to quantify the impacts of the choice of different asymptotic models on data drawn from another asymptotic model. Asymptotic curves from Table S1 were fit to each of the 500 synthetic logistic data sets and used to generate mean estimates of maximum asymptotic size.

Bootstrap resampling [44] was used to estimate the sensitivity of dinosaur growth rate studies to population sampling (Text S5). Five hundred distinct samples were drawn at random from the data set. A subset of models (both asymptotic and increasing) that were a best fit to at least one dinosaur taxon were fit to each of the 500 random samples, and fits were then evaluated by calculating 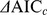.

To assess the effects of errors in age estimation, I performed Monte Carlo simulations, each having 500 trials, on the *Tyrannosaurus* 2 data set. Three simulations used 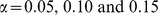, corresponding to normally distributed heteroscedastic age estimation errors of 5%, 10% and 15% of the age. In another series of Monte Carlo experiments, age estimation errors were modeled by homoscedastic errors which are normally distributed with a standard deviation of 0.25, 0.5 and 1 year.

To assess specimen maturity, I determined the extent of the region in which an asymptotic model is supported by data by rescaling the bone dimension 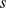 for each data point to
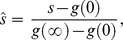
where 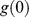 is the minimum value of the growth function and 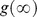 is the asymptote. The rescaling yields sizes 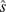 that fall between 0% and 100%. Within any set of growth data, the largest rescaled size, 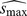, indicates the degree of maturity attained by the oldest specimen in the data set, as a percentage of the maximum asymptotic size estimated from the model fit.

The bone dimension 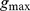 at which the maximum growth rate 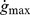 occurs was similarly rescaled to a percentage:
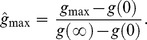



In order to avoid unsupported extrapolation, the growth rates presented in Table 4 were evaluated only at data points. Although it is possible to calculate the maximum growth rate at the inflection point in cases where sigmoidal models garner strong support, in most cases the calculation is unwarranted because the estimated inflection point is too far from data points to be sufficiently constrained by the data.

**Table 4:** Bone growth and growth rates for 31 data sets.

Data set	Model	Kind	*t*_m_ (yr)	*y*_m_ (cm)	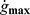 (cm/yr)	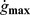 /*y*_m_ (%/yr)
*Tyrannosaurus* 1	Extreme Value 2	A	2	25.2	8.40	33.3%
*Tyrannosaurus* 2	Extreme Value 2	A	2	25.2	9.06	36.0%
*Gorgosaurus*	Linear 2	I	18	91.6	3.59	3.9%
*Albertosaurus*	Linear 2	I	24	89.5	3.16	3.5%
*Saurornitholestes*	Rational 2z	A	2	30	12.8	42.8%
*Saurornitholestes*	Michaelis Menten 2	A	2	30	12.8	42.8%
*Syntarsus*	Power 2	I	2	12.2	2.93	24.0%
*Allosaurus* fc1	Cubic 2	I	14	33.8	5.76	17.0%
*Allosaurus* fc2	Exponential 3	I	17.5	33.8	6.06	17.9%
*Allosaurus* fc3	Cubic 2b	I	14	19	0.94	4.9%
*Allosaurus* fc4	Quadratic 2	I	11.8	33.8	4.38	13.0%
*Allosaurus* hc1	Persistence 3a	I	20	17.8	1.39	7.8%
*Allosaurus* hc2	Persistence 3a	I	22.0	17.8	1.37	7.7%
*Allosaurus* uc1	Persistence 3a	I	17	12.5	0.92	7.3%
*Allosaurus* uc2	Persistence 3a	I	16.7	12.5	0.96	7.7%
*Allosaurus* hl	Cubic 2b	I	19	38.7	2.16	5.6%
*Allosaurus* ul	Exponential 2	I	16	24.5	1.44	5.9%
*Allosaurus* fl	Cubic 2	I	13	87.2	11.4	13.0%
*Allosaurus* tc1	Quadratic 2b	I	16	23.9	1.80	7.5%
*Allosaurus* tc2	Power 2	I	16	21.4	1.91	8.9%
*Psittacosaurus m*1	Quadratic 2b	I	9	21	3.00	14.3%
*Psittacosaurus l*1	Persistence 3a	I	0.5	3	3.16	105.3%
*Psittacosaurus l*2	Persistence 3a	I	0.5	3.1	3.19	103.0%
*Psittacosaurus l*3	Persistence 3a	I	0.5	3	3.09	103.1%
*Psittacosaurus l*4	Persistence 3a	I	0.5	3.1	3.13	101.0%
*Hypacrosaurus* fc	Extreme Value 2	A	7	33.6	2.65	7.9%
*Hypacrosaurus* tc	Extreme Value 3b	S	6	23.6	3.16	13.4%
*Hypacrosaurus* tc	Extreme Value 3a	S	6	23.6	3.16	13.4%
*Apatosaurus*	Rational 2z	A	5	4.64	0.79	17.1%
*Apatosaurus*	Michaelis Menten 2	A	5	4.64	0.79	17.1%
*Alamosaurus*	Linear 2	I	13	12.4	0.78	6.3%
Northampton	Persistence 3a	I	5	62	2.10	3.4%
*Janenschia*	Power 3	I	5	26.5	2.32	8.7%
*Massospondylus*	Linear 2	I	15	44.46	2.52	5.7%

Bone growth rates were estimated by best-fit models (those for which 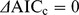). Growth rates are evaluated at the data point 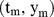 at which the maximum growth rate 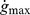 occurs. The growth rate is expressed both directly (cm/yr) and as percentage of size per year. S: sigmoidal; I: increasing; A: attenuating.

## Results

### Comparison with Prior Analysis

Table 3 presents the results of my attempt to replicate the published findings from previous studies of the growth of 26 of the distinct taxa listed in Table 1. For 15 taxa, the published reports did not include sufficient details (i.e. they were missing data, or detailed analytical methods, or regression equations), so they could not be verified directly. Replication was not attempted for one taxon (*Allosaurus*) because its situation is complicated, as covered in a section below.

Replication of the regression results to within 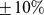 of the reported values for 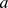 and 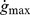 was possible for only four of the 11 taxa attempted. For the remaining seven taxa, least-squares regression yielded best-fit regression parameters that are substantially different from those reported, despite my using the same growth functions and analytical methods specified in the original studies (Table 3, Table S4, Table S5 and Table S10, Figs. S1–S7).

Where replication yielded substantial discrepancies, I used image-processing software to overlay plots of the reported data and regression curves onto digital scans of the original figures. In the case of *Syntarsus* and *Massospondylus* [20], the figures plot data points that differ markedly from their referenced sources [5], [6]. In the case of *Tyrannosaurus* and *Gorgosaurus* [25], the published regression equations do not provide the best fit to the data provided. For both *Tyrannosaurus* and *Psittacosaurus lujiatunensis* [18], the curves plotted in the figure of the original study match neither the published regression equations nor the best-fit regression equations that emerge from my calculations. Moreover, the data points plotted in the figure for *Psittacosaurus* do not match the published data set. My attempts to reconcile these discrepancies by testing possible sources of error were unsuccessful.

In the case of a growth study of *Alamosaurus, Apatosaurus, Janenschia* and the Northampton sauropod [37], my regression results differed slightly from those reported. In that paper, however, the authors do not present the equations as best fits from regression, but rather as upper and lower bounds, and my results confirm that the equations do indeed serve as approximate bounds on the best fits.

A further replication issue is that calculation of the maximum growth rate from the published regression equation could not be replicated for any of the 10 taxa in [20], [25]. The issue of irreproducible results in prior studies is explored in more detail in Text S1, Table S5 and Table S10 and Figs. S1–S7. In several studies, the resulting maximum growth rates are then converted from units of kg/year to grams/day to facilitate comparison to each other and to data from extant animals [20], [24], [25].

Unfortunately, this conversion is done by dividing the annual growth rate by the number of days in a Mesozoic year. This would be correct if the length of a Mesozoic day was, in absolute time units such as seconds, the same as a present era day. Instead, it is the duration of a year that is constant across geologic history [45], while the length of a day (and thus the number of days per year) has varied due to changes in Earth’s rotation rate [46]–[49]. As a result, the dinosaur daily growth rates as calculated are inappropriate for comparison to rates from extant animals or to each other if they are from different geologic periods. In addition, there appear to be other issues with some of the daily growth rate calculations, see Text S1.

### Impact of Choice of Independent Variable

Those estimates made by using age as the independent variable had a standard deviation that was 208% of the standard deviation for estimates derived from the 500 regressions that used age as a dependent variable for heteroscedastic errors and 409% for homoscedastic errors. (Text S2 and Fig. S9). These results demonstrate that the incorrect choice of independent variable can result in highly error-prone estimates.

### Impact of Choice of Model

**Figure 3:**
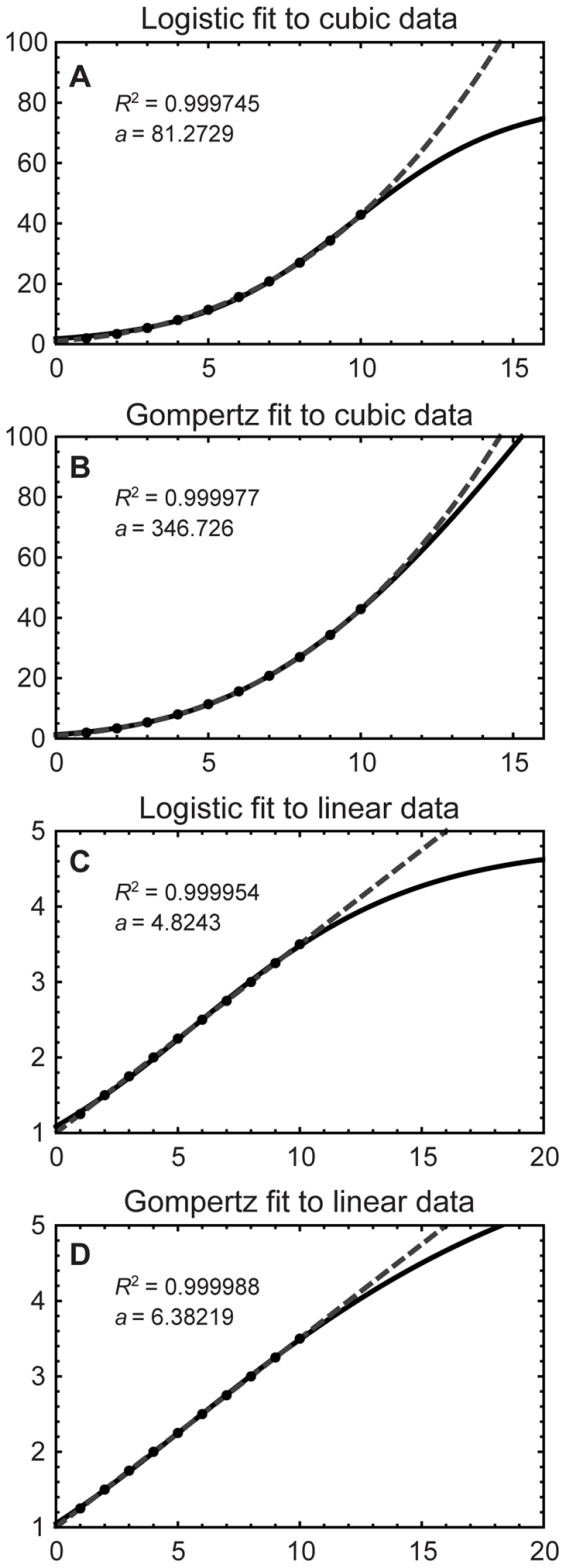
Logistic and Gompertz curve fits to synthetically generated cubic and linear data sets. Sample plots show logistic and Gompertz curve fits (solid curves) to a synthetically generated cubic data set (**A**, **B**) and linear data set (**C**, **D**), shown as dashed lines. Note that 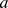, the asymptotic value of the best-fit curves as 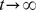, differs substantially between the logistic and Gompertz fits, despite 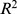 values near 1 in each case.

Asymptotic curves contain phases that are increasing and nearly linear. As a consequence, asymptotic curves can almost always be fit to a finite number of data points sampled from linear, cubic or other increasing curves sufficiently closely to obtain a value of 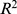 approaching unity [43], [50]. This is illustrated by Fig. 3, which shows logistic and Gompertz model fits to linear and cubic data. Fits of the 62 asymptotic growth curves in this study to the same linear data all yield 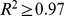; fits to the cubic data from Fig. 3 yield 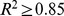 in all but four cases (Table S3). The resulting model fits produce estimated maximum asymptotic sizes that range (in the linear case) from 2.9 to more than 8.46×10^55^ (Table S3 and Text S4). Since there is no maximum asymptotic size for the linear data set, these are mathematical artifacts caused by using the wrong model to fit the linear data.

A Monte Carlo simulation comparing fits of asymptotic models revealed that their standard deviations varied widely (Text S3 and Fig. S9). The choice of model clearly matters a great deal to the final results. But the right choice cannot be known *a priori*, so it is important to test many models and to use an objective criterion, such as 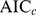, to select the best-performing model.

### Impact of Error Analysis

**Figure 4:**
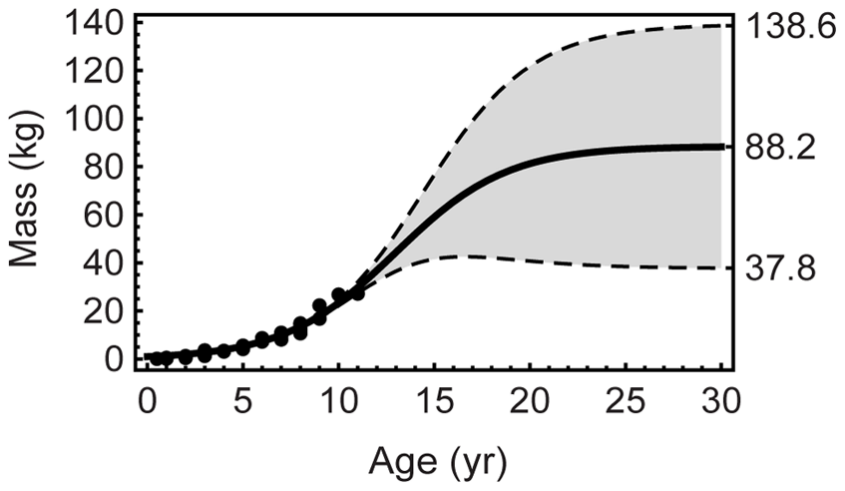
Logistic fit to *P. lujiatunensis* age–mass data points. The 95% confidence band for the logistic curve is shown in gray. Despite a large number of data points (82), the data poorly constrain the asymptotic portion of the curve because they represent mostly young specimens. By age 30, the curve has reached 88.2, which is nearly the maximum asymptotic size for this curve fit of 88.47. The fit shown here is based on an attempted replication of the results in [18]–see Text S1.

The 95% confidence band is a standard statistical error analysis technique that graphically shows the limitations of predicting future values from small data sets. Fig. 4 illustrates my attempted replication (see Text S1 and Fig. S7) of the model fit for *P. lujiatunensis* from [18] (maximum asymptotic size 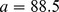 kg and 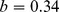) along with the 95% confidence band (CB) for the model fit.

The oldest specimen in the data set is age 11, and the size of that specimen is just 31% of 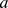, as estimated by this model. This ratio serves as an internal consistency check on the model fit and its likely accuracy–it tells us that the predicted maximum asymptotic size occurs far from the data, at more than twice the age of the oldest specimen. As a result, the CB closely follows the data points, but as the curve leaves the region it broadens substantially, ultimately spanning a factor of 3.7.

In this case, we know that the underlying osteology data set was explicitly shown to be linear [18]; we should not expect this logistic model to be valid in this case, any more than fitting to linear data (cf. Fig. 3 and Table S3). Unfortunately, the utility of confidence bands or intervals is limited by the fact that we do not know the correct error model. The confidence band of Fig. 4 may be conservative because it assumes a homoscedastic error model. If instead the errors are heteroscedastic–as is discussed below–then the CB could be even wider. A similar limitation occurs with confidence intervals – as the bootstrap and Monte Carlo error analyses show, it is very easy to find that even small amounts of error can swamp the signal and lead to infinite confidence intervals. Even with these limitations, it is instructive to perform error analysis.

### Best Fits and Growth Rate Estimates from the Reanalysis

**Figure 5:**
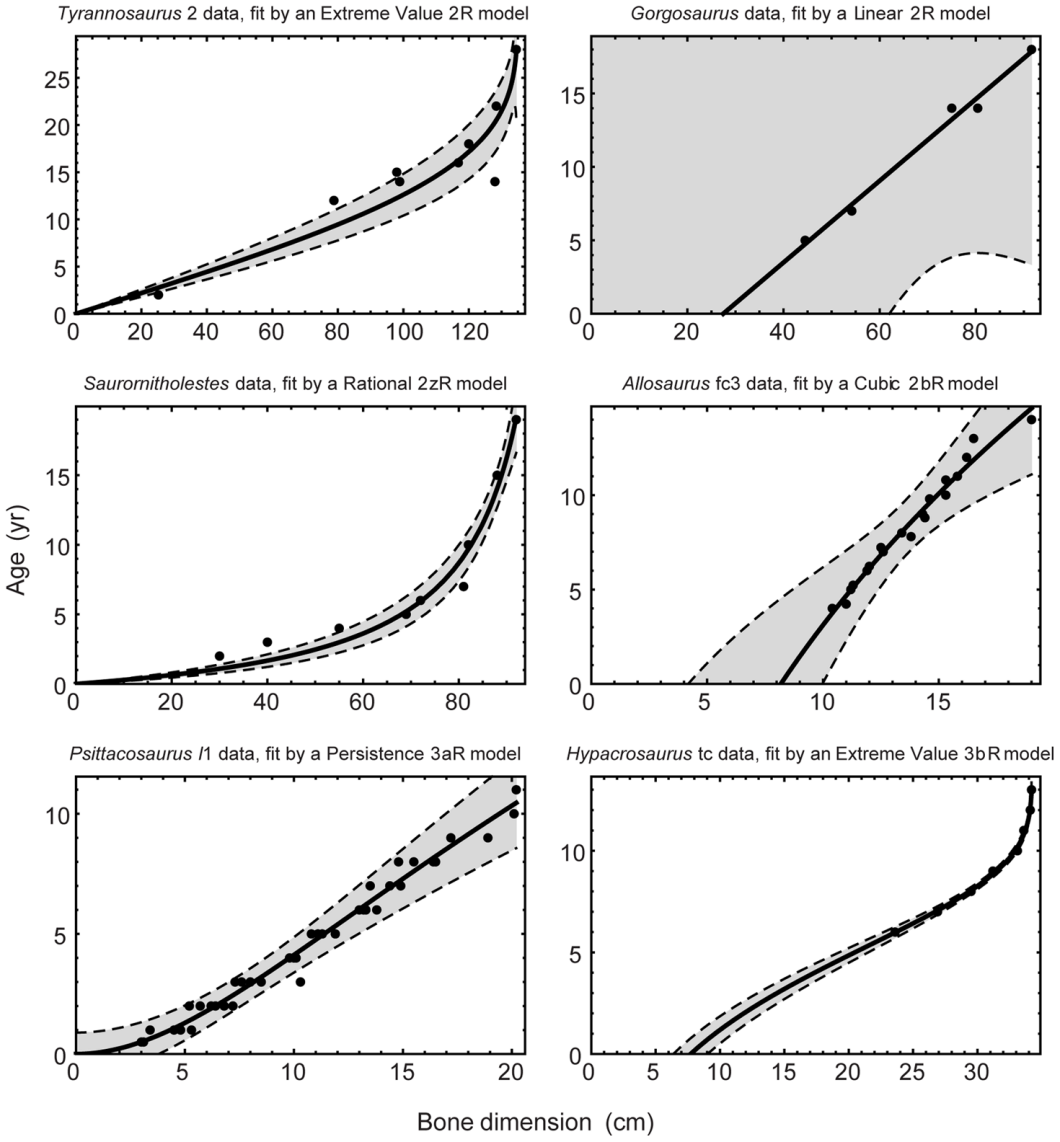
Best-fit growth curves for selected dinosaur taxa. Curves are the results of non-linear regressions in which bone dimension was used as the independent variable and age as the dependent variable. In each case, the curve shown provided the best fit to the data (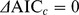) among 77 alternative models. See Table S8 for fit parameters and Fig. S12 for plots of best-fit curves for other taxa. The shaded area is the 95% confidence band, assuming homoscedastic, normally distributed errors.

Table 4 presents the best-fitting models and estimated maximum growth rates for the 31 data sets; a subset of these curves is illustrated in Fig. 5. Asymptotic models are best fits (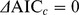) to all data sets for only four taxa: *Tyrannosaurus*, *Saurornitholestes*, *Hypacrosaurus* and *Apatosaurus*. The best fits to the remaining 10 taxa were obtained either from increasing functions alone or from a mixture of asymptotic and increasing curves across multiple data sets. Table S2 and Table S8 present results for all models having strong statistical support (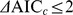), as well as best fitting Attenuating and Sigmoidal curves if none have 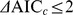. Fig. S12 plots the best-fit curves for all of the taxa.

The estimates of 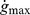 listed in Table 4 are model-dependent; alternative estimates produced by fitting other models are given in Table S4. Because all models must fit the same data points for final sizes, different models all generate the same average growth rate for a given data set.

When expressed in relative terms, the estimated growth rates range from a low of 3.4% per year for the Northampton data set to a high of 105% per year for the *Psittacosaurus l*1 data set. Although the latter figure indicates an annual doubling in size, that pace of growth seems reasonable when one considers that it occurs at the hatchling stage when the femur length is just 3 cm.

### Assessing Specimen Maturity

For the four taxa noted above that are best fit by asymptotic curves, with 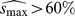, three of them (*Tyrannosaurus*, *Saurornitholestes* and *Hypacrosaurus*) have 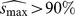 indicating that the data sets include specimens that at least approach skeletal maturity. But for the remaining ten taxa, the oldest specimens available are less than 62% of the asymptotic size predicted by their best-fitting asymptotic models. This finding suggests that the specimens for those taxa were skeletally immature at the time of death, and that the asymptotic fits may suffer from insufficient data.

**Figure 6:**
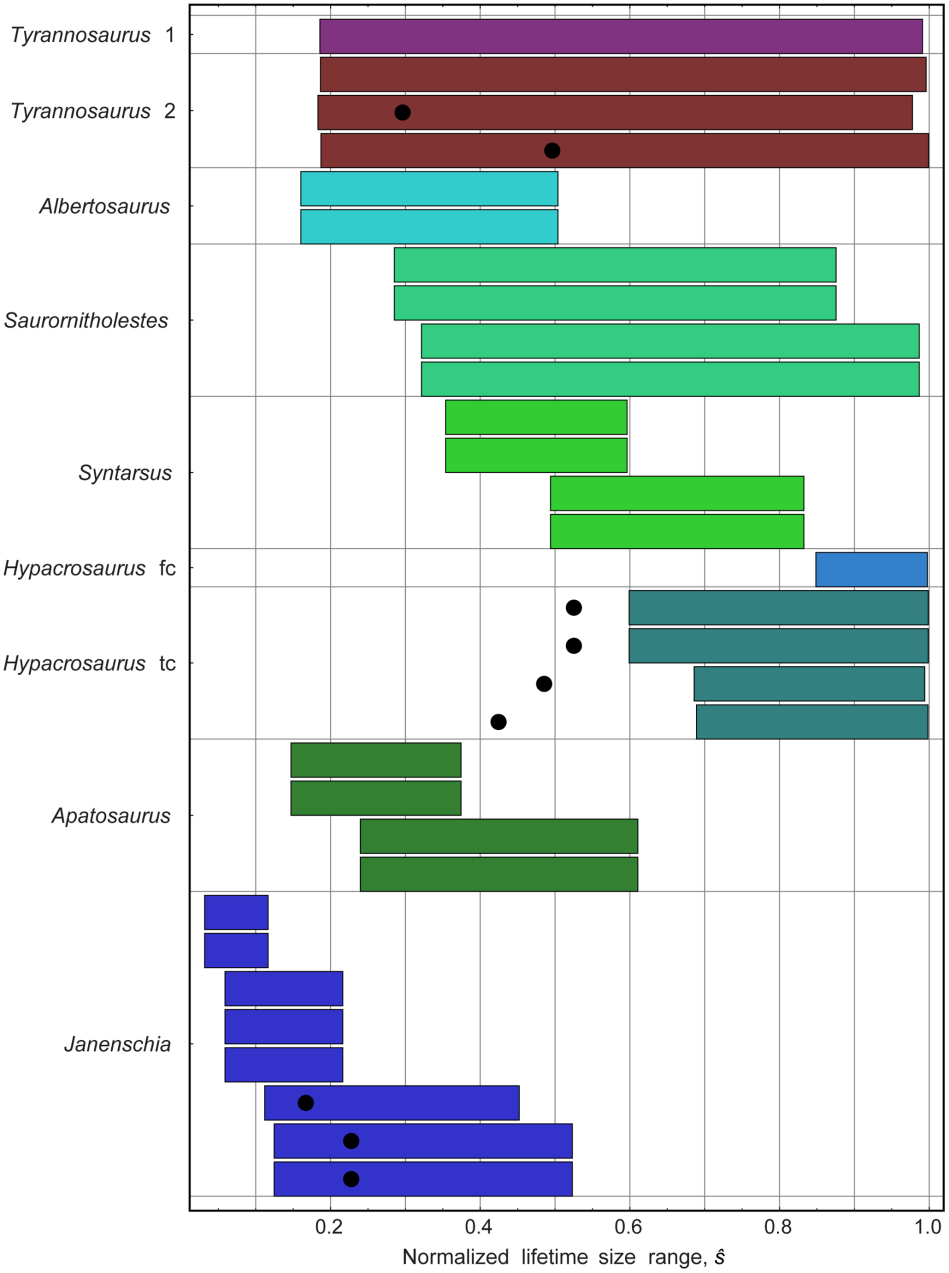
Ranges of bone sizes reported in asymptotic models. Each horizontal bar shows the range of values in the data set, expressed as a percentage of the maximum bone size predicted by each asymptotic curve that strongly supports the data set. (For some data sets, the best-fitting asymptotic curve may not fit as well as an increasing curve; see Table 4.) The best fitting asymptotic model is displayed at the top for each taxon. In cases where a sigmoidal curve provides the best fit, a round dot indicates the rescaled size 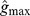, at which the maximum growth rate occurs. Each range shown depends on the model selected, because the curve fit determines the values of 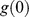 and 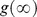 that are used in rescaling. Note, for some taxa, 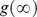 is so large that horizontal bars showing the range in values of the data set are imperceptible, and therefore have been omitted, as for *Psittacosaurus mongoliensis*. Data for these taxa can be found by comparing Table S6 and Table S8.

Fig. 6 shows the range of the rescaled sizes 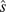 and the location of 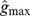 for all data sets in this analysis for which an asymptotic fit shows strong support (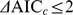, see also Table S8). The breadth of the range of sizes and the degree to which the span covers sizes approaching 100% provide a basis for evaluating confidence in the maximum size estimated by the model. For *Tyrannosaurus*, for example, the fact that the sizes range from about 18% to 99% of maximum size suggests that the best-fit asymptotic model is applicable for most of the life span of this taxon and the predicted maximum size is likely a reasonable estimate. We can similarly draw confidence in the maximum sizes predicted by model fits for *Hypacrosaurus* and *Saurornitholestes* because their data fall well within the asymptotic region, and thus constrain the model there. Because the two *Hypacrosaurus* data sets cover only the portion from 85% to 99% for the femur data set, and from 60% to 99% for the tibia data set, however, the estimates they yield for maximum growth rate or other metrics that occur early in the lifespan are not well constrained. *Psittacosaurus mongoliensis* has been omitted from Fig. 6, because its largest specimen is only 0.6% of the very large predicted asymptotic size, so its bars would not be visible on the chart. Some *Allosaurus* bone data sets have an asymptotic model with strong support, but its special situation will be discussed below.

In the case of *Psittacosaurus l*1 through *l*4, there were no asymptotic models with strong support. Nevertheless, the largest specimens are at most a mere 15% of the predicted maximum adult size from the best fitting asymptotic models. The best interpretation of this result is that we should have little confidence in the estimate of maximum size produced by the asymptotic models in these cases, both because those models require a large degree of extrapolation (*i.e.*, from 15% to 100%) to estimate an asymptote, and because they do not fit the data as well as increasing curves do.

Fig. 6 shows that most of the dinosaur data sets similarly span a small part of the apparent size range for the taxon–for eleven sets, data are missing altogether for the later growth stages–and that even among those taxa for which an asymptotic model does garner strong support, most of the specimens (including some of the largest specimens) were skeletally immature at the time of death. Underrepresentation of fully grown specimens in the data sets could explain why increasing growth functions are a better fit than asymptotic models for so many taxa. Other possible explanations are discussed below.

### The Effects of Population Sampling

In 26.8% of the 500 bootstrap trials of the *Tyrannosaurus* 2 data set, an increasing curve was the best fit to the resampled data; in contrast, only asymptotic curves obtained strong statistical support when fit to the original data set. The reason is clear from examination of the data sets: the original data include only one very old specimen (age 28) and one very young specimen (age 2). If either or both of these data points are missing from a bootstrap trial, the data points in the middle may be best fit by a non-asymptotic function.

Bootstrap sampling can be used in certain cases to calculate confidence intervals on parameters of interest such as 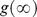, at least with respect to population sampling errors. Unfortunately, that approach does not work in this case because some of the best fits are by increasing functions, for which 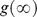 is infinite. This method could be used to create error estimates and confidence intervals for sizes and ages which fit within the range of the data points, but those would still be very large.

This example shows that population sampling will continue to be a problematic issue for dinosaur growth rate studies until the data sets include a sufficient number of points in all parts of the life cycle to enable stable statistical results. This limits the statistical power of estimates, including those in this paper.

### The Effects of Errors in Age Estimation

A Monte Carlo simulation using normally distributed heteroscedastic errors in age added to the *Tyrannosaurus* 2 data set found that, whereas only asymptotic curves had strong statistical support when fit to the unadjusted data set, the best-fit model was an increasing function in 1% of the trials when 5% error was added to the age estimates. At 10% error, the proportion rose to 12.6%, and it increased further to 19.8% when 15% random error was added to the data. This is in good agreement with the result found for Monte Carlo simulation on the effects of error on a synthetic data set (see Figure S10 and Text S5).

This result suggests that even relatively small amounts of age estimation error can alter the pattern in the data so much that an asymptotic curve no longer offers the best fit. As with the bootstrap results discussed above, this is almost certainly because the asymptotic nature of the data set depends crucially on a few data points; if the error on those particular points is great, the correlation to asymptotic growth suffers disproportionately.

## Discussion

Each of the steps in the whole bone, or longitudinal approach to growth analysis involves assumptions and choices in the method of analysis – some of which are explicitly stated, others appear implicitly. The success or failure of the overall growth analysis depends crucially on these assumptions and choices.

### Issues with Age Estimation

LAG counts involve at least two kinds of uncertainty: inconsistent counts and missing LAGs. Counts sometimes vary among bones taken from the same specimen, and even among multiple thin sections cut from the same bone [10], [51]–[53]. In addition, LAGs may be missing as a result of bone remodeling, particularly remodeling that occurs during enlargement of the medullary region in the bone cortex [10], [15].

In the whole-bone method, retrocalculation (step 1a) is sometimes used in an attempt to reduce both kinds of uncertainty. The most common approach identifies the smallest distance between LAGs found in smaller specimens, and then uses this single value to estimate LAGs missing from the bones of larger specimens [5], [6], [20], [24]. As Fig. 1 illustrates, however, the minimum LAG distance can vary among specimens. The whole-bone method lacks the additional data required to reconcile inconsistent LAG counts or thicknesses.

Retrocalculation has also been performed by physical superposition of thin sections [18] rather than by calculation. Important details of the retrocalculation method used–such as how to reconcile conflicting data from multiple specimens–are omitted or poorly documented in most papers, as are intermediate data required to replicate the calculations [10], [20], [24], [25], although some studies [11], [40] have reported such data.

In the longitudinal method, the correlation of time series (step 2) is able, in principle, to provide more robust results when LAG counts are inconsistent or missing because it can combine times series from multiple specimens to provide coverage of the entire growth range. Fig. 1 illustrates the application of this process to the *Allosaurus* humerus data from multiple specimens spanning a wide range of sizes by Bybee *et al.* [32]. Their approach is to add age offsets, specified as an integer number of years, to each time series to align the curves by sight. Woodward [35] and Lehman and Woodward [37] similarly analyzed data on two specimens, while Lee [36] applied the method to several taxa. However, most longitudinal studies have examined only single specimens, which limit estimations of the absolute ages to educated guesswork.

### Issues with Mass Estimation

Osteology is the source of all data on dinosaur growth, and questions about maximum asymptotic size (if any) and other biological characteristics can and should be answered directly from observations of bones. If estimated masses are needed, they would best be calculated only after statistical analysis has been performed on the osteological growth data. Performing mass estimation as an intermediate step prior to fitting a growth curve unnecessarily risks introducing error, which could confound the statistical analysis challenges already present in the osteology. Nevertheless, many dinosaur growth studies have estimated body mass from bone dimensions as an intermediate step. Three methods are in use for mass estimation. Each involves uncertainties or issues that remain contentious.

#### The Anderson method

The most widely used method was described by Anderson *et al.* [34], who expanded on an idea proposed by Alexander *et al.* [54], [55] that body mass correlates with the mechanical strength of long bones, which is in turn a function of bone circumference. Anderson *et al.* calibrated this relation by using an empirical regression on extant quadrupedal mammals ranging in weight from 47 g to 6,000 kg. The resulting formula for bipeds is
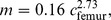
and for quadrupeds the formula is



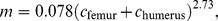
where 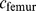 and 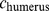 are the circumference of the respective bones in mm, and the mass, 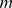, is in grams.

A recent study [56] of 200 mammal and 47 non-avian reptile species found substantial empirical support for the Anderson *et al.* method with a slightly different exponent (2.749 rather than 2.73). The relevance of the Anderson method to dinosaurs and its performance as a mass estimator is a vigorous research area and is the subject of much past and ongoing work [57]–[67].

#### Developmental mass extrapolation

DME was introduced by Erickson and Tumanova [24] on the rationale that the method of Anderson *et al.* is suitable for estimating the mass of an adult dinosaur, but cannot be used for juveniles. Erickson and Tumanova offered no criteria for determining the age or degree of maturity of specimen for which the Anderson method is applicable, however, nor have they or others provided reason to believe that, *a priori*, the Anderson method is any more or less suitable for juveniles than for adults.

As practiced, DME requires the assumption that total body mass scales isometrically in bone dimension (at least for the bones measured), from neonate to maximum adult size [24]. Indeed, a more descriptive term for the method would be isometric interpolation. Isometric scaling implies that the body mass, 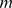, scales as 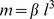, where 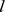 is a linear bone dimension (length, radius, or circumference), and 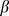 is a constant that has the same units as density. Typically, the bone dimension used in DME is femur length, but if the assumption of isometric growth holds true, then any linear bone dimension should have the same correlation, although the value of 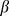 may differ. To use DME, one first selects the specimen with the longest femur length 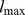, and makes a mass estimate for that specimen 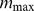 via Anderson or some other method. Given that, the mass for other femur lengths 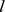, between 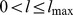, is given by
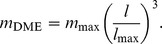



In the case of isometric growth, DME yields estimates for mass for the ontogenetic series that are smaller than those generated by the Anderson formula, by the ratio
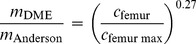
where 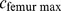 is the circumference of the longest femur (*i.e.*, the bone used to measure 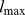 in step 2b of the whole-bone method).

Most allometric correlations for determining body mass are verified by extensive comparisons to extant animals [34], [56], [57], [61], [65]–[72]. Despite an extensive search of the literature, I was unable to identify any published work that assesses the performance of DME by applying it to ontogenetic series for extant animals.

In three articles [20], [24], [25], Erickson and coworkers cite studies of the alligator *Alligator mississippiensis* and the California gull *Larus californicus*, as well as a 1947 study of *Homo sapiens.* These studies did not apply DME but instead showed support for more general isometric growth during ontogeny. The relevance of humans as a test case for dinosaurs is unclear. More recent studies [73]–[77] find that human growth during childhood is not isometric, and in particular, is not isometric in femur length [70].

The question of whether dinosaurs grew isometrically can be investigated directly by studying bone dimensions in an ontogenetic sequence. Bybee *et al.* [32] showed that growth was not isometric for *Allosaurus*; each of the principle bones under study grew at a different rate. I tested this question in my reanalysis of the *Allosaurus* data. Kilbourne and Makovicky [78] examined allometry among bones in many genera of dinosaurs and concluded that, although a number of taxa did exhibit isometric growth, the large theropods in their study did not. This finding raises questions about the application of DME to the theropods in the studies listed in Table 1.

#### Laser scanning of skeletons

A third, emerging approach to estimating mass uses laser scanning of skeletons as a basis for 3-D computer reconstructions of soft tissue [58], [79]–[81]. Hutchinson *et al.* [41] applied this method to an ontogenetic series of *Tyrannosaurus rex* specimens. Their results show that the growth of *T. rex* was not isometric: body mass 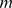 scaled with bone dimension 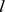 as 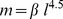 or 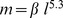 for the specimens in their study and thus is not well modeled by DME. Body masses for the youngest specimens, as estimated by DME, are 150% to 300% of the estimates derived by laser scanning (see Text S6, Table S9, and Fig. S11).

### Issues with Fitting Growth Models

The growth of dinosaurs is modeled mathematically, as in other areas of biology, by a function 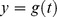, where 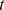 is age and 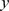 is a body size parameter, such as length or mass. Many functions have been used to describe biological growth (see Table S1), including increasing curves that grow without limit (*i.e.,* 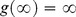)–such as linear, quadratic, cubic, exponential and power law curves–and asymptotic curves that approach the horizontal asymptotic value 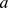 as 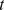 becomes large, *i.e.,* 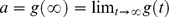.

Dinosaur growth studies have most often used logistic, Gompertz and von Bertalanffy curves, all of which are sigmoidal (S-shaped) curves. Sigmoidal curves have an inflection point in the linear phase at age 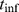 where 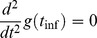 and the growth rate achieves a maximum value 
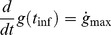
. Some dinosaur growth studies have used attenuating curves, such as the negative exponential (also known as monomolecular) curve 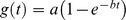. Attenuating curves include linear and asymptotic phases but lack an inflection point or an initial exponential phase.

A review of the literature revealed two broad classes of problematic issues in the ways that mathematical models have been applied in dinosaur growth rate studies: four conceptual issues having to do with unproven or incorrect assumptions that underlie many dinosaur growth studies and affect the interpretation of growth models, and five issues concerning the use of inappropriate statistical methods in the analyses of the models. Each of these issues is discussed at length here so that these problems may be addressed and avoided in future work.

#### Assumption 1–Determinate growth and sigmoidal growth curves

Most previous studies assume that dinosaurs (or all vertebrates, according to some authors) exhibit determinate growth, and moreover that individual specimens must thus have followed sigmoidal growth trajectories. This assumption is often stated directly (see *e.g.* [20], [24]–[26]).

The term “determinate” growth has been applied widely, but often very loosely, in biology. Sebens [82], in a comprehensive review of biological growth patterns, defines four types of determinate growth. Type 1, the growth pattern that is implicitly assumed by most studies of dinosaurs, is genetically predetermined, with the result that all individuals in the species share a common growth curve that is asymptotic (but not necessarily sigmoidal). Type 2 determinate growth also follows an asymptotic curve at the individual level, but the maximum size and growth rate may differ from one habitat to another for a given species, even when genetic changes are absent. In aggregate data on multiple specimens, this variance in growth appears statistically as error in the age estimate. In such cases, the aggregate data may sometimes be fit best by an increasing function rather than by an asymptotic curve, as is easily demonstrated with Monte Carlo simulation (see below and Text S5). If type 2 determinate growth applies to dinosaurs, then we should expect that a data set including individuals from different habitats and different time periods may or may not show asymptotic growth.

In type 3 determinate growth, mortality rates vary by habitat but are sufficiently high that few or no individuals reach an asymptotic size. Except in permissive ecosystems (for example, in captivity), individuals continue growing, perhaps at decreasing rates, until death. Sebens points out that growth curves in type 3 determinate growth may not be asymptotic, even at the individual level. Type 4 determinate growth combines the properties of types 2 and 3, with high mortality and variation at the habitat or individual level. Again, the growth trajectories need not follow an asymptotic curve.

Some dinosaur specimens [25] exhibit an external fundamental system (EFS), a tightly spaced set of LAGs that is thought to mark the cessation of further growth [8]–[12], [15]. The existence of an EFS implies that the specimen stopped growing, either because the individual reached skeletal maturity or as a result of disease or habitat-specific reasons, as can occur in type 1 indeterminate growth within Sebens’ taxonomy. The cessation of growth, even if it is a result of skeletal maturity, does not by itself provide conclusive evidence of whether the animal experienced type 1, 2 or 4 determinate growth, or whether the growth curve was asymptotic.

Longitudinal studies of the growth of individual specimens offer some examples of growth cessation. The *Hypacrosaurus* data set studied here corresponds to a single individual that apparently reached skeletal maturity, and the slowing of growth observed in the data set is well fit by asymptotic curves. Yet we lack the information needed to determine which of the several possible types of determinate growth was at work. Nor does a result for a single hypacrosaur specimen imply that all dinosaurs grew as it did.

An analogous situation is posed by growth studies of *Alligator mississippiensis*: a large-scale (∼2000 data point) growth study [83] of wild specimens in the Florida Everglades found strong statistical evidence of an increasing growth curve, which the authors interpreted as evidence of indeterminate growth, consistent with other crocodilian growth studies [84]–[86]. Despite this, a recent histological study [87] found the presence of EFS in several captive *A. mississippiensis*, indicating that, at least in those specimens living in permissive environments, cessation of growth occurred well before death, which is generally interpreted as evidence of determinate growth.

Although a full analysis of growth in *A. mississippiensis* is beyond the scope of the present study, Sebens’ growth framework offers several possible resolutions to the apparent contradiction: the Everglades habitat may not allow specimens there to reach skeletal maturity (*i.e.*, type 3 or type 4 determinate growth may apply), or growth may be asymptotic but highly plastic (type 1 indeterminate growth). Further work is necessary to resolve this, but it demonstrates clearly that observation of an EFS does not necessarily imply that an asymptotic growth curve will fit growth data. Since this occurs for a well-studied extant animal, we cannot assume the situation will be better when interpreting the much smaller and less-controlled dinosaur data sets.

Finally, a substantial body of literature holds that many vertebrates undergo indeterminate growth [82], [88]–[91], including crocodiles [92]–[99], which are close dinosaur relatives and form one significant out-group in the extant phylogenetic bracket method [100]. It is thus far from proven that all vertebrates have determinate growth or sigmoidal growth curves. For these reasons and because dinosaurs are a large and diverse group, the hypothesis that some may have had indeterminate growth is worth testing.

#### Assumption 2–finite population samples

Many studies implicitly assume that growth data follows sigmoidal growth curves even when the overall population is sampled by a relatively small number of data points, and that therefore exclusively sigmoidal models should be fit to growth data. This assumption is refuted by the simple mathematical consequence of finite sampling. A finite data set sampled from a population may be best fit by a non-asymptotic curve–even if individual dinosaur specimens followed sigmoidal growth trajectories. This is easily demonstrated by Monte Carlo simulation (see Text S5). This is exacerbated by the presence of statistical noise in the data, which can swamp the signal, and cause the best fit to be an increasing function.

#### Assumption 3–data set sufficiency

A third unjustified assumption implicit in many dinosaur studies is that all data sets–even those that are restricted in the range of ages represented–are suitable for estimating the basic parameters of growth, including the maximum asymptotic size 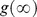. It is well known that fits to sigmoidal models yield meaningful parameters only if the data set being fit includes points that span the three distinct phases of the sigmoid curve (or the two phases of attenuating asymptotic curves) [101]–[103]. I used several methods to test whether the data sets representing 14 dinosaur taxa were suitable for sigmoidal fits.

Attempting to fit an asymptotic model to insufficient data can yield results that are erroneous or even nonsensical, as illustrated in Fig. 3 and by Table S3 and Table S4. These wide-ranging estimates are artifacts of the mathematical models used; they offer no meaningful interpretation of the “asymptotic size” because the data were drawn from a line or cubic that have no asymptote. Although these fits work properly in the vicinity of the data points, they clearly are not suitable for extrapolation far from them [43], [94], [96], [101]–[103]–but it is in that distant region that the asymptotic behavior occurs.

It may be that dinosaur growth data are biased by high mortality at both ends of the age spectrum in ways that undermine model fits. Juvenile dinosaur fossils are rare, as has long been noted [104], perhaps as a result of juveniles being consumed whole by predators or scavengers [92], taphonomic effects [17], or geographic clustering of nesting sites.

Dinosaur survivorship curves have only recently been studied [27], [28] and must overcome some substantial statistical issues [19], but the results so far indicate an exponential decrease in survivorship for the oldest individuals, as is found for many extant animals [101]. As an example, the life table for *Psittacosaurus lujiatunensis* assembled by Erickson *et al.* [18] shows 97.4% mortality by age 12, at which point the specimens are only 30% of the maximum size estimated by fitting the model used in the original study [18] (see Text S1 and Table S4 and Table S5). Examination of extant bird colonies showed that they produce specimens from age zero to about half adult size, but rarely any of full size [93], and this may be a good model for dinosaur nesting colonies. If high mortality is typical, then small samples are likely to contain few, if any, fully mature specimens.

#### Assumption 4–the prediction fallacy

Many dinosaur growth studies produce a mathematical model that is based on one or more of the assumptions above and least-squares regression. The resulting curve is then used to estimate a maximum asymptotic size, maximum growth rate, or other parameters–in effect, to predict the growth history of the species over its full lifespan, even when few or no data points lie near the relevant portion of the growth curve.

No principle of statistical inference supports this practice. Least-squares regression and related statistical techniques minimize the difference between a model and the data points [94], [102], [103]; they thus evaluate the model only at the data points, and it is only near those points that the resulting fit can be useful and valid. Taking a portion of the curve that is far from the data points seriously as a prediction is widely considered to be unsupported extrapolation [43], [94], [96], [101]–[103]. A specific example of this fallacy is to assume that the parameter 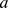 can always be interpreted as a good statistical estimate for 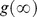, the asymptotic growth size. While parameter 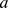 always exists for a fit to any data set, not every data set has sufficient points in the asymptotic region that one is justified in interpreting 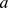 as a valid prediction for where growth will lead. That is the situation with the fits to linear or cubic data sets discussed above – the asymptotic fits are good in the vicinity of the finite data set, but not valid far away.

The best statistical practice is to use the model that best fits the data points, as measured by objective model selection methods [43], [94], [96], [101]–[103], rather than rely on one’s expectations for the behavior of growth trajectories. In this study, I used a formal model selection criterion, the corrected Akaike information criterion 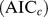 [43], to determine the best fits to data sets among a wide range of plausible models.

### Statistical Issues

Beyond these conceptual problems, five kinds of methodological issues appeared recurrently in the dinosaur growth rate studies I reviewed. In a number of cases, these problems reflect serious departures from accepted statistical methodology that could greatly magnify errors and may undermine confidence in the results of the studies, or even invalidate them altogether.

#### Inappropriate choice of variables

A bedrock assumption in ordinary least-squares regression (also known as Model I regression) is that the independent variable has no error; all error to be minimized by the regression procedure occurs only in the dependent variable [94]. Model II regression (or Deming regression) distributes the error equally in independent and dependent variables [94].

In dinosaur growth data sets, the bone dimensions are typically measured accurately to within a millimeter (except when fractured or incomplete), so they make a natural choice of independent variable for conventional regression. Age, in contrast, is estimated with considerably greater uncertainty, and for that reason it is the natural choice for the dependent variable. Using size rather than time as the independent variable has long been recommended in studies of growth in extant animals [95].

Unfortunately, most prior dinosaur growth studies treat age as the independent variable and use body size (either mass or bone dimension, depending on the study) as the dependent variable. This violation of the basic assumption of regression has the potential to introduce serious errors and to magnify the effect of any errors already present in the data.

Monte Carlo simulations show the severity of this wrong choice of independent variable – the error in parameter estimation is substantially larger. One previous dinosaur growth study [32] used Model II regression of age against mass in order to accommodate errors in both the mass and age estimates. An even better approach, in cases where tightly controlled laboratory measurements of bone dimension are available, is to avoid using mass estimates altogether, as they have unknown error properties.

#### Overfitting

The statistical explanatory power of a curve fit depends in part on a comparison of the number of model parameters to the number of data points. As the number of parameters approaches the data point count, explanatory power diminishes rapidly, and the data set is said to be overfit [105]–[108].

Growth studies for many dinosaur taxa are constrained by very small data sets (see Table 2 and Table S7). As a result, they require models that have a concomitantly small number of parameters. This requirement has not always been observed, however. Prior studies of three dinosaur taxa–*Shuvuuia*, *Maiasaura* [20] and *Daspletosaurus* [25]–employed four-parameter models to fit just three data points. In studies of *Apatosaurus* [20] and *Tyrannosaurus* [41], four-parameter models were fit to four data points. Such practices violate accepted statistical norms and cannot be expected to yield meaningful results.


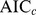
 assigns an information theoretic likelihood of validity that depends on 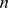, the number of fit parameters, and 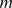, the number of data points. Zero likelihood of validity (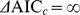) is assigned to fits where 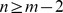 [43]. Under this criterion, the curve fits for *Gorgosaurus* and *Albertosaurus* [25], each of which fits four parameters to five data points, are also invalid. In cases of this kind, when the number of data points is known to be small, two- or three-parameter models must be used to obtain results that are statistically meaningful under 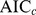. Overfitting cannot be avoided merely by selecting arbitrary values for one or more parameters (see Text S1).

#### Arbitrary model selection

Most prior studies of dinosaur growth attempted to fit only one model [20], [24], [25], [29], [36]. Just two [36], [38] papers reported the use of formal statistical criteria to select the best-fitting model among many plausible alternatives. All of the other studies reviewed here employed *ad hoc* or arbitrary means to choose among two or three growth models.

In a 2009 study [18], for example, the authors chose two sigmoidal curves (logistic and Gompertz) to fit age–mass data for *P. lujiatunensis*. Finding that “A Gompertz equation (black dashed line) shows similar fit for the empirical data but predicts an unreasonable asymptotic size (107-kg),” the investigators made a subjective choice to favor the logistic model, which better matched their personal judgment of what is reasonable for asymptotic size.

Such a reliance on intuition allows subjective opinions and preconceptions to enter and even dominate the statistical analysis. Limiting the models tested to just two excludes the possibility that searching a wider suite of curves may yield one that is a better fit. Moreover, model selection methods based on intuition are difficult to replicate and can generate contradictory conclusions. Fig. 3 in [18] shows that age is well fit by a linear function of femur length, for example, and the authors use this linear relation to estimate the ages of many of the specimens in the study. But they then use DME to convert this linear age–femur length relationship to an age–mass relationship, and they fit an asymptotic curve to the latter. Given that they show the data set is already well fit by a straight line, it is difficult to understand why only asymptotic models were applied to the data in the next phase of the study.

In contrast, objective model-selection methods such as 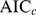 produce easily replicated estimates based either on the best-fit curve or on an 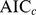-based weighted average across models [96].

#### Extrapolation without error estimates

Although the purpose of growth analysis is to estimate growth rate, maximum asymptotic size, and other biological parameters of interest, it is not always possible to derive good estimates from a limited data set. Unfortunately, confidence intervals, error estimates or other self-consistency checks are rarely presented in published studies of dinosaur growth. This omission limits the utility of the studies’ results, particularly for estimates of 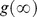 because, for 11 of the 14 taxa studied here, no data points fall within the asymptotic region.

#### Exclusive use of asymptotic models

Given the limitations of the data discussed above, it is a source of concern that most prior studies have excluded non-asymptotic curves when modeling growth data. This choice overly constrains the growth analysis and could result in important biological information being overlooked. It also opens up the possibility of erroneous estimates from fitting unsuitable models to the data.

The need to compare the performance of asymptotic models to non-asymptotic alternatives is particularly acute when estimating a biologically meaningful parameter such as 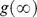, which does not exist in increasing models. Although, as the example in Fig. 3 shows, one can fit asymptotic curves to data derived from increasing functions, interpreting the fit parameter 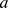 as an estimate for 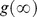 is invalid. The converse is also true; a finite data set sampled from an asymptotic curve can appear to be increasing. To be worthy of serious consideration, any estimate must be derived from a model that produces the best fit to the data (or at least has strong statistical support) among multiple alternatives, and even then it must only be used in the vicinity of the data points.

Although many studies feature exclusive use of asymptotic models, there have been notable exceptions. As early as 1993, Chinsamy-Turan [6] found that the best fit for *Massospondylus* was a power-law model, which was compared to exponential and other non-asymptotic curves. In a more recent study [15], she showed that growth data from *Massospondylus*, *Syntarsus* and *Psittacosaurus mongoliensis* fit non-asymptotic curves well. Several studies have applied power-law [32] or linear models [97]; Cooper *et al.* [38], for example, noted that a linear model also fits the data for several theropod taxa to which Erickson *et al.* [25] fit sigmoidal curves. In a 2007 paper, Lee [36] analyzed 26 datasets and found that 21 were best fit by a linear model. Some later studies [18], [27] nevertheless employed asymptotic curve fits exclusively for age–mass data, despite in one case [18] finding that linear models provided an excellent fit to the femur length–age data, which implies that growth was not asymptotic.

### Comparison to Previous Results

As discussed above and also in greater detail in the Supporting Information (Text S1), many previously published results in dinosaur growth studies were obtained by using methods that deviate from accepted statistical practices or are irreproducible due to lack of published data or methodology. Additionally, prior studies that presented mass growth rates depend crucially on estimates of soft-tissue mass, which are obtained by DME or other methods that involve intrinsically higher uncertainty than osteology. These prior studies also used age as the independent variable, whereas I use it as the dependent variable.

The study of bone growth offers a simpler and more informative source of insight into dinosaur growth rates, so I present bone growth rates (cm/year) in the tables and figures here as the preferred way to present results. However, I also used DME and the same mass estimates as the original papers to convert the bone growth curves into mass curves, but only for the purpose of comparison with previous work. Because this conversion results in mass–age data points that are the same as those published in prior work, my estimates of mean growth rate (*i.e.*, averaged over lifespan) must be identical to those in previous studies. Only the growth trajectories–*i.e.*, whether growth was slow and steady, or concentrated in a rapid burst–may differ. In particular, I find peak growth rates that differ, in many cases substantially, from those previously reported and widely cited in the literature (Table 3).

#### *Tyrannosaurus*

A 2004 analysis by Erickson *et al.* [25] estimated a peak growth rate of *Tyrannosaurus* of 767 kg/yr. This cannot be replicated; the first derivative of the regression function given in that paper actually peaks at 791 kg/yr – see Text S1 for a discussion. A 2006 study by Bybee *et al.* [32] estimated a growth rate for this species of 559 kg/yr, and in a subsequent paper, Erickson *et al.* [17] published a revised estimate of 601 kg/yr. The recent method of Hutchinson *et al.* [80] for estimating tyrannosaur masses has so far been used on only four specimens, too few to fit even a two-parameter model.

I was unable to replicate the published regression equation of [25]. The best-fit regression that I found using their original stated methodology yields a peak growth rate of 467 kg/yr (Table 3, see also Fig. S1, Fig. S6 and the discussion in Text S1). However, the more rigorous approach described above, which uses femur length as the independent variable and formal model selection among a wide set of growth functions, followed by conversion from femur length to mass, results in an estimated peak growth rate of 365 kg/yr (Table 5). This value is 22% less than that obtained by attempted replication of [25], but is less than half that of the peak rate derived from the equation published in that reference.

**Table 5:** Comparison to previous estimates of mass growth rate.

Taxon	Reference	Source of estimate	Age of data pointwith highestgrowth rate (yr)	Highestgrowth rate(kg/yr)	Highestgrowth rate(%/yr)
*Tyrannosaurus*	[25]	published result		767	
	[25]	derived from regression equation	16	790	29%
	[25]	actual best fit to age, mass	16	460	18%
	this paper	bone dim. fit, scaled to mass	14	365	15%
*Gorgosaurus*	[25]	published result		114	
	[25]	derived from regression equation	14	107	13%
	[25]	actual best fit to age, mass	18	126	11%
	this paper	bone dim. fit, scaled to mass	18	132	12%
*Albertosaurus*	[25]	published result		122	
	[25]	derived from regression equation	15	126	17%
	[25]	actual best fit to age, mass	15	121	16%
	this paper	bone dim. fit, scaled to mass	24	155	9%
*Massospondylus*	[20]	published result		34.6	
	[20]	derived from regression equation	12	33.3	19%
	[20]	actual best fit to age, mass	15	51.1	21%
	this paper	bone dim. fit, scaled to mass	15	42.30	17%
*Syntarsus*	[20]	published result		9.14	
	[20]	derived from regression equation	3	7.46	100%
	[20]	actual best fit to age, mass	4	5.41	52%
	this paper	bone dim. fit, scaled to mass	6	4.83	25%
*Psittacosaurus mongoliensis*	[20]	published result		5.82	
	[20]	derived from regression equation	7	5.43	52%
	[20]	actual best fit to age, mass	7	5.61	53%
	this paper	bone dim. fit, scaled to mass	9	9.37	41%
*Psittacosaurus mongoliensis*	[24]	published regression equation	7	4.59	41%
	[24]	actual best fit to age, mass	8	5.11	32%
	this paper	bone dim. fit, scaled to mass	9	9.37	41%
*Psittacosaurus lujiatunensis*	[18]	published regression equation	0.5	5.04	24%
	[18]	actual best fit to age, mass	10	5.24	22%
	this paper	bone dim. fit, scaled to mass	11	7.71	25%
*Apatosaurus*	[20]	published result		5466	
	[37]	derived from regression equation	18	399	11%
	[37]	actual best fit to age, mass	18	482	11%
	this paper	bone dim. fit, scaled to mass	17	485	11%
*Alamosaurus*	[37]	derived from regression equation	13	890	15%
	[37]	actual best fit to age, mass	13	1083	16%
	this paper	bone dim. fit, scaled to mass	13	1271	19%
*Janenschia*	[37]	derived from regression equation	11	624	15%
	[37]	actual best fit to age, mass	12	657	14%
	this paper	bone dim. fit, scaled to mass	20	1430	10%
Northampton	[37]	derived from regression equation	24	180	7%
	[37]	actual best fit to age, mass	33	209	5%
	this paper	bone dim. fit, scaled to mass	25	281	4%

Note that the growth rates in this table are only evaluated at the data points, which have integer ages. The highest growth rate at a data point is less than or equal to the maximum growth rate across the entire life span 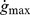, which in general occurs at a non-integer age 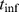.

Comparison of maximum mass growth rates and age at peak growth rate from the best-fit models in this study (those for which 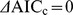) to corresponding values published in other studies. Growth rates are expressed in terms of both mass and percentage of mass added per year.

It is unclear whether the simple Monte Carlo error model used here adequately describes the errors likely to occur when estimating dinosaur ages. Horner and Padian [40] published error estimates with their *T. rex* ages of 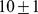, 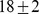 and 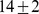, which correspond to 10% to 14% error. Few other growth studies have reported error estimates. If the Monte Carlo model presented here does adequately reflect the errors, the simulation results imply that the *Tyrannosaurus* 2 growth curve is sensitive to estimation error and that further work is needed to determine what value or distribution of errors are appropriate.

#### *Apatosaurus*

A 2001 study [20] estimated a peak growth rate of 5466 kg/yr for *Apatosaurus*. The results here find a far lower rate of 485 kg/yr, which supports the conclusion of other authors [35], [37] that the earlier estimate was flawed, although the disparity may also arise in part from differences in the data used. My analysis draws on Woodward’s longitudinal reanalysis [35] of two of the specimens reported by Curry [98]. The 2001 study used four specimens from Curry to create a whole-bone data set, but those data were not available.

In all three previous studies of *Apatosaurus* growth rates, the authors fit asymptotic (von Bertalanffy) models exclusively. My results do show strong statistical support for asymptotic models of this data, but the estimates of maximum asymptotic size should be treated very cautiously because, under the best-fit model, the largest specimen is only 37% of full size.

#### Other dinosaur taxa

Results for other taxa are summarized in Table 5. Reported rates in this table are limited to just the eleven taxa in those five studies (out of the 26 listed in Table 1) for which the data and details published were sufficient to reproduce the regression results closely.

Table 5 presents growth rates from both asymptotic and increasing functions. These rates can be compared because they have been evaluated only at data points, thus avoiding issues of extrapolation. In general, the results here are quite compatible with those from previous studies, and the differences show no overall pattern: in some cases the best-fit rates are faster, and in others they are slower.

In the case of *Alamosaurus*, the earlier study estimated an asymptotic size, whereas I find that the only model having strong support is linear and thus does not approach an asymptote. I similarly find strong support only for an increasing model to fit the data set for the Northampton sauropod. The data for *Janenschia* support both increasing and asymptotic models, but the range of sizes in the data set does not extend far enough into the asymptotic growth region to allow estimation of the asymptotic size with any confidence.

A reanalysis of the *Psittacosaurus l*1 data set from [18], using femur length as the independent variable, found that the data is in fact best fit by an increasing model. Moreover, even when the reanalysis is restricted to asymptotic models, logistic and Gompertz curves do not garner the greatest support from 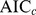.

Quantitative comparison of the mass growth rates estimated here to those made in longitudinal studies other than those mentioned above is not possible because they do not explicitly calculate mass growth rates. Those studies instead use growth rates to draw conclusions about differences in ontogenetic growth between bones of *Allosaurus* [32], or between *Tyrannosaurus* and *Hypacrosaurus* [38]. The qualitative conclusions of these studies are supported by the analysis here, as is the finding of non-isometric growth in Bybee *et al.* [32], which provides the best longitudinal data set available for any taxa of dinosaur.

### The Case of *Allosaurus*

Bybee *et al.* [32] presents multiple data sets for *A. fragilis*, including longitudinal data measured from humerus, tibia, ulna and femur fossils. Curves were fit by eye, using integer age offsets, to determine the ages (Table S6, data sets labeled hc1, tc1, uc1, and fc1, respectively). I reanalyzed the data by using a least-squares minimization procedure that does not constrain the age offsets to integer values. The results (Table S6, data sets labeled hc2, tc2, uc2, fc2, fc3 and fc4) are highly similar to those previously reported [32] for the humerus (Fig. 1), ulna, and tibia.

**Figure 7:**
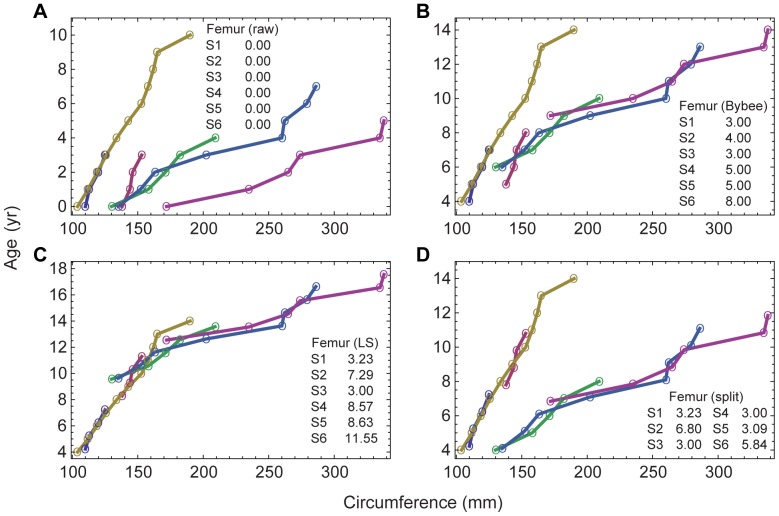
Longitudinal time series for *A. fragilis* femora. **A**, raw data from Bybee *et al.* [32]; the time series of LAGs from each specimen is plotted as a separate curve. **B**, the data set *Allosaurus* fc1, in which age offsets, 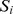, were applied to line up the time series by eye, as published in [32]. **C**, the data set *Allosaurus* fc2, in which a least-squares minimization procedure was applied to optimize the age offsets to produce a single cluster. **D**, splitting the *A. fragilis* femur data into two groups and separately clustering each group by using the least-squares method yields the *Allosaurus* fc3 (left cluster) and *Allosaurus* fc4 (right cluster) data sets.

The results for the femur vary substantially, however, depending on which method is used to assign age offsets (Fig. 7). The femur data for *Allosaurus* include six specimens. The alignment method used in [32] assigns an age of 14 years at time of death to two specimens whose femoral circumferences differ by a factor of 1.8 (190 mm vs. 338 mm, Fig. 7B). The corresponding mass difference is a factor of 4.8, if calculated by using the Anderson *et al.* bipedal formula [34] or a factor of 5.6 if calculated by isometric scaling. A roughly five-fold difference in mass between two individuals of the same age and same taxon seems biologically implausible, although not impossible.

The least-squares alignment method combines data from the six specimens into a single curve and yields a somewhat more plausible result: an estimated age of 11 years for the smaller femur and 14.5 years for the larger (Fig. 7C). The results of this method suggest, however, that the growth rate accelerated dramatically starting at about age 12, a pattern that seems biologically unlikely and is not consistent with trajectories seen in the humerus, tibia, or ulna data for *Allosaurus*.

Fig. 7D illustrates a third approach to assigning age offsets, which splits the femur data sets into two groups and to cluster each group separately by using the least-squares procedure. Splitting the data in this manner would be appropriate if the femora in the data set originated from two biologically distinct populations, such as different species, subspecies, or genders. Data sets fc3 and fc4 have been split and clustered separately.

**Figure 8:**
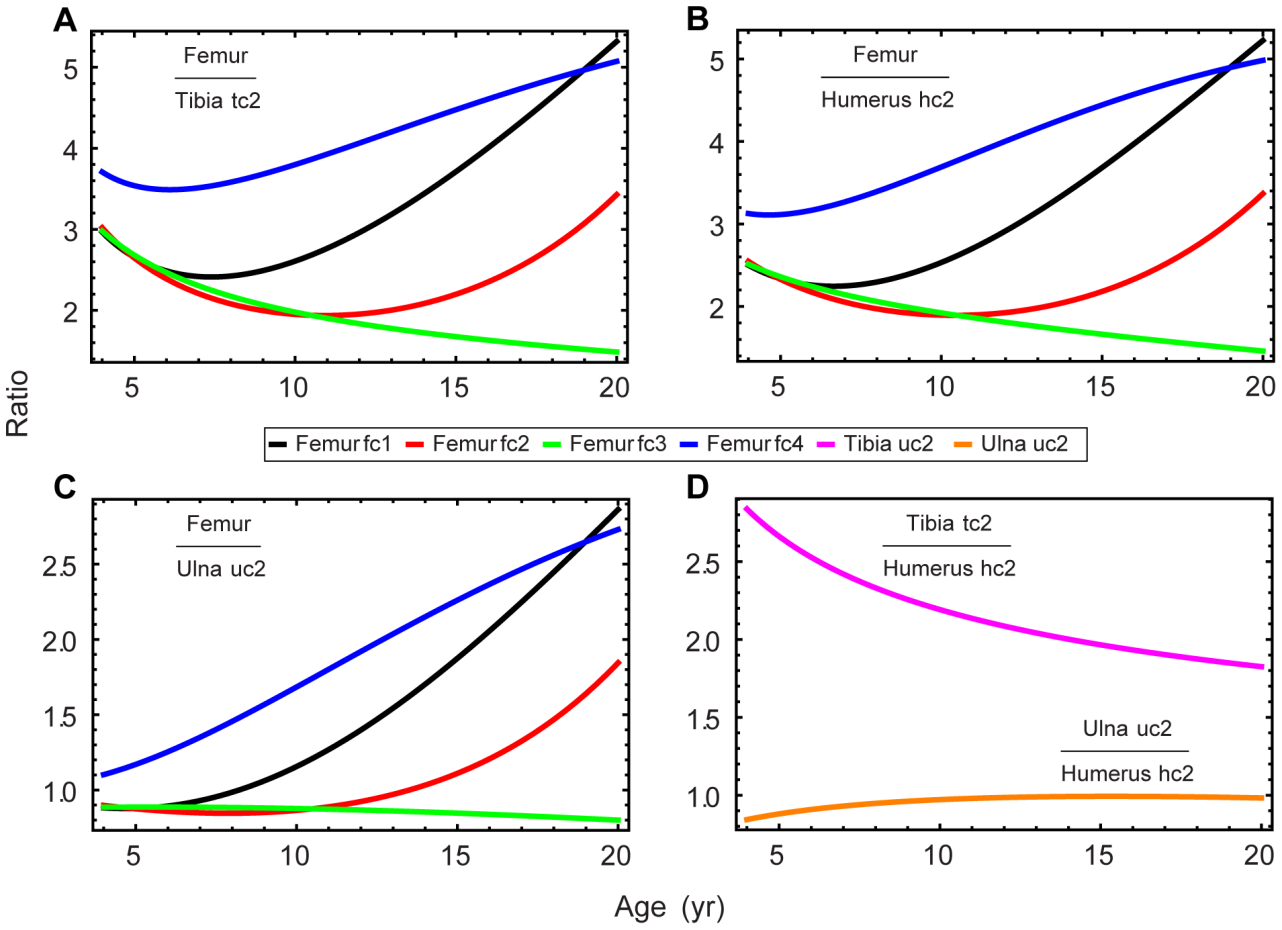
Ratios of *Allosaurus* long-bone growth models. The growth models that best fit longitudinal time series of LAG circumference are compared by plotting ratios of best-fit models for femur to the best-fit models for (**A**) the tc2 tibia, (**B**) hc2 humerus, and (**C**) uc2 ulna data sets. If *Allosaurus* grew isometrically, the ratios would be constant and thus would appear on the charts as horizontal lines. Four alternative time series, calculated using the models that best fit the femur data sets fc1, fc2, fc3 and fc4 (see Fig. 7), are plotted in each panel. Comparison of the ratio curves suggests that data set *Allosaurus* fc3 (green curve) is the best match to the humerus, tibia and ulna time series. The dramatic change with age observed in data set *Allosaurus* fc2 (red curve) suggests that the specimens it represents may be biologically different from those in the other data sets. **D**, the ratios of the best-fit models for the tibia tc2 data set to those for the humerus hc2 data set, and similarly the ratios for ulna uc2 to humerus hc2, both approach reasonable asymptotes, suggesting that those three data sets are compatible with one another.

Fig. 8 illustrates the relative growth rates of the bones by plotting the ratio of the models that best fit to them. Isometric growth implies that all bones grow at the same rate, which would appear as horizontal lines on these charts. Instead, we see evidence that growth was highly non-isometric, a finding that independently supports the primary conclusions of Bybee *et al.* [32] by the use of different models.

The ratio plots in Fig. 8 also test the consistency of the femur data sets with those for other bones. The non-isometric trajectories seen in Fig. 8A, with three of the four ratios increasing after an initial drop to value well above 1.0, implies that late in life the circumferences of the tibia and femur differ substantially. As a matter of biomechanics, such wide differences seems implausible because the strength of long bones under load is a function of circumference [99] and one expects strengths to be similar in different parts of the same leg.

The ratio of femur to tibia circumference for the data set *Allosaurus* fc3, in contrast, monotonically approaches an asymptote of 0.82 by age 20 (solid green curve in Fig. 8A). This is the trajectory that one would expect, and it suggests that the splitting and clustering of this femur data set is biologically valid and that *Allosaurus* fc3 is the femur data set that matches the tibia data set *Allosaurus* tc2. Similar patterns are seen in plots of the ratios of femur models to the humerus hc2 and ulna uc2 models (Figs. 8B and 8C). In each case, the ratio for the best-fit model to the fc3 data set approaches a plausible asymptote, whereas the other ratios rise at advanced ages to unreasonable levels. Both the ratio of tibia to humerus and that of ulna to humerus (Fig. 8D) approach reasonable asymptotes, which confirms that those data sets are compatible with one another.

Taken together, the asymptotic behavior of these ratios strongly suggests that the specimens in the *Allosaurus* fc3, hc2, tc2, and uc2 data sets all originated from the same taxon. Conversely, it also suggests that the three specimens in *Allosaurus* fc4 (UUVP 11164, UUVP 2656, and UUVP 3694) represent a biologically distinct group. The biological distinction could be a difference in taxon, sexual dimorphism, or perhaps an environmental factor, such as more favorable habitat. Another possible, but less likely, explanation is error in the data set, for example bone remodeling which altered the LAGs.

Only increasing models have strong statistical support when fit to the *Allosaurus* fc3 and fc4 data sets. We can therefore determine only the lower bound on the maximum size at skeletal maturity, which clearly must be larger than the largest specimens in the data set. Whereas the previous study [32] found a ratio of 1.8 between the femoral circumferences of two 14-year-old individuals, the split and clustered data sets fc3 and fc4 differ in femoral circumference by a factor of 2.1 at age 12, corresponding to a mass ratio of 7.6 (bipedal formula) or 9.3 (isometric scaling). Splitting the data sets reduces the precision of the method used to determine relative age offsets, so the actual size difference may be smaller than the mass ratios suggest.

Nevertheless, such a large difference in size suggests that the two groups may represent distinct taxa, especially considering the difficulty of making precise taxonomic assignments based only on isolated long bones. I conclude that, assuming the absence of systematic errors in the data, the most likely explanation is that UUVP 11164, UUVP 2656, and UUVP 3694 are from a fast-growing, and likely gigantic, unidentified Allosaurid taxon distinct from *A. fragilis*.

Although most taxonomic groups in paleontology are identified by osteology, taxonomy also recognizes formal groups identified from footprints (ichnotaxa), eggs (ootaxa), and other characteristics. The *Allosaurus* results presented here would, if confirmed, be the first of a new category, an anaptorythmic (from the ancient Greek for “growth rate”) taxon that is initially distinguished from other groups by its growth curve. Although the current finding must be regarded as tentative until confirmed by further research, this example shows some of the potential value in using growth curves to study dinosaurs.

## Conclusions

In order to obtain robust, reproducible results in model-based studies of dinosaur growth, it is essential that workers in this area address the conceptual and methodological issues identified here. Model selection and curve fitting should be performed in accordance with standard statistical methodologies and with care to recognize the assumptions and limitations of the models and their interpretation.

The analyses reported here find that only a few dinosaur growth data sets exhibit a marked slowing of growth with age and that most previous qualitative assumptions of asymptotic growth were incorrect. These findings have implications for the validity of other kinds of dinosaur studies. Estimates of peak dinosaur growth rates from Erickson *et al.* [20] have been incorporated, for example, in calculations of dinosaur body temperatures [109]. Those studies–as well as any others that make inferences from ages or maximum sizes derived from prior studies–should be reexamined because their inputs may not be valid.

One motivation for studying dinosaur growth rates is to compare them to extant animals such as crocodiles, birds and mammals. This approach has been used to infer aspects of dinosaur metabolism and other biological properties [5]–[15], [20], [22], [23], [25], [37]. The results here revise some growth rates upward and others downward. They do not change the broad conclusion that dinosaurs were relatively fast-growing creatures. The lack of data points and lack of mature specimens, however, implies that great caution should be used in drawing conclusions from the quantitative rates until more data has been analyzed.

A second surprising conclusion from this study is that so few skeletally mature specimens appear in the growth data sets. Mature individuals seem to be missing or underrepresented in the data on a wide range of taxonomic groups, including ornithopods, theropods, ceratopsians, hadrosaurs, sauropods and prosauropods.

This latter conclusion is consistent with the results of Horner and coworkers [110], who report that most of the dinosaur bone histology specimens they have examined are skeletally immature. It is also supported by the initial studies on dinosaur life tables that show steep mortality curves [17]–[19], [28]. A study by Sander and Tückmantel [111] of bone growth lamina in specimens of *Apatosaurus*, *Barosaurus*, *Brachiosaurus, Janenschia* and an unidentified diplodocid found consistent lamina thickness across the range of ages studied, which similarly implies a linear trend in bone growth and thus a lack of skeletal maturity.

The most parsimonious explanation for this pattern is that most dinosaur data sets to date are dominated by skeletally immature specimens, even at the largest sizes. It is possible, however, that the apparent bias is a result of random or systematic errors in the age estimation process–including, perhaps, the retrocalculation step in the whole-bone method. But, it is interesting that skeletally mature specimens are also absent in many longitudinal data sets that employ no retrocalculation.

One potential form of systematic error to consider is taxonomic misclassification. If mature individuals are assigned to a different taxon from juveniles, the result could appear as an absence of mature specimens. Although this might seem unlikely, Scannella and Horner [112] have argued, by analysis of an ontogenetic sequence, that *Triceratops* and *Torosaurus* should be considered a single taxon that includes both skeletally immature (*Triceratops*) and mature (*Torosaurus*) specimens. In their view, the specimens were misclassified as distinct taxa for many years because ontogenetic changes in cranial anatomy late in life altered the appearance of mature individuals so much that they initially seemed to warrant a separate genus. Erickson *et al.* [18] have similarly suggested that the *Psittacosaurus major* specimens should be united into a single taxon with *P. lujiatunensis*. Perhaps even larger psittacosaur taxa, representing the next stage of ontogeny, await discovery.

The new approach to estimation and analysis of growth rates presented here suggest several directions for future study. One high priority should be to improve the data sets available for growth analysis so that conclusions made from them carry a high degree of statistical confidence. A policy of transparency in publishing both complete data and a description of methodology detailed enough to enable independent replication of reported analyses is very important to the further development of dinosaur growth studies.

A straightforward approach would be to perform additional longitudinal studies on specimens that were previously examined by using the whole-bone method (see Table 1); for these specimens, much of the hard work of collection and preparation of histological slides has already been done. Measurements made from digital micrographs of the slides would enable both new analyses and verification of prior retrocalculation work.

I did not consider how the rate of tissue deposition may have influenced LAG formation, a topic that has been examined in histological studies [14], [111], [113]. Additional work could also improve the confidence with which we are able to estimate body masses from bone measurements, a task that is important but still involves a high degree of uncertainty. Characterizing DME and other mass estimation techniques by applying them to a range of extant animals would greatly improve our understanding of the potential sources of error in using this method to study extinct taxa.

Finally, the use of split clustering of longitudinal LAG data sets and of bone growth model ratios demonstrated here for *Allosaurus* holds promise as a possible way to detect misclassification of specimens, and perhaps even to detect previously unidentified taxa. More insight could be obtained from a comparison of bone ratios from articulated specimens, especially among specimens thought to be of similar age at time of death. In the intriguing case of *Allosaurus*, existing morphometric studies report bone length rather than circumference [114]–[116], so such a comparison must await further work.

## Supporting Information

Figure S1
**Plots of attempted replication of results from references** [**20]**, [24], [25] **.** The published regression equations from references [20], [24], [25] (red and brown) can be compared in these plots to the published data points (blue dots) and the best-fit curve for logistic function A (dark blue) and for function B (dashed magenta) and 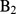 (green) or C (dashed magenta) and 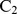 (light blue). Note that *P. mongoliensis* occurs in two different papers [20], [24] and thus has two regression equations. The fits for *Albertosaurus* and for *P. mongoliensis* are fairly close (see Table S1), but the other published curves are not very close to either the best-fit curves or the data points.(PDF)

Figure S2
**Plots of prior study best fits and attempted replication of results from reference** [**20**] **.** The published regression equations from reference [20] (red) are overlaid for comparison with the published data points (blue dots) and data points recovered from the curve via image processing (red dots). The best-fit curves for logistic function A to the published points (blue), 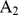 (dashed magenta) and to the recovered data A (green), 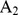 (dashed black) differ substantially from the curves corresponding to the published regression equations. **A**, **B**, data points for *Massospondylus* and *Syntarsus* data from references [5] and [6], as described in Text S1. **C**, **D**, adding the data points recovered from the plots yields different data sets (see discussion in Text S1).(PDF)

Figure S3 **Plots of prior study best fits and attempted replication of results from reference** [**37**] **.** The fits presented in reference [37] are shown in red, whereas the attempted replication best fits are drawn in black, as are the data points. Rather than best fits, the authors of [37] explicitly present two or three fit scenarios for each taxon in an effort to place lower and upper bounds on possible fits. In the case of *Apatosaurus* and *Alamosaurus*, at least some of the fit scenarios are close to being best fits. In the case of *Janenschia* and the Northampton sauropod, they are less successful.
(PDF)

Figure S4 **Detailed analysis of the** ***Massospondylus*** **plot from** [**20**] **.** **A**, a digital scan of the original plot from [20]. **B**, recovered data points have been overlaid in red. The close correspondence with the original data points shows that the overlaid plot is well registered with the scanned plot. **C**, the published data set, a curve fit to the published data set (blue) and a fit to the recovered points (green). Labeled features **a**, **b** and **c** are discussed in the Text S1. Each of the colored lines shows attempts to replicate the fit. The published regression equation (red curve) matches the curve in the original plot well by overlapping it throughout its range. None of the attempted replication fits, either to the full data set, or the recovered data set, match the curve in the original plot.
(PDF)

Figure S5 **Detailed analysis of the** ***Syntarsus*** **plot from** [**20**] **.** **A**, a digital scan of the original plot from [20]. **B**, the recovered data points overlaid in red. The close correspondence with the original data points shows that the overlap plot is reasonably well registered with the scanned plot. **C**, the regression equation (red), Chinsamy data set (blue dots), curves fit to the Chinsamy data set (blue, dashed magenta) and a fit to the recovered points (green, dashed black). Labeled features **a**, **b** and **c** are discussed in the Text S1. The published regression equation matches the curve in the original figure well, by overlapping it. So do the various attempted replication curves based on the recovered data set (green, dashed black). This strongly suggests that the regression equation was derived from the recovered data points (*i.e.*, the data points that appear in the figure), rather than from the full Chinsamy data set (blue dots). The attempted replication fits to the full Chinsamy data set (blue and dashed magenta) are substantially different than the regression equation.
(PDF)

Figure S6 **Detailed analysis of the** ***Tyrannosaurus*** **plot from** [**25**] **.** **A**, a digital scan of the original plot from [25]. **B**, the published data points overlaid in blue. The close correspondence with the original data points shows that the overlap plot is well registered with the scanned plot. One data point (labeled **a** in **B** and **C**) is not well registered. The plotted data point appears to correspond to a non-integer age (∼22.25 years), whereas all of the data points in the publish data set are LAG counts and thus are integers. **C**, the published age–mass data points overlaid in blue, along with the best-fit logistic function A (blue), best-fit logistic function B (dashed light blue) and the published regression equation (red). Labeled features **a**, **b** and **c** are discussed in the Text S1. The published regression equation (red) differs substantially from the plotted curve (see point **c**) in the plot. Neither the plotted curve nor the published regression equation matches any of the attempted replication fits.
(PDF)

Figure S7 **Detailed analysis of the** ***Psittacosaurus lujiatunensis*** **plot from** [**18**] **.** **A**, a digital scan of the original plot from Fig. 6 of [18]. **B**, the full data set overlaid in red points and the regression equation from the caption to Fig. 6 of [18]. Fits from logistic functions A, D, E, F and G are also overlaid on top of the plot. **C**, the original plot overlaid with the subset of data points that are histologically aged, along with fits to that data set. **D**, the data set modified to match the original plot data points, along with fits to that data set. Features **a**, **b**, **c**, **d** and **e** labeled with arrows are discussed in Text S1.
(PDF)

Figure S8 **Logistic curve used in the Monte Carlo examples.** **A**, regularly spaced sample points, and **B**, points displaced in time by a random amount drawn from a normal distribution to create a Monte Carlo sample.
(PDF)

Figure S9
**Estimates for maximum asymptotic growth parameter** 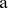 **from synthetic data.** Histograms of estimates for maximum asymptotic size 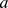 from 500 data sets generated by the Monte Carlo method with either homoscedastic or heteroscedastic error models as described in the text. In each case, the same curve sampled to generate the data (Logistic 3z of Fig. S8) was also used to do the fits; this is the original curve. The Monte Carlo data was then fit with either the same or a different asymptotic curve. Estimates of the parameter *a* made with age as the independent variable (blue histogram) have much larger standard deviation 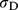, than the standard deviation 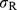 of those made with time as the independent variable (red histogram): 208% in the case of heteroscedastic errors and 409% in the case of homoscedastic errors when the original and fit curve were the same. The ratio of the standard deviation 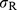 of the curve used to analyze the fits to the standard deviation 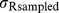 of the curve used to sample the data is 1 for the homoscedastic case because the analyzing and sampling curves are the same. In the other cases, however, the original curve and the one with which it is fit are different. In general, estimates of the parameter *a*, made by using age as the independent variable, have a much larger standard deviation, than do those that used time as the independent variable, although the ratio 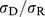 varies for the curve. The ratio of the standard deviation of each analyzing curve 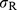 to the base case for the curve used to sample 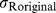 varies widely, from a low of 1% for Extreme Value 3a (i.e. a better estimate than using the same curve), to 623% for Morgan Mercer Flodin 4b. This shows that the choice of analysis curve used to fit can have a large impact on the quality of the resulting statistical estimates.(PDF)

Figure S10
**Finite samples of a sigmoidal curve can be best fit by increasing curves.** This figure summarizes the results of Monte Carlo experiments in which samples of 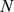 points for 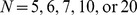 were chosen by a two-step method. The age was drawn from a normal distribution with mean 11.5 years and standard deviation 2.875 years to simulate the relative scarcity of very young and very old specimens. The age was used to sample the Logistic 3z curve of Fig S8. The ages may then have had error added (either homoscedastic or heteroscedastic error models) as in other Monte Carlo experiments (see Text S5) in this work. In a control group set, no error was added. The resulting data sets of 500 *N*-point samples were then fit with both increasing and asymptotic curves (see Text S5), and the fraction of best-fits that were increasing were tallied. Because the original sampled curve is sigmoidal, one would expect few if any of the zero-error data samples to be fit by an increasing curve, but that is not the case: for 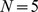, almost half of the N-point samples were best fit by increasing curves in the zero-error case. In all cases–but especially with zero error added–the tally of *N*-point samples best fit by increasing curves dropped with larger numbers of points per sample. I found that, with either heteroscedastic or homoscedastic errors added, the higher the error rate, the larger the fraction of best-fits that were increasing.(PDF)

Figure S11
**Scaling of Hutchinson** ***et al.*** **mass estimates vs. DME.** Mass estimates for four *T. rex* specimens reported by Hutchinson *et al.* [41] are plotted (black dots) against femur size on a log-log scale. The scenarios Min, Max, Ave, are the minimal, maximal and average estimates. The Hybrid scenario uses the Ave masses for all specimens except the largest one (“Sue”), for which the Min estimate is used. Mass estimates for each specimen obtained by using DME scaling are plotted in blue. DME has, by definition, a scaling exponent of mass with femur size of 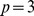. A one-parameter, DME-like scaling law has been fit (black line) to the black points, as has a two-parameter power law (red line).(PDF)

Figure S12 **Best-fit functions for each taxon.** Each chart plots the best-fit function obtained for the named data set from regression analysis, using size as the independent variable. The shaded area is the 95% confidence band, assuming normally distributed homoscedastic errors.
(PDF)

Table S1
**Functional form of growth functions** 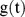 **.** This table includes both asymptotic functions, which approach a finite asymptote of 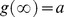, and increasing functions for which 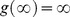. For each growth function, its inverse function  is also presented (if 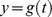, then 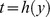), along with several properties of the function such as the starting value 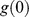, the growth rate 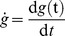, the age at the inflection point 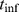, the size at the inflection point 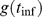, and the maximum growth rate 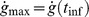 (which occurs at the inflection point). The inflection point and related functions are meaningful for sigmoidal curves, as they are not defined for increasing or attenuating curves. The constraints given define the parameter values for which the growth function are defined and real-valued. Most growth models in the literature employ three or four parameters. Sample size (*m*) is small for many of the dinosaur data sets, however, so it is important to include variations of the functions that include just two parameters. Although a two-parameter model is less flexible in fitting data patterns than is a model having more parameters, a two-parameter model can yield statistically valid results even when fit to as few as five data points. The corrected Akaike information criterion (AIC*_c_*) is infinite whenever the number of parameters is not less than 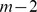. N.M.: not meaningful.(DOCX)

Table S2
**Best fits and strongly supported fits.** The 62 asymptotic models and 15 increasing models used in this study are listed. For each model, counts are given of the number of age–bone dimension data sets for which the model provided the best fit (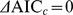) or strong support (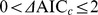) when fit. Counts are shown separately for direct fits (which used age as the independent variable) and reverse fits (which used age as the dependent variable). Of the functions shown, 41 had strong statistical support when fit to at least one data set.(XLSX)

Table S3
**Fits to line and cubic data.** For each of the 62 asymptotic models used in this study, the maximum asymptotic parameter 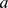 and the correlation coefficient 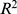 are given for fits to the same synthetically generated linear and cubic data sets plotted in Fig. 3. The high values of 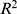 indicate that all of these sigmoid models can achieve an excellent fit to linear data, and most to cubic data as well. Attenuating curves are more variable, but the majority of these also achieve excellent fits. The wide range of values obtained for the maximum asymptotic size 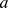, which varies from 2.89 to 8.46×10^55^ for these fits to the same linear data (and a similarly wide range for fits to the cubic data) demonstrates that these parameters are mathematical artifacts that have no predictive value, although the fits will work properly in the vicinity of the data points.(XLSX)

Table S4
**Detailed results of attempts to replicate previously published findings. A.** For each data set examined, values of three biological parameters–maximum asymptotic size 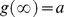, maximum growth rate 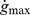, and age at maximum rate of growth (also called inflection point) 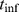
–are given for both the regression equations published in previous studies and for the best fits found to the data sets (see Text S1). In cases where the reported value is zero, N.A. appears for the ratio. **B.** Model parameters 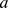, 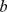, 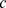 and 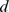 for previously reported regression equations and for the best-fit models from this study. Table entries are blank where the model does not include the parameter. The parameters given depend on the model. Numbers listed in bold are fixed in order to investigate previously reported fits. For any A2, C2 D2, E2, F2 or G2 fit, the value of *a* is fixed up front and not part of the fit. For any B2 fit, the values of both *a* and *d* are fixed up front and are not part of the fit.(XLSX)

Table S5
**Summary of replication results for 11 taxa from five dinosaur growth studies.** For each data set, values of maximum asymptotic size 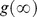, age at maximum rate of growth (also called inflection point) 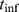, and maximum growth rate, 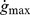, expressed in absolute terms and as percentage of mass added per year are given for both the regression equation published in previous studies and for the best fits of replication attempts.(XLSX)

Table S6 **Data sets of dinosaur growth gathered from the literature and used in this study.** The size parameter is a linear dimension (*e.g.*, femur length), as described in Table 2. *Tyrannosaurus* 2 comprises the *Tyrannosaurus* 1 data set supplemented with a specimen from [40] and one from [41]. All other data sets are taken from a single original source (see Table 2 for references). Age is reported as estimated age in years from the original source. The data sets for *Allosaurus* include several variations, as discussed in the paper. The *Psittacosaurus l*1 data set contains all 80 data points from the source article. One fossil specimen block in this data set includes 34 individuals, however, only one of which was measured; the data points are thus not independent measurements. Several other data points in that data set similarly represent a single measurement duplicated to cover multiple individuals in a find. To avoid overweighting the fits to these duplicate data points (for which the age is only imprecisely known), I prepared the *Psittacosaurus l*2 data set, which contains each point listed only once (a total of 39 data points). *Psittacosaurus l*3 is the subset of the data for which age was determined histologically, according to the original reference; *Psittacosaurus l*4 is the same set but with duplicate data points removed.
(XLSX)

Table S7 **Age–mass data used to attempt replication of prior results.** The data was gathered from references (see Table 2). In several cases, the scaling mass used for DME had to be estimated from digital scans of published plots–see Text S1 for details. The *Psittacosaurus l*5 data set is the recovered data set (see Text S1 for details), made to conform to what appears to be plotted in Fig. 6 of reference [18], and *Psittacosaurus l*6 is the same data set but with only one point for each distinct age-mass pair.
(XLSX)

Table S8
**Parameters for best-fit models.** Growth models and their associated parameters for each taxon are shown, along with the 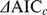 values for each model. All models having strong statistical support (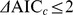) are shown; the best-fitting model has 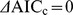. In addition, the best-fitting sigmoid and attenuating curves (*i.e.*, those having the lowest 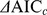) are shown for each taxon even if they were not among the models garnering strong support.(XLSX)

Table S9 **Scaling of DME vs. Hutchinson** ***et al.*** **for five specimens of** ***Tyrannosaurus rex*** **.** Femur length and mass estimates for minimal (Min), maximal (Max), average (Ave) and Hybrid scenarios, as calculated by Hutchinson *et al.* [41], are presented, along with the mass estimate derived by DME and the ratio between the DME estimate and the Hutchinson mass estimate. By construction, the DME mass is equal to the Hutchinson *et al.* mass for the largest specimen (“Sue”), so the ratios in that case are precisely 1.0.
(XLSX)

Table S10 **Least squares verification spreadsheet.** The data from Table S8 is presented along with the original regression equations from [20], [24], [25], and the attempted replication regression equations. For each regression equation, the sum of square of errors is calculated to show that the attempted replication fits are better fits (smaller sum of square errors) than the original fits. See Text S1 for details.
(XLSX)

Text S1 **Attempting to replicate prior results.**
(DOCX)

Text S2 **Impact of the choice of independent variable.**
(DOCX)

Text S3 **Impact of the choice of sigmoid curve.**
(DOCX)

Text S4 ***R*** **^2^ is not an adequate goodness-of-fit measure.**
(DOCX)

Text S5 **Impact of finite sample size.**
(DOCX)

Text S6 **Hutchinson** ***et al.*** **2011 and DME.**
(DOCX)

Text S7 **Discussion of growth models and fitting.**
(DOCX)
