# Supplementary material for: Revisiting the Estimation of Dinosaur Growth Rates
Source: PLoS One. 2013 Dec 16;8(12):e81917. doi: 10.1371/journal.pone.0081917 (PMC3864909; doi:10.1371/journal.pone.0081917)
Supplement: Figure S1 — Plots of attempted replication of results from references [20] , [24], [25] . The published regression equations from references [20], [24], [25] (red and brown) can be compared in these plots to the published data points (blue dots) and the best-fit curve for logistic function A (dark blue) and for function B (dashed magenta) and (green) or C (dashed magenta) and (light blue). Note that P. mongoliensis occurs in two different papers [20], [24] and thus has two regression equations. The fits for Albertosaurus and for P. mongoliensis are fairly close (see Table S1), but the other published curves are not very close to either the best-fit curves or the data points. (PDF) [file pone.0081917.s001.pdf]

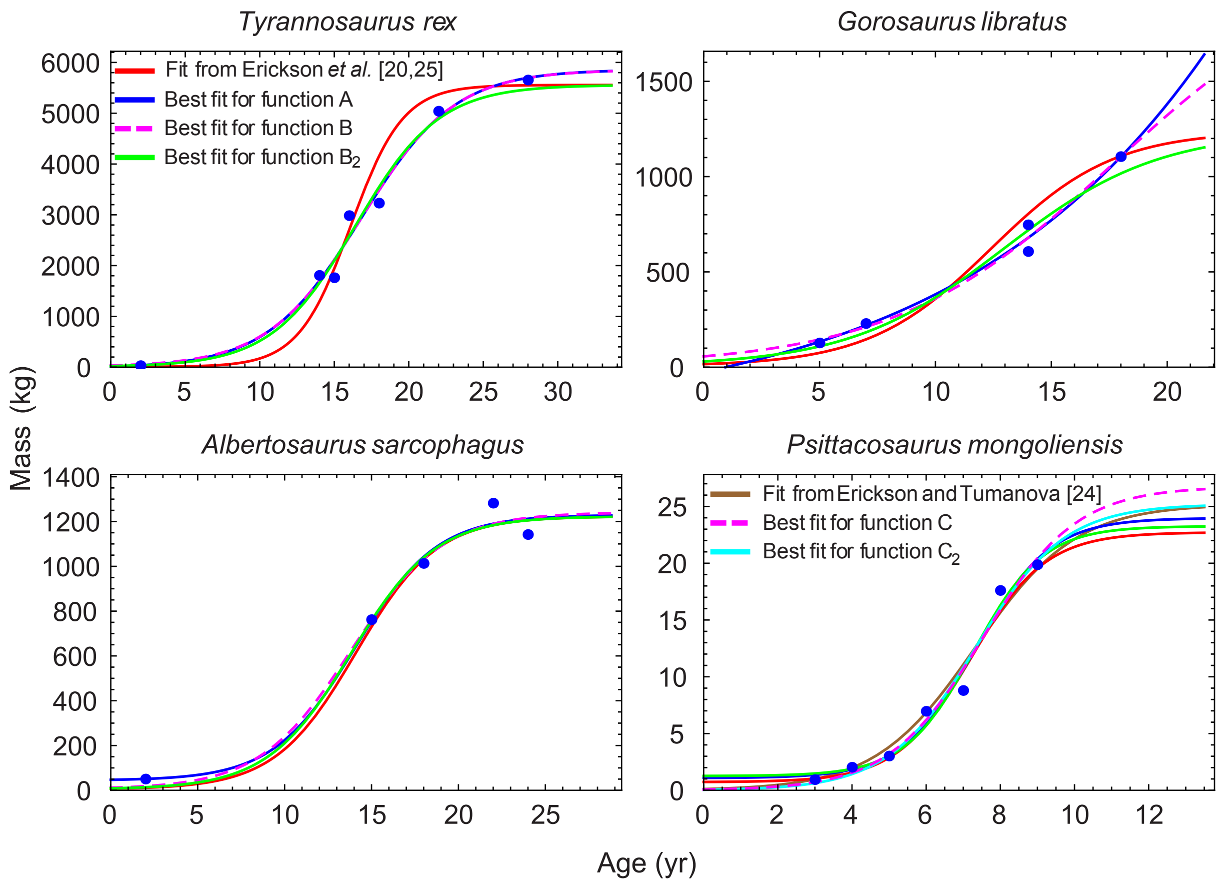

Tyrannosaurus rex
Gorosaurus libratus
Albertosaurus sarcophagus
Psittacosaurus mongoliensis
Fit from Erickson et al. [20,25]
Best fit for function A
Best fit for function B
Best fit for function B2
Fit from Erickson and Tumanova [24]
Best fit for function C
Best fit for function C2
Mass (kg)
Age (yr)
