# Supplementary figures and images for: Revisiting the Estimation of Dinosaur Growth Rates
Source: PLoS One. 2013 Dec 16;8(12):e81917. doi: 10.1371/journal.pone.0081917 (PMC3864909; doi:10.1371/journal.pone.0081917)

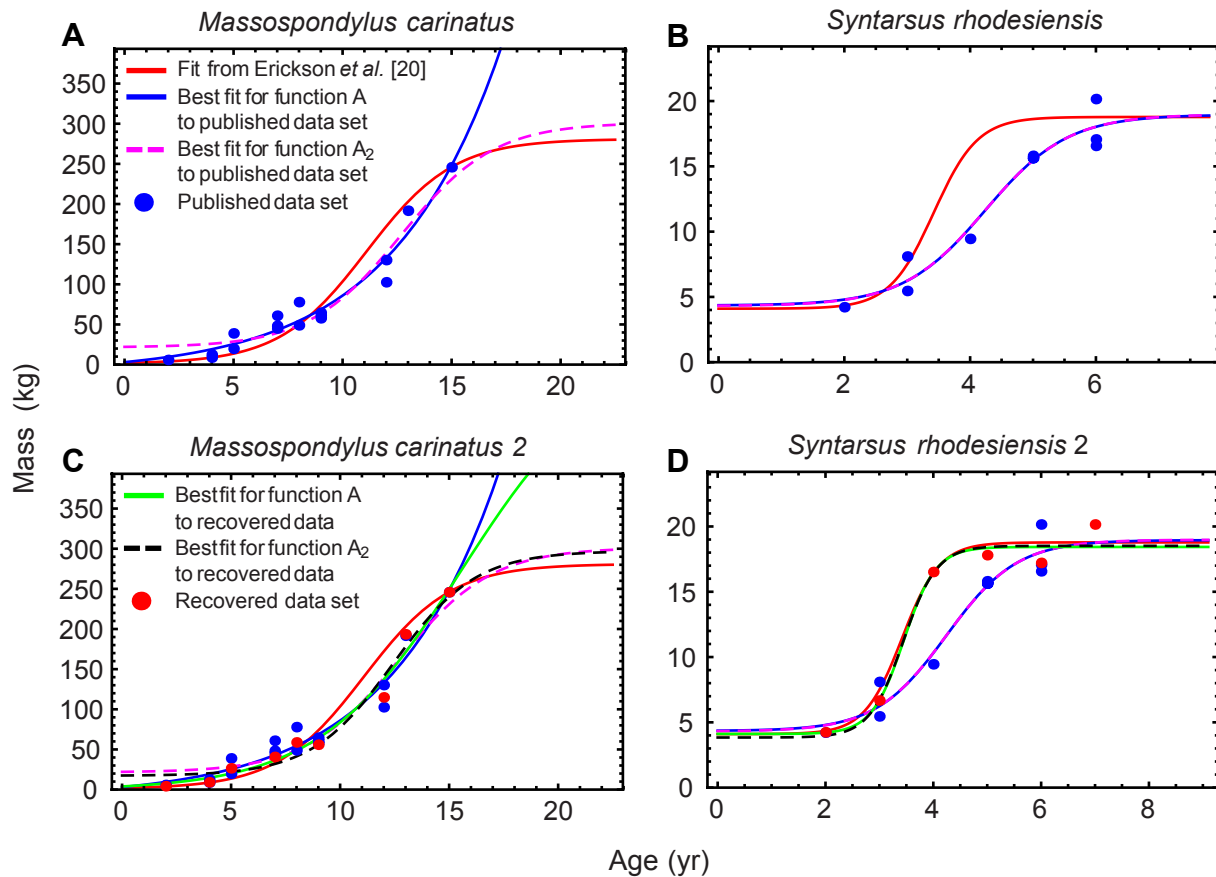

Supplement: Figure S2 — Plots of prior study best fits and attempted replication of results from reference [20] . The published regression equations from reference [20] (red) are overlaid for comparison with the published data points (blue dots) and data points recovered from the curve via image processing (red dots). The best-fit curves for logistic function A to the published points (blue), (dashed magenta) and to the recovered data A (green), (dashed black) differ substantially from the curves corresponding to the published regression equations. A, B, data points for Massospondylus and Syntarsus data from references [5] and [6], as described in Text S1. C, D, adding the data points recovered from the plots yields different data sets (see discussion in Text S1). (PDF) [file pone.0081917.s002.pdf]

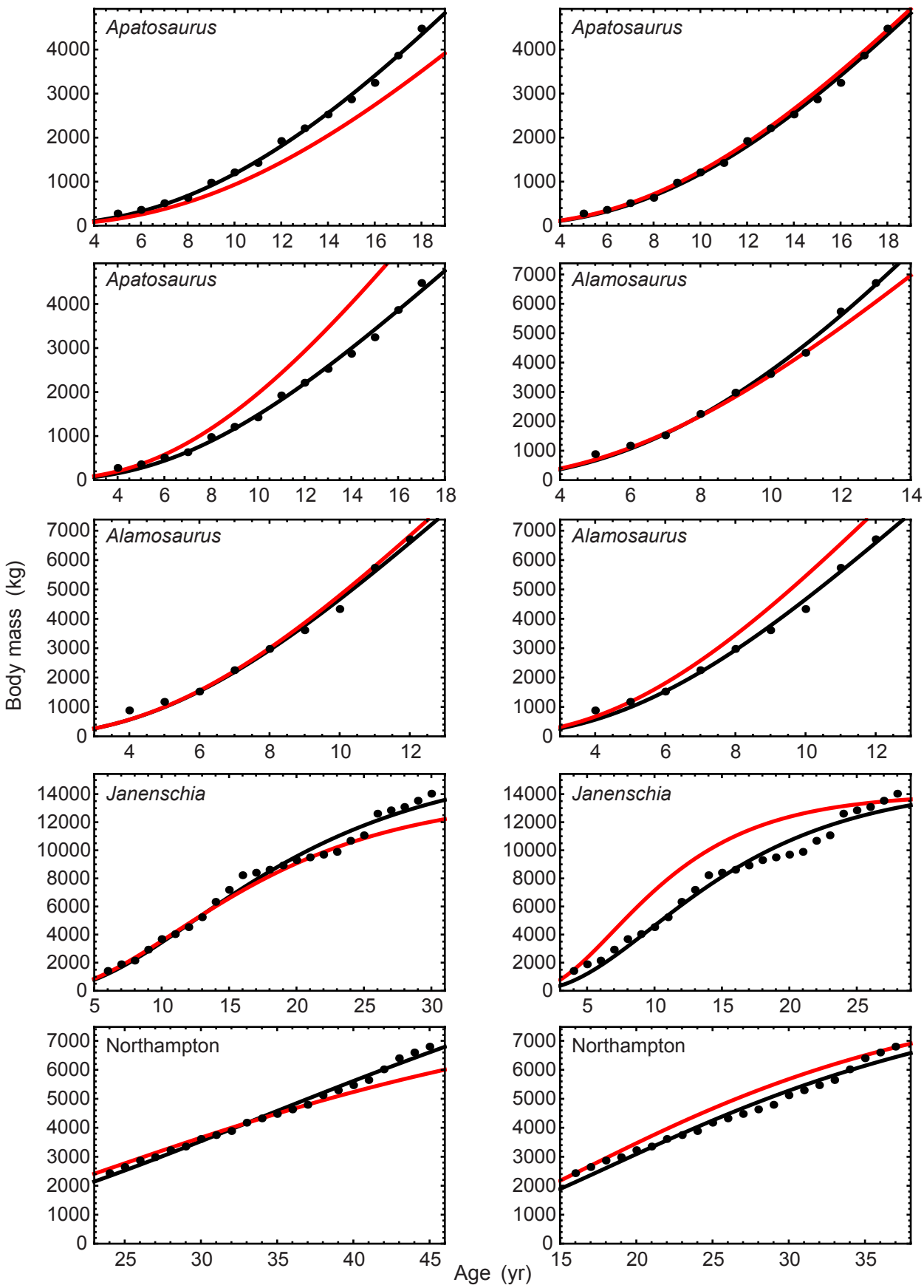

Supplement: Figure S3 — Plots of prior study best fits and attempted replication of results from reference [37] . The fits presented in reference [37] are shown in red, whereas the attempted replication best fits are drawn in black, as are the data points. Rather than best fits, the authors of [37] explicitly present two or three fit scenarios for each taxon in an effort to place lower and upper bounds on possible fits. In the case of Apatosaurus and Alamosaurus, at least some of the fit scenarios are close to being best fits. In the case of Janenschia and the Northampton sauropod, they are less successful. (PDF) [file pone.0081917.s003.pdf]

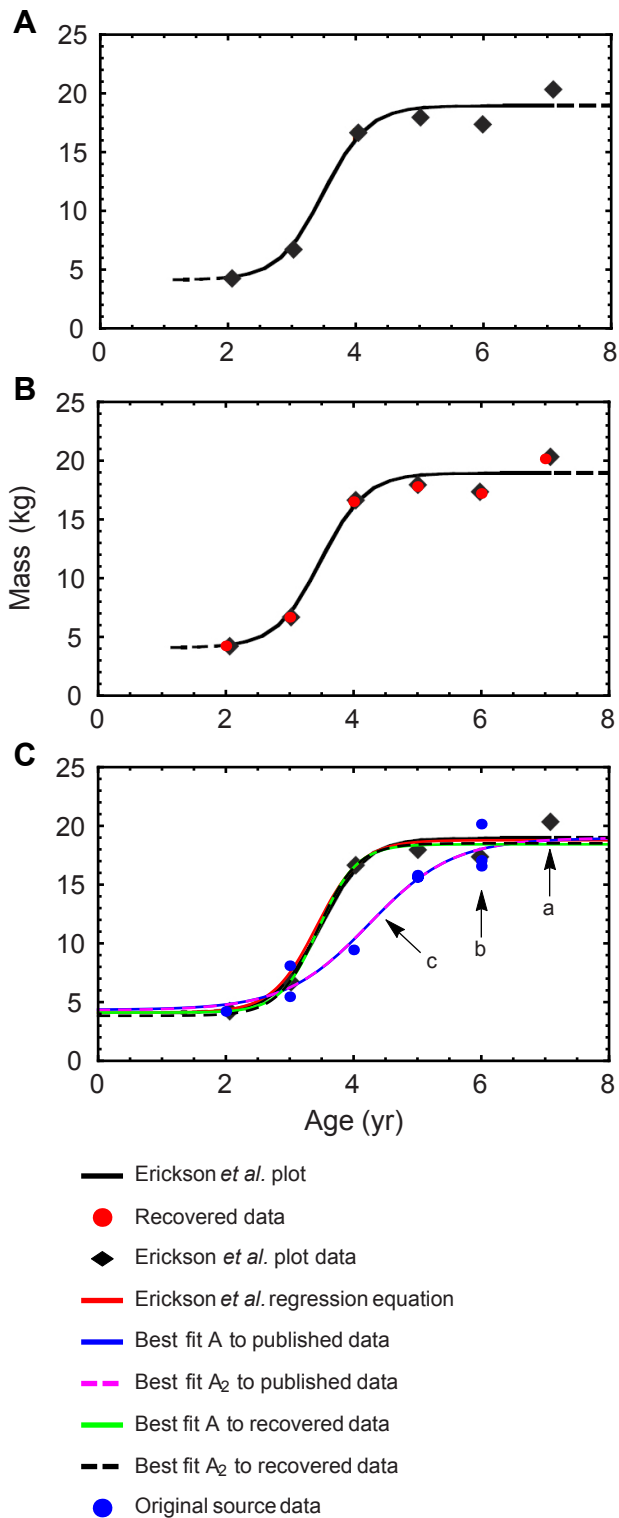

Supplement: Figure S5 — Detailed analysis of the Syntarsus plot from [20] . A, a digital scan of the original plot from [20]. B, the recovered data points overlaid in red. The close correspondence with the original data points shows that the overlap plot is reasonably well registered with the scanned plot. C, the regression equation (red), Chinsamy data set (blue dots), curves fit to the Chinsamy data set (blue, dashed magenta) and a fit to the recovered points (green, dashed black). Labeled features a, b and c are discussed in the Text S1. The published regression equation matches the curve in the original figure well, by overlapping it. So do the various attempted replication curves based on the recovered data set (green, dashed black). This strongly suggests that the regression equation was derived from the recovered data points (i.e., the data points that appear in the figure), rather than from the full Chinsamy data set (blue dots). The attempted replication fits to the full Chinsamy data set (blue and dashed magenta) are substantially different than the regression equation. (PDF) [file pone.0081917.s005.pdf]

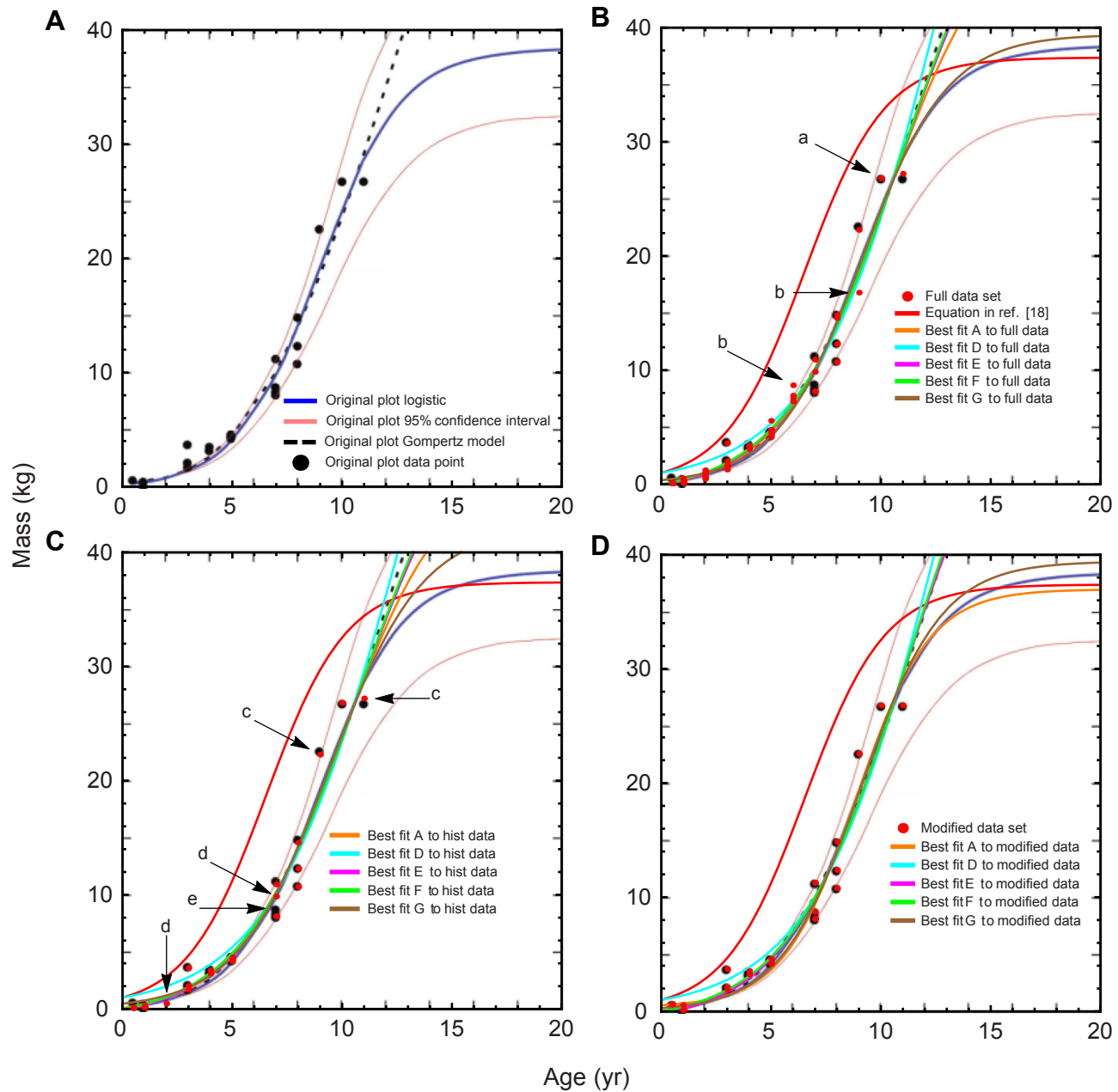

Supplement: Figure S7 — Detailed analysis of the Psittacosaurus lujiatunensis plot from [18] . A, a digital scan of the original plot from Fig. 6 of [18]. B, the full data set overlaid in red points and the regression equation from the caption to Fig. 6 of [18]. Fits from logistic functions A, D, E, F and G are also overlaid on top of the plot. C, the original plot overlaid with the subset of data points that are histologically aged, along with fits to that data set. D, the data set modified to match the original plot data points, along with fits to that data set. Features a, b, c, d and e labeled with arrows are discussed in Text S1. (PDF) [file pone.0081917.s007.pdf]

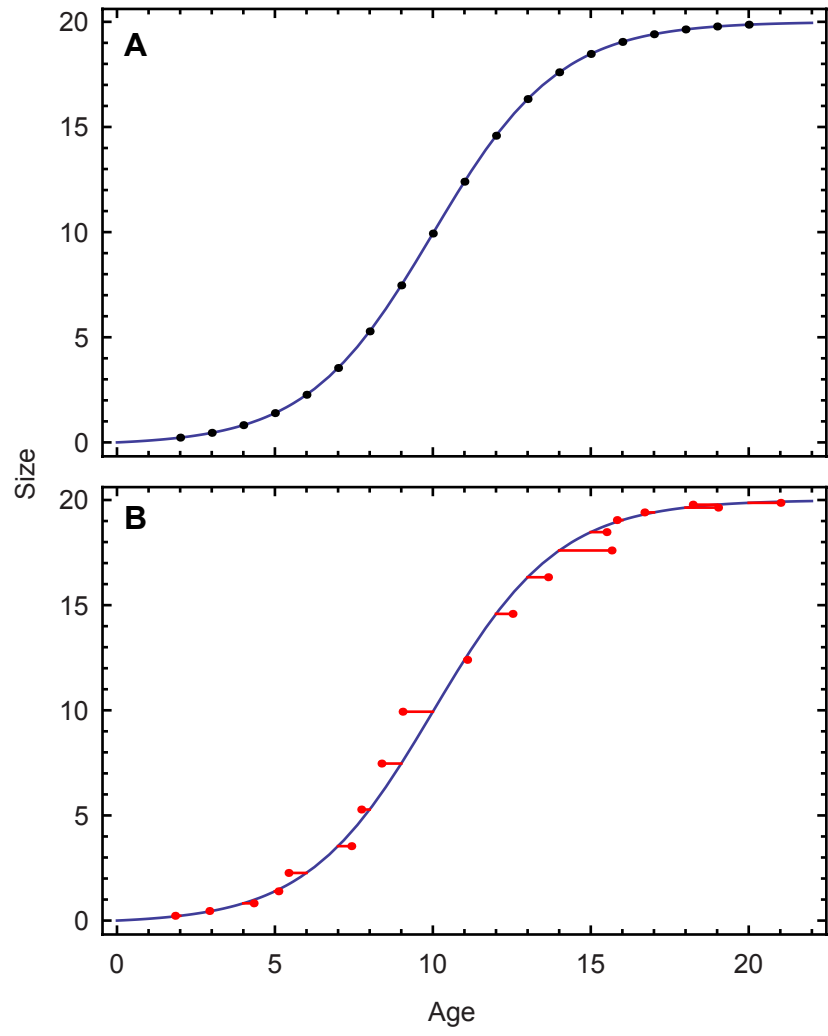

Supplement: Figure S8 — Logistic curve used in the Monte Carlo examples. A, regularly spaced sample points, and B, points displaced in time by a random amount drawn from a normal distribution to create a Monte Carlo sample. (PDF) [file pone.0081917.s008.pdf]

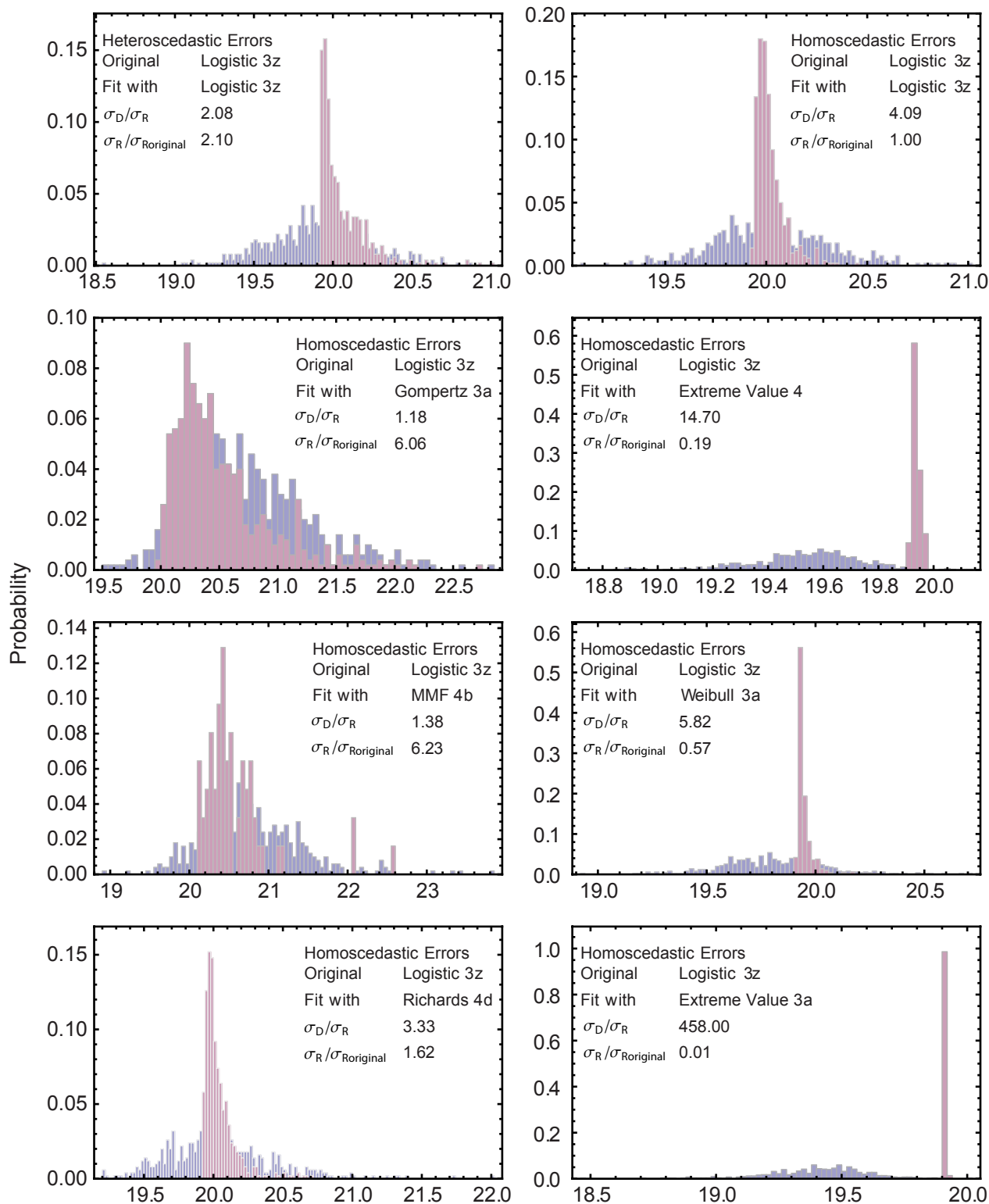

Supplement: Figure S9 — Estimates for maximum asymptotic growth parameter from synthetic data. Histograms of estimates for maximum asymptotic size from 500 data sets generated by the Monte Carlo method with either homoscedastic or heteroscedastic error models as described in the text. In each case, the same curve sampled to generate the data (Logistic 3z of Fig. S8) was also used to do the fits; this is the original curve. The Monte Carlo data was then fit with either the same or a different asymptotic curve. Estimates of the parameter a made with age as the independent variable (blue histogram) have much larger standard deviation , than the standard deviation of those made with time as the independent variable (red histogram): 208% in the case of heteroscedastic errors and 409% in the case of homoscedastic errors when the original and fit curve were the same. The ratio of the standard deviation of the curve used to analyze the fits to the standard deviation of the curve used to sample the data is 1 for the homoscedastic case because the analyzing and sampling curves are the same. In the other cases, however, the original curve and the one with which it is fit are different. In general, estimates of the parameter a, made by using age as the independent variable, have a much larger standard deviation, than do those that used time as the independent variable, although the ratio varies for the curve. The ratio of the standard deviation of each analyzing curve to the base case for the curve used to sample varies widely, from a low of 1% for Extreme Value 3a (i.e. a better estimate than using the same curve), to 623% for Morgan Mercer Flodin 4b. This shows that the choice of analysis curve used to fit can have a large impact on the quality of the resulting statistical estimates. (PDF) [file pone.0081917.s009.pdf]

Heteroscedastic Error Model

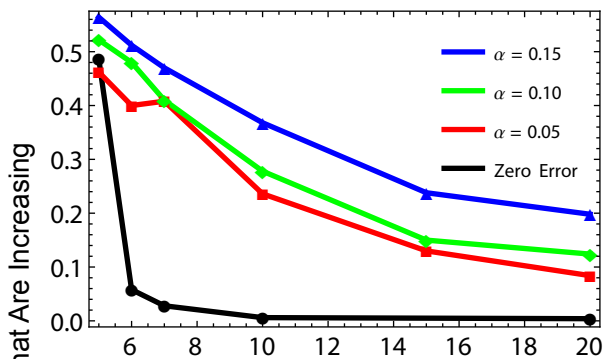

Homoscedastic Error Model

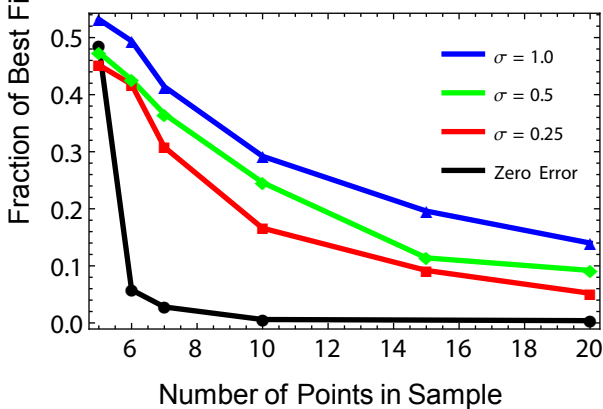

Supplement: Figure S10 — Finite samples of a sigmoidal curve can be best fit by increasing curves. This figure summarizes the results of Monte Carlo experiments in which samples of points for were chosen by a two-step method. The age was drawn from a normal distribution with mean 11.5 years and standard deviation 2.875 years to simulate the relative scarcity of very young and very old specimens. The age was used to sample the Logistic 3z curve of Fig S8. The ages may then have had error added (either homoscedastic or heteroscedastic error models) as in other Monte Carlo experiments (see Text S5) in this work. In a control group set, no error was added. The resulting data sets of 500 N-point samples were then fit with both increasing and asymptotic curves (see Text S5), and the fraction of best-fits that were increasing were tallied. Because the original sampled curve is sigmoidal, one would expect few if any of the zero-error data samples to be fit by an increasing curve, but that is not the case: for , almost half of the N-point samples were best fit by increasing curves in the zero-error case. In all cases–but especially with zero error added–the tally of N-point samples best fit by increasing curves dropped with larger numbers of points per sample. I found that, with either heteroscedastic or homoscedastic errors added, the higher the error rate, the larger the fraction of best-fits that were increasing. (PDF) [file pone.0081917.s010.pdf]

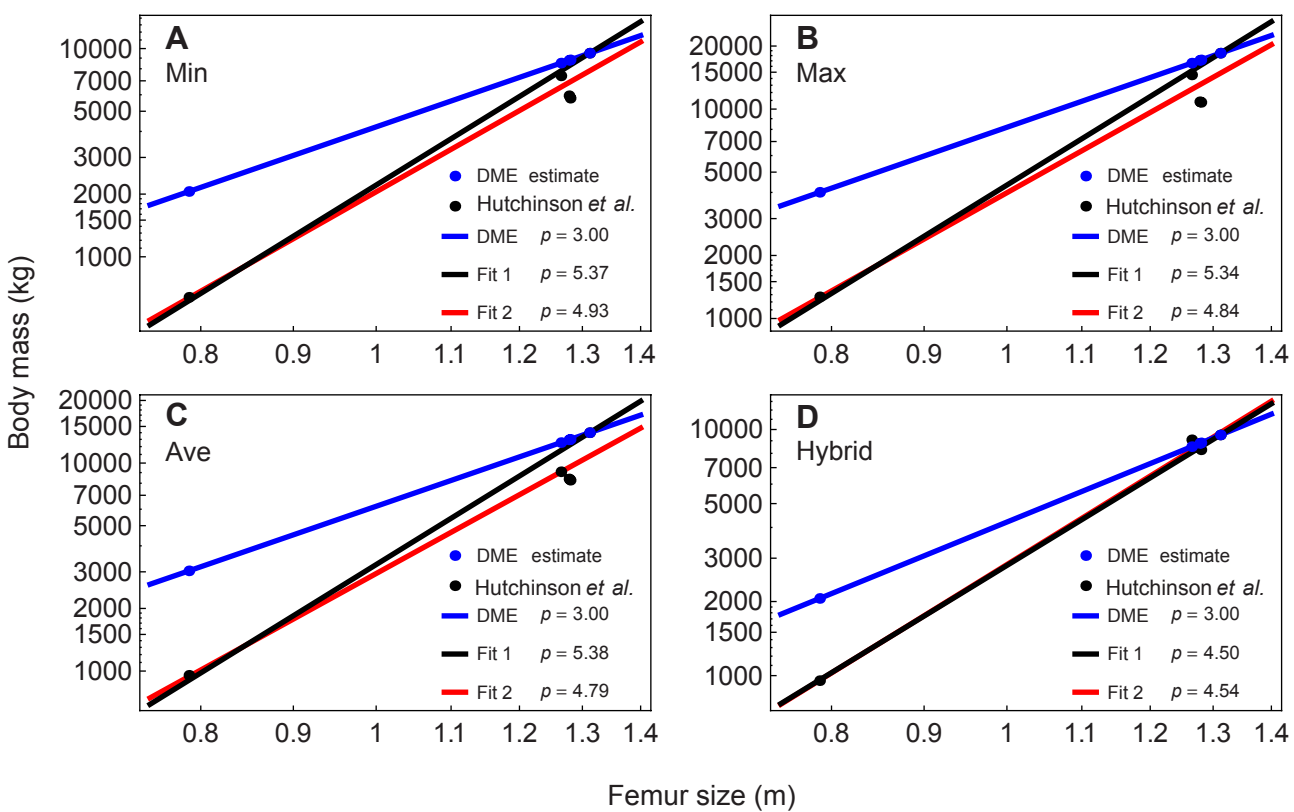

Supplement: Figure S11 — Scaling of Hutchinson et al. mass estimates vs. DME. Mass estimates for four T. rex specimens reported by Hutchinson et al. [41] are plotted (black dots) against femur size on a log-log scale. The scenarios Min, Max, Ave, are the minimal, maximal and average estimates. The Hybrid scenario uses the Ave masses for all specimens except the largest one (“Sue”), for which the Min estimate is used. Mass estimates for each specimen obtained by using DME scaling are plotted in blue. DME has, by definition, a scaling exponent of mass with femur size of . A one-parameter, DME-like scaling law has been fit (black line) to the black points, as has a two-parameter power law (red line). (PDF) [file pone.0081917.s011.pdf]
