# Supplementary material for: Revisiting the Estimation of Dinosaur Growth Rates
Source: PLoS One. 2013 Dec 16;8(12):e81917. doi: 10.1371/journal.pone.0081917 (PMC3864909; doi:10.1371/journal.pone.0081917)
Supplement: Figure S4 — Detailed analysis of the Massospondylus plot from [20] . A, a digital scan of the original plot from [20]. B, recovered data points have been overlaid in red. The close correspondence with the original data points shows that the overlaid plot is well registered with the scanned plot. C, the published data set, a curve fit to the published data set (blue) and a fit to the recovered points (green). Labeled features a, b and c are discussed in the Text S1. Each of the colored lines shows attempts to replicate the fit. The published regression equation (red curve) matches the curve in the original plot well by overlapping it throughout its range. None of the attempted replication fits, either to the full data set, or the recovered data set, match the curve in the original plot. (PDF) [file pone.0081917.s004.pdf]

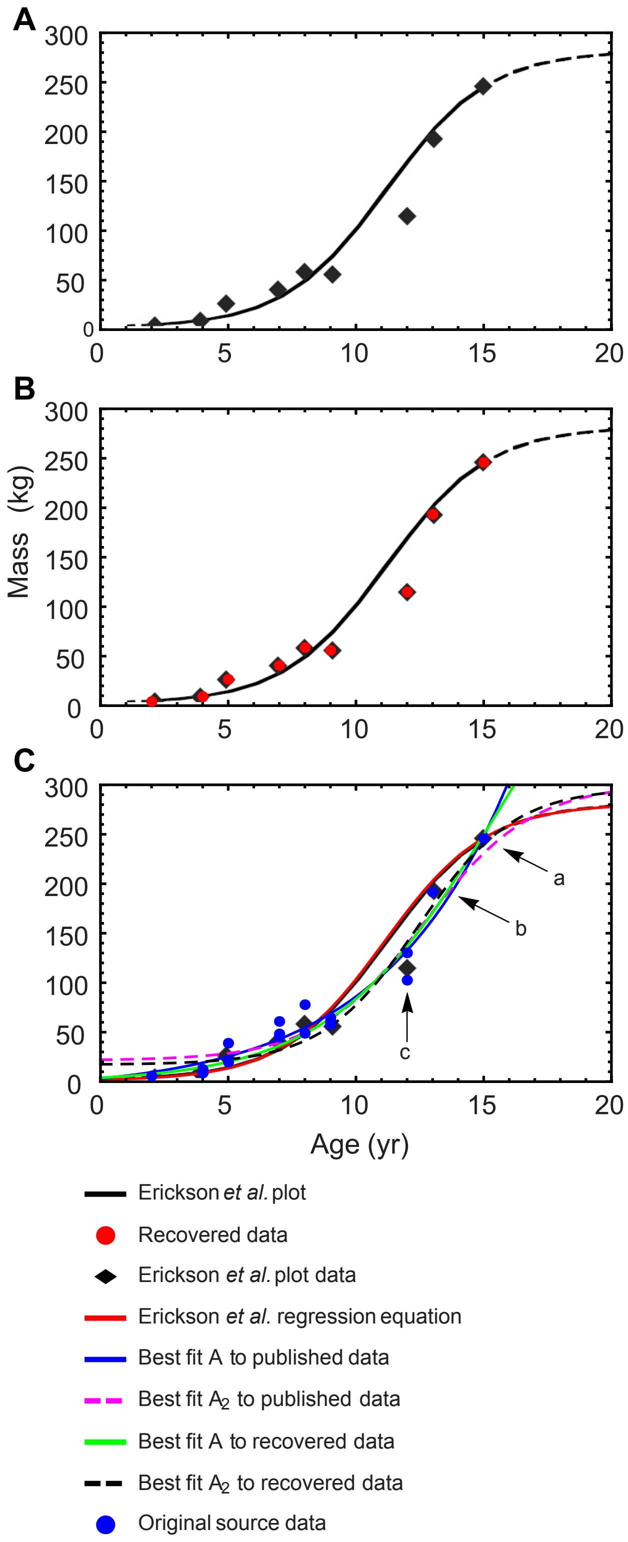

A
B
C
Mass (kg)
Age (yr)
a
b
c
Erickson et al. plot
Recovered data
Erickson et al. plot data
Erickson et al. regression equation
Best fit A to published data
Best fit A2 to published data
Best fit A to recovered data
Best fit A2 to recovered data
Original source data
