# Supplementary material for: Revisiting the Estimation of Dinosaur Growth Rates
Source: PLoS One. 2013 Dec 16;8(12):e81917. doi: 10.1371/journal.pone.0081917 (PMC3864909; doi:10.1371/journal.pone.0081917)
Supplement: Figure S6 — Detailed analysis of the Tyrannosaurus plot from [25] . A, a digital scan of the original plot from [25]. B, the published data points overlaid in blue. The close correspondence with the original data points shows that the overlap plot is well registered with the scanned plot. One data point (labeled a in B and C) is not well registered. The plotted data point appears to correspond to a non-integer age (∼22.25 years), whereas all of the data points in the publish data set are LAG counts and thus are integers. C, the published age–mass data points overlaid in blue, along with the best-fit logistic function A (blue), best-fit logistic function B (dashed light blue) and the published regression equation (red). Labeled features a, b and c are discussed in the Text S1. The published regression equation (red) differs substantially from the plotted curve (see point c) in the plot. Neither the plotted curve nor the published regression equation matches any of the attempted replication fits. (PDF) [file pone.0081917.s006.pdf]

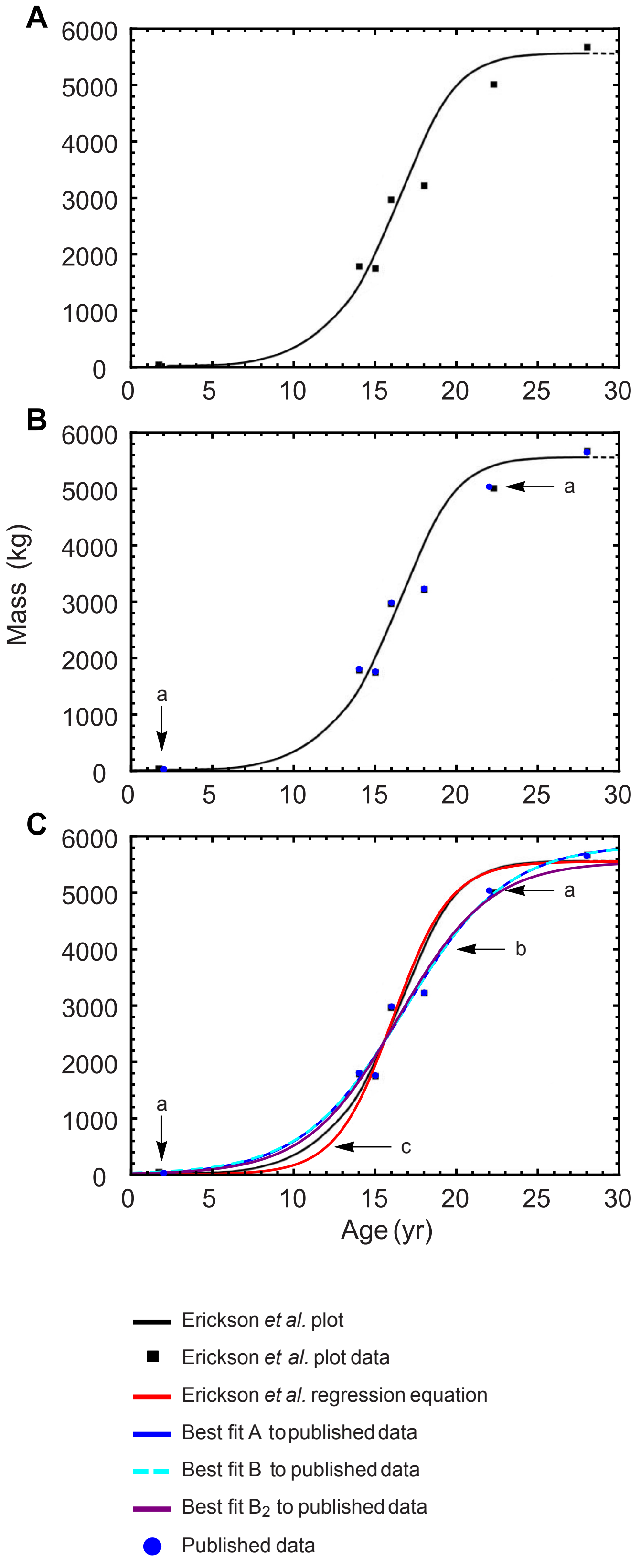
A
B
C
Mass (kg)
Age (yr)
0
1000
2000
3000
4000
5000
6000
0
5
10
15
20
25
30
a
b
c
Erickson et al. plot
Erickson et al. plot data
Erickson et al. regression equation
Best fit A to published data
Best fit B to published data
Best fit B2 to published data
Published data
