# Supplementary material for: Revisiting the Estimation of Dinosaur Growth Rates
Source: PLoS One. 2013 Dec 16;8(12):e81917. doi: 10.1371/journal.pone.0081917 (PMC3864909; doi:10.1371/journal.pone.0081917)
Supplement: Figure S12 — Best-fit functions for each taxon. Each chart plots the best-fit function obtained for the named data set from regression analysis, using size as the independent variable. The shaded area is the 95% confidence band, assuming normally distributed homoscedastic errors. (PDF) [file pone.0081917.s012.pdf]

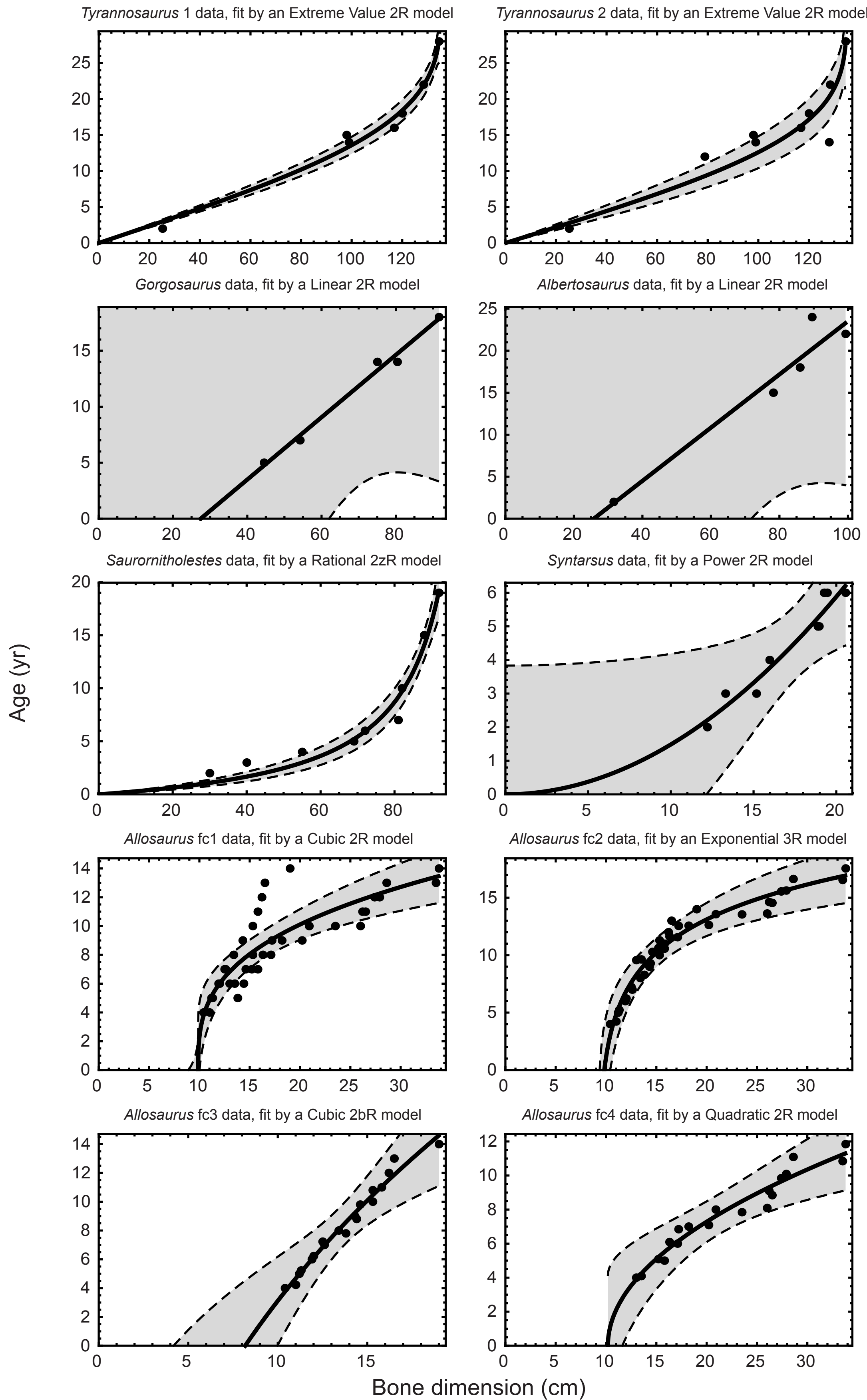

Tyrannosaurus 1 data, fit by an Extreme Value 2R model
Tyrannosaurus 2 data, fit by an Extreme Value 2R model
Gorgosaurus data, fit by a Linear 2R model
Albertosaurus data, fit by a Linear 2R model
Saurornitholestes data, fit by a Rational 2zR model
Syntarsus data, fit by a Power 2R model
Allosaurus fc1 data, fit by a Cubic 2R model
Allosaurus fc2 data, fit by an Exponential 3R model
Allosaurus fc3 data, fit by a Cubic 2bR model
Allosaurus fc4 data, fit by a Quadratic 2R model
Age (yr)
Bone dimension (cm)

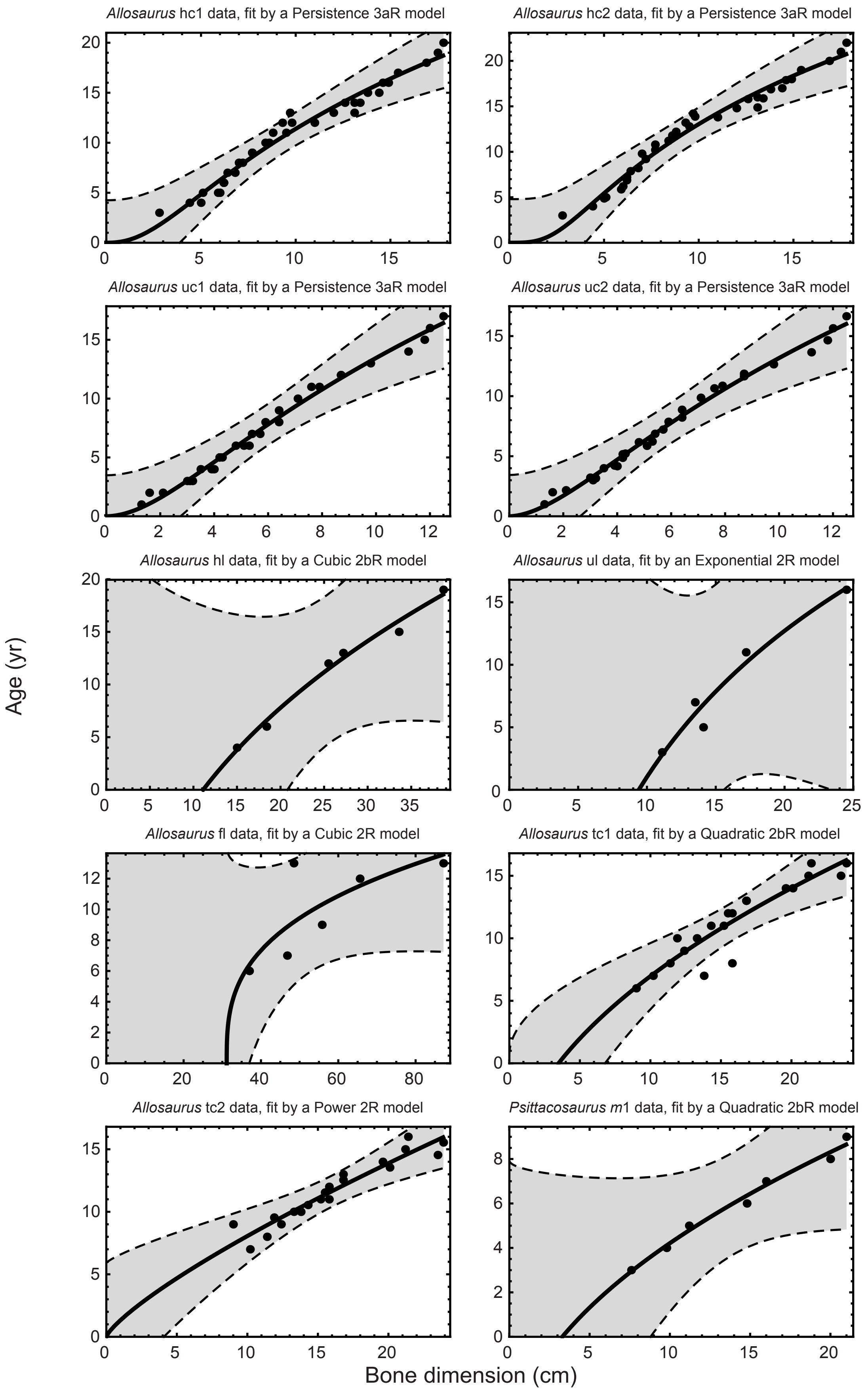
Allosaurus hc1 data, fit by a Persistence 3aR model
Allosaurus hc2 data, fit by a Persistence 3aR model
Allosaurus uc1 data, fit by a Persistence 3aR model
Allosaurus uc2 data, fit by a Persistence 3aR model
Allosaurus hl data, fit by a Cubic 2bR model
Allosaurus ul data, fit by an Exponential 2R model
Allosaurus fl data, fit by a Cubic 2R model
Allosaurus tc1 data, fit by a Quadratic 2bR model
Allosaurus tc2 data, fit by a Power 2R model
Psittacosaurus m1 data, fit by a Quadratic 2bR model
Age (yr)
Bone dimension (cm)

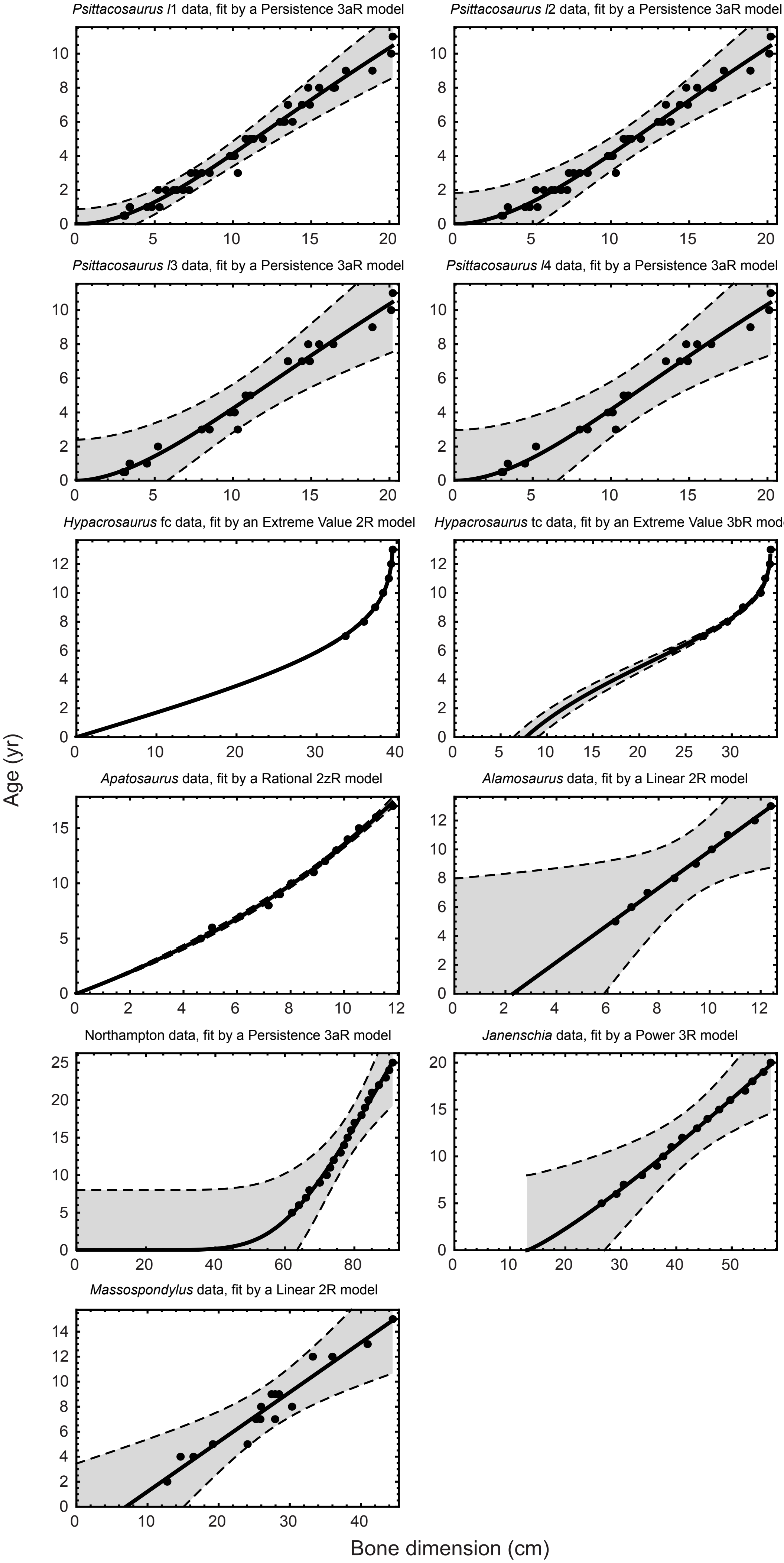
Psittacosaurus l1 data, fit by a Persistence 3aR model
Psittacosaurus l2 data, fit by a Persistence 3aR model
Psittacosaurus l3 data, fit by a Persistence 3aR model
Psittacosaurus l4 data, fit by a Persistence 3aR model
Hypacrosaurus fc data, fit by an Extreme Value 2R model
Hypacrosaurus tc data, fit by an Extreme Value 3bR model
Apatosaurus data, fit by a Rational 2zR model
Alamosaurus data, fit by a Linear 2R model
Northampton data, fit by a Persistence 3aR model
Janenschia data, fit by a Power 3R model
Massospondylus data, fit by a Linear 2R model
Age (yr)
Bone dimension (cm)
