# Supplementary material for: Revisiting the Estimation of Dinosaur Growth Rates
Source: PLoS One. 2013 Dec 16;8(12):e81917. doi: 10.1371/journal.pone.0081917 (PMC3864909; doi:10.1371/journal.pone.0081917)
Supplement: Table S1 — Functional form of growth functions . This table includes both asymptotic functions, which approach a finite asymptote of , and increasing functions for which . For each growth function, its inverse function is also presented (if , then ), along with several properties of the function such as the starting value , the growth rate , the age at the inflection point , the size at the inflection point , and the maximum growth rate (which occurs at the inflection point). The inflection point and related functions are meaningful for sigmoidal curves, as they are not defined for increasing or attenuating curves. The constraints given define the parameter values for which the growth function are defined and real-valued. Most growth models in the literature employ three or four parameters. Sample size (m) is small for many of the dinosaur data sets, however, so it is important to include variations of the functions that include just two parameters. Although a two-parameter model is less flexible in fitting data patterns than is a model having more parameters, a two-parameter model can yield statistically valid results even when fit to as few as five data points. The corrected Akaike information criterion (AICc) is infinite whenever the number of parameters is not less than . N.M.: not meaningful. (DOCX) [file pone.0081917.s013.docx]

| Beta-P4  Kind: asymptotic sigmoidal  References: [1–3] | | | | | | |  |
| --- | --- | --- | --- | --- | --- | --- | --- |
| Function | Equation | Constraints | | | | |  |
| $g\left(t\right)$ | $a\left(1-\left(1+\left(\frac{t}{c}\right)^{d}\right)^{-b}\right)$ | $a>0$ | $b>0$ | | $c>0$ | $d>0$ | |
| $h\left(y\right)$ | $c\left(-1+\left(-\frac{-a+y}{a}\right)^{-1/b}\right)^{\frac{1}{d}}$ | $a>y$ | $b>0$ | | $c>0$ | $d>0$ | |
| $g\left(0\right)$ | 0 |  | |  | | |  |
| $\frac{\text{d}g\left(\text{t}\right)}{\text{d}t}$ | $\frac{abd\left(\frac{t}{c}\right)^{d}\left(1+\left(\frac{t}{c}\right)^{d}\right)^{-1-b}}{t}$ |  | |  | | |  |
| $t_{\text{inf}}$ | $c\left(\frac{-1+d}{1+bd}\right)^{\frac{1}{d}}$ |  | |  | | |  |
| $g\left(t_{\text{inf}}\right)$ | $a-a\left(\frac{1+bd}{d+bd}\right)^{b}$ |  | |  | | |  |
| $\frac{\text{d}g\left(t_{\text{inf}}\right)}{\text{d}t}$ | $\frac{ab\left(-1+d\right)^{\frac{-1+d}{d}}d\left(\left(1+b\right)d\right)^{-1-b}\left(1+bd\right)^{b+\frac{1}{d}}}{c}$ | | |  | | |  |

| Chapman-Richards 3  Kind: asymptotic sigmoidal  References: [2,4–7] | | | | | |
| --- | --- | --- | --- | --- | --- |
| Function | Equation | Constraints | | | |
| $g\left(t\right)$ | $a\left(1-\text{e}^{-bt}\right)^{c}$ | $a>0$ | $b>0$ | | $c>0$ |
| $h\left(y\right)$ | $-\frac{\text{Log}\left(1-\left(\frac{y}{a}\right)^{\frac{1}{c}}\right)}{b}$ | $a>y$ | $b>0$ | | $c>0$ |
| $g\left(0\right)$ | 0 |  | |  | |
| $\frac{\text{d}g\left(\text{t}\right)}{\text{d}t}$ | $abc\text{e}^{-bt}\left(1-\text{e}^{-bt}\right)^{-1+c}$ |  | |  | |
| $t_{\text{inf}}$ | $\frac{\text{Log}\left(c\right)}{b}$ |  | |  | |
| $g\left(t_{\text{inf}}\right)$ | $a\left(\frac{-1+c}{c}\right)^{c}$ |  | |  | |
| $\frac{\text{d}g\left(t_{\text{inf}}\right)}{\text{d}t}$ | $ab\left(\frac{-1+c}{c}\right)^{-1+c}$ |  | |  | |

| Chapman-Richards 4  Kind: asymptotic sigmoidal  References: [2,4–7] | | | | | | | |
| --- | --- | --- | --- | --- | --- | --- | --- |
| Function | Equation | Constraints | | | | | |
| $g\left(t\right)$ | $d+\left(a-d\right)\left(1-\text{e}^{-bt}\right)^{c}$ | $a>0$ | $b>0$ | | $c>0$ | | $a>d$ |
| $h\left(y\right)$ | $\frac{\text{Log}\left[\frac{1}{1-\left(\frac{-d+y}{a-d}\right)^{\frac{1}{c}}}\right]}{b}$ | $a>y$ | $b>0$ | | $c>0$ | | $y>d$ |
| $g\left(0\right)$ | $d$ |  | |  | |  | |
| $\frac{\text{d}g\left(\text{t}\right)}{\text{d}t}$ | $bc\left(a-d\right)\text{e}^{-bct}\left(-1+\text{e}^{bt}\right)^{-1+c}$ |  | |  | |  | |
| $t_{\text{inf}}$ | $\frac{\text{Log}\left[c\right]}{b}$ |  | |  | |  | |
| $g\left(t_{\text{inf}}\right)$ | $\left(\frac{-1+c}{c}\right)^{c}\left(a-d\right)+d$ |  | |  | |  | |
| $\frac{\text{d}g\left(t_{\text{inf}}\right)}{\text{d}t}$ | $b\left(\frac{-1+c}{c}\right)^{-1+c}\left(a-d\right)$ |  | |  | |  | |

| Extreme Value 3a  Kind: asymptotic sigmoidal  References: [2,7,8] | | | | |
| --- | --- | --- | --- | --- |
| Function | Equation | Constraints | | |
| $g\left(t\right)$ | $a\left(1-\text{e}^{-\text{e}^{c+bt}}\right)$ | $a>0$ | $b>0$ |  |
| $h\left(y\right)$ | $\frac{-c+\text{Log}\left[-\text{Log}\left[-\frac{-a+y}{a}\right]\right]}{b}$ | $a>y$ | $b>0$ |  |
| $g\left(0\right)$ | $a-a\text{e}^{-\text{e}^{c}}$ |  |  |  |
| $\frac{\text{d}g\left(\text{t}\right)}{\text{d}t}$ | $ab\text{e}^{c-\text{e}^{c+bt}+bt}$ |  |  |  |
| $t_{\text{inf}}$ | $-\frac{c}{b}$ |  |  |  |
| $g\left(t_{\text{inf}}\right)$ | $\frac{a\left(-1+\text{e}\right)}{\text{e}}$ |  |  |  |
| $\frac{\text{d}g\left(t_{\text{inf}}\right)}{\text{d}t}$ | $\frac{ab}{\text{e}}$ |  |  |  |

| Extreme Value 3b  Kind: asymptotic sigmoidal  References: [2,7,8] | | | | |
| --- | --- | --- | --- | --- |
| Function | Equation | Constraints | | |
| $g\left(t\right)$ | $a\left(1-\text{e}^{-\text{e}^{b\left(c+t\right)}}\right)$ | $a>0$ | $b>0$ |  |
| $h\left(y\right)$ | $\frac{-bc+\text{Log}\left[-\text{Log}\left[-\frac{-a+y}{a}\right]\right]}{b}$ | $a>y$ | $b>0$ |  |
| $g\left(0\right)$ | $a-a\text{e}^{-\text{e}^{bc}}$ |  |  |  |
| $\frac{\text{d}g\left(\text{t}\right)}{\text{d}t}$ | $ab\text{e}^{-\text{e}^{b\left(c+t\right)}+b\left(c+t\right)}$ |  |  |  |
| $t_{\text{inf}}$ | $-c$ |  |  |  |
| $g\left(t_{\text{inf}}\right)$ | $\frac{a\left(-1+\text{e}\right)}{\text{e}}$ |  |  |  |
| $\frac{\text{d}g\left(t_{\text{inf}}\right)}{\text{d}t}$ | $\frac{ab}{\text{e}}$ |  |  |  |

| Extreme Value 3z  Kind: asymptotic sigmoidal  References: [2,7,8] | | | | |
| --- | --- | --- | --- | --- |
| Function | Equation | Constraints | | |
| $g\left(t\right)$ | $a\left(1-\text{e}^{\text{e}^{c}-\text{e}^{c+bt}}\right)$ | $a>0$ | $b>0$ |  |
| $h\left(y\right)$ | $\frac{-c+\text{Log}\left[\text{e}^{c}-\text{Log}\left[-\frac{-a+y}{a}\right]\right]}{b}$ | $a>y$ | $b>0$ |  |
| $g\left(0\right)$ | $0$ |  |  |  |
| $\frac{\text{d}g\left(\text{t}\right)}{\text{d}t}$ | $ab\text{e}^{c+\text{e}^{c}-\text{e}^{c+bt}+bt}$ |  |  |  |
| $t_{\text{inf}}$ | $-\frac{c}{b}$ |  |  |  |
| $g\left(t_{\text{inf}}\right)$ | $a-a\text{e}^{-1+\text{e}^{c}}$ |  |  |  |
| $\frac{\text{d}g\left(t_{\text{inf}}\right)}{\text{d}t}$ | $ab\text{e}^{-1+\text{e}^{c}}$ |  |  |  |

| Extreme Value 4  Kind: asymptotic sigmoidal  References: [2,7,8] | | | | |
| --- | --- | --- | --- | --- |
| Function | Equation | Constraints | | |
| $g\left(t\right)$ | $d+\left(a-d\right)\left(1-\text{e}^{-\text{e}^{c+bt}}\right)$ | $a>0$ | $b>0$ | $a>d$ |
| $h\left(y\right)$ | $\frac{-c+\text{Log}\left[\text{Log}\left[\frac{a-d}{a-y}\right]\right]}{b}$ | $a>y$ | $b>0$ | $y>d$ |
| $g\left(0\right)$ | $a+\left(-a+d\right)\text{e}^{-\text{e}^{c}}$ |  |  |  |
| $\frac{\text{d}g\left(\text{t}\right)}{\text{d}t}$ | $b\left(a-d\right)\text{e}^{c-\text{e}^{c+bt}+bt}$ |  |  |  |
| $t_{\text{inf}}$ | $-\frac{c}{b}$ |  |  |  |
| $g\left(t_{\text{inf}}\right)$ | $\frac{d+a\left(-1+\text{e}\right)}{\text{e}}$ |  |  |  |
| $\frac{\text{d}g\left(t_{\text{inf}}\right)}{\text{d}t}$ | $\frac{b\left(a-d\right)}{\text{e}}$ |  |  |  |

| Gompertz 3a  Kind: asymptotic sigmoidal  References: [4,9–11] | | | | |
| --- | --- | --- | --- | --- |
| Function | Equation | Constraints | | |
| $g\left(t\right)$ | $a\text{e}^{-b\text{e}^{-ct}}$ | $a>0$ | $b>0$ | $c>0$ |
| $h\left(y\right)$ | $-\frac{\text{Log}\left[-\frac{\text{Log}\left[\frac{y}{a}\right]}{b}\right]}{c}$ | $a>y$ | $b>0$ | $c>0$ |
| $g\left(0\right)$ | $a\text{e}^{-b}$ |  |  |  |
| $\frac{\text{d}g\left(\text{t}\right)}{\text{d}t}$ | $abc\text{e}^{-b\text{e}^{-ct}-ct}$ |  |  |  |
| $t_{\text{inf}}$ | $\frac{\text{Log}\left[b\right]}{c}$ |  |  |  |
| $g\left(t_{\text{inf}}\right)$ | $\frac{a}{\text{e}}$ |  |  |  |
| $\frac{\text{d}g\left(t_{\text{inf}}\right)}{\text{d}t}$ | $\frac{ac}{\text{e}}$ |  |  |  |

| Gompertz 3b  Kind: asymptotic sigmoidal  References: [4,9–11] | | | | |
| --- | --- | --- | --- | --- |
| Function | Equation | Constraints | | |
| $g\left(t\right)$ | $a\text{e}^{-\text{e}^{-b\left(-c+t\right)}}$ | $a>0$ | $b>0$ |  |
| $h\left(y\right)$ | $\frac{bc-\text{Log}\left[-\text{Log}\left[\frac{y}{a}\right]\right]}{b}$ | $a>y$ | $b>0$ |  |
| $g\left(0\right)$ | $a\text{e}^{-\text{e}^{bc}}$ |  |  |  |
| $\frac{\text{d}g\left(\text{t}\right)}{\text{d}t}$ | $ab\text{e}^{-\text{e}^{b\left(c-t\right)}+b\left(c-t\right)}$ |  |  |  |
| $t_{\text{inf}}$ | $c$ |  |  |  |
| $g\left(t_{\text{inf}}\right)$ | $\frac{a}{\text{e}}$ |  |  |  |
| $\frac{\text{d}g\left(t_{\text{inf}}\right)}{\text{d}t}$ | $\frac{ab}{\text{e}}$ |  |  |  |

| Gompertz 3z  Kind: asymptotic sigmoidal  References: [4,9–11] | | | | |
| --- | --- | --- | --- | --- |
| Function | Equation | Constraints | | |
| $g\left(t\right)$ | $\frac{a\left(-1+\text{e}^{b-b\text{e}^{-ct}}\right)}{-1+\text{e}^{b}}$ | $a>0$ | $b>0$ | $c>0$ |
| $h\left(y\right)$ | $-\frac{\text{Log}\left[-\frac{-b+\text{Log}\left[-\frac{\left(1-\text{e}^{b}\right)\left(-\frac{a}{1-\text{e}^{b}}+y\right)}{a}\right]}{b}\right]}{c}$ | $a>y$ | $b>0$ | $c>0$ |
| $g\left(0\right)$ | $0$ |  |  |  |
| $\frac{\text{d}g\left(\text{t}\right)}{\text{d}t}$ | $\frac{abc\text{e}^{b-b\text{e}^{-ct}-ct}}{-1+\text{e}^{b}}$ |  |  |  |
| $t_{\text{inf}}$ | $\frac{\text{Log}\left[b\right]}{c}$ |  |  |  |
| $g\left(t_{\text{inf}}\right)$ | $\frac{a\left(-1+\text{e}^{-1+b}\right)}{-1+\text{e}^{b}}$ |  |  |  |
| $\frac{\text{d}g\left(t_{\text{inf}}\right)}{\text{d}t}$ | $\frac{ac\text{e}^{-1+b}}{-1+\text{e}^{b}}$ |  |  |  |

| Gompertz 4a  Kind: asymptotic sigmoidal  References: [4,9–11] | | | | | | | |
| --- | --- | --- | --- | --- | --- | --- | --- |
| Function | Equation | Constraints | | | | | |
| $g\left(t\right)$ | $d+\left(a-d\right)\text{e}^{-b\text{e}^{-ct}}$ | $a>0$ | $b>0$ | | $c>0$ | | $a>d$ |
| $h\left(y\right)$ | $\frac{\text{Log}\left[\frac{b}{\text{Log}\left[\frac{-a+d}{d-y}\right]}\right]}{c}$ | $a>y$ | $b>0$ | | $c>0$ | | $y>d$ |
| $g\left(0\right)$ | $d+\left(a-d\right)\text{e}^{-b}$ |  | |  | |  | |
| $\frac{\text{d}g\left(\text{t}\right)}{\text{d}t}$ | $bc\left(a-d\right)\text{e}^{-b\text{e}^{-ct}-ct}$ |  | |  | |  | |
| $t_{\text{inf}}$ | $\frac{\text{Log}\left[b\right]}{c}$ |  | |  | |  | |
| $g\left(t_{\text{inf}}\right)$ | $d+\frac{a-d}{\text{e}}$ |  | |  | |  | |
| $\frac{\text{d}g\left(t_{\text{inf}}\right)}{\text{d}t}$ | $\frac{c\left(a-d\right)}{\text{e}}$ |  | |  | |  | |

| Gompertz 4b  Kind: asymptotic sigmoidal  References: [4,9–11] | | | | |
| --- | --- | --- | --- | --- |
| Function | Equation | Constraints | | |
| $g\left(t\right)$ | $d+\left(a-d\right)\text{e}^{-\text{e}^{-b\left(-c+t\right)}}$ | $a>0$ | $b>0$ | $a>d$ |
| $h\left(y\right)$ | $\frac{bc-\text{Log}\left[\text{Log}\left[\frac{-a+d}{d-y}\right]\right]}{b}$ | $a>y$ | $b>0$ | $y>d$ |
| $g\left(0\right)$ | $d+\left(a-d\right)\text{e}^{-\text{e}^{bc}}$ |  |  |  |
| $\frac{\text{d}g\left(\text{t}\right)}{\text{d}t}$ | $b\left(a-d\right)\text{e}^{-\text{e}^{b\left(c-t\right)}+b\left(c-t\right)}$ |  |  |  |
| $t_{\text{inf}}$ | $c$ |  |  |  |
| $g\left(t_{\text{inf}}\right)$ | $d+\frac{a-d}{\text{e}}$ |  |  |  |
| $\frac{\text{d}g\left(t_{\text{inf}}\right)}{\text{d}t}$ | $\frac{b\left(a-d\right)}{\text{e}}$ |  |  |  |

| He Legendre 2  Kind: asymptotic sigmoidal  References: [12,13] | | | | |
| --- | --- | --- | --- | --- |
| Function | Equation | Constraints | | |
| $g\left(t\right)$ | $\frac{a}{1+t^{-b}}$ | $a>0$ | $b>0$ |  |
| $h\left(y\right)$ | $\left(-1+\frac{a}{y}\right)^{-1/b}$ | $a>y$ | $b>0$ |  |
| $g\left(0\right)$ | $0$ |  |  |  |
| $\frac{\text{d}g\left(\text{t}\right)}{\text{d}t}$ | $\frac{abt^{-1+b}}{\left(1+t^{b}\right)^{2}}$ |  |  |  |
| $t_{\text{inf}}$ | $\left(\frac{-1+b}{1+b}\right)^{\frac{1}{b}}$ |  |  |  |
| $g\left(t_{\text{inf}}\right)$ | $\frac{a\left(-1+b\right)}{2b}$ |  |  |  |
| $\frac{\text{d}g\left(t_{\text{inf}}\right)}{\text{d}t}$ | $\frac{a\left(-1+b\right)^{1-\frac{1}{b}}\left(1+b\right)^{1+\frac{1}{b}}}{4b}$ |  |  |  |

| He Legendre 3  Kind: asymptotic sigmoidal  References: [12,13] | | | | |
| --- | --- | --- | --- | --- |
| Function | Equation | Constraints | | |
| $g\left(t\right)$ | $\frac{ab}{b+t^{-c}}$ | $a>0$ | $b>0$ | $c>0$ |
| $h\left(y\right)$ | $\left(-b+\frac{ab}{y}\right)^{-1/c}$ | $a>y$ | $b>0$ | $c>0$ |
| $g\left(0\right)$ | $0$ |  |  |  |
| $\frac{\text{d}g\left(\text{t}\right)}{\text{d}t}$ | $\frac{abct^{-1+c}}{\left(1+bt^{c}\right)^{2}}$ |  |  |  |
| $t_{\text{inf}}$ | $\left(\frac{-1+c}{b\left(1+c\right)}\right)^{\frac{1}{c}}$ |  |  |  |
| $g\left(t_{\text{inf}}\right)$ | $\frac{a\left(-1+c\right)}{2c}$ |  |  |  |
| $\frac{\text{d}g\left(t_{\text{inf}}\right)}{\text{d}t}$ | $\frac{a\left(-1+c\right)^{\frac{-1+c}{c}}\left(1+c\right)\left(b\left(1+c\right)\right)^{\frac{1}{c}}}{4c}$ |  |  |  |

| He Legendre 4  Kind: asymptotic sigmoidal  References: [12,13] | | | | | | | |
| --- | --- | --- | --- | --- | --- | --- | --- |
| Function | Equation | Constraints | | | | | |
| $g\left(t\right)$ | $d+\frac{b\left(a-d\right)}{b+t^{-c}}$ | $a>0$ | $b>0$ | | $c>0$ | | $a>d$ |
| $h\left(y\right)$ | $\left(\frac{d-y}{b\left(-a+y\right)}\right)^{\frac{1}{c}}$ | $a>y$ | $b>0$ | | $c>0$ | | $y>d$ |
| $g\left(0\right)$ | $d$ |  | |  | |  | |
| $\frac{\text{d}g\left(\text{t}\right)}{\text{d}t}$ | $\frac{bc\left(a-d\right)t^{-1+c}}{\left(1+bt^{c}\right)^{2}}$ |  | |  | |  | |
| $t_{\text{inf}}$ | $\left(\frac{-1+c}{b\left(1+c\right)}\right)^{\frac{1}{c}}$ |  | |  | |  | |
| $g\left(t_{\text{inf}}\right)$ | $\frac{a\left(-1+c\right)+d+cd}{2c}$ |  | |  | |  | |
| $\frac{\text{d}g\left(t_{\text{inf}}\right)}{\text{d}t}$ | $\frac{\left(\frac{-1+c}{b\left(1+c\right)}\right)^{\frac{-1}{c}}\left(-1+c^{2}\right)\left(a-d\right)}{4c}$ |  | |  | |  | |

| Korf 3  Kind: asymptotic sigmoidal  Reference: [14] | | | | |
| --- | --- | --- | --- | --- |
| Function | Equation | Constraints | | |
| $g\left(t\right)$ | $a\text{e}^{-bt^{-c}}$ | $a>0$ | $b>0$ | $c>0$ |
| $h\left(y\right)$ | $\left(-\frac{\text{Log}\left[\frac{y}{a}\right]}{b}\right)^{-1/c}$ | $a>y$ | $b>0$ | $c>0$ |
| $g\left(0\right)$ | $0$ |  |  |  |
| $\frac{\text{d}g\left(\text{t}\right)}{\text{d}t}$ | $abc\text{e}^{-bt^{-c}}t^{-1-c}$ |  |  |  |
| $t_{\text{inf}}$ | $\left(\frac{1+c}{bc}\right)^{\frac{-1}{c}}$ |  |  |  |
| $g\left(t_{\text{inf}}\right)$ | $a\text{e}^{-1-\frac{1}{c}}$ |  |  |  |
| $\frac{\text{d}g\left(t_{\text{inf}}\right)}{\text{d}t}$ | $a\left(1+c\right)\left(\frac{1+c}{bc}\right)^{\frac{1}{c}}\text{e}^{-1-\frac{1}{c}}$ |  |  |  |

| Korf 4  Kind: asymptotic sigmoidal  Reference: [14] | | | | | | | |
| --- | --- | --- | --- | --- | --- | --- | --- |
| Function | Equation | Constraints | | | | | |
| $g\left(t\right)$ | $d+\left(a-d\right)\text{e}^{-bt^{-c}}$ | $a>0$ | $b>0$ | | $c>0$ | | $a>d$ |
| $h\left(y\right)$ | $\left(\frac{b}{\text{Log}\left[\frac{-a+d}{d-y}\right]}\right)^{\frac{1}{c}}$ | $a>y$ | $b>0$ | | $c>0$ | | $y>d$ |
| $g\left(0\right)$ | $d$ |  | |  | |  | |
| $\frac{\text{d}g\left(\text{t}\right)}{\text{d}t}$ | $bc\left(a-d\right)\text{e}^{-bt^{-c}}t^{-1-c}$ |  | |  | |  | |
| $t_{\text{inf}}$ | $\left(\frac{1+c}{bc}\right)^{-1/c}$ |  | |  | |  | |
| $g\left(t_{\text{inf}}\right)$ | $d+\left(a-d\right)\text{e}^{-1-\frac{1}{c}}$ |  | |  | |  | |
| $\frac{\text{d}g\left(t_{\text{inf}}\right)}{\text{d}t}$ | $\left(1+c\right)\left(\frac{1+c}{bc}\right)^{\frac{1}{c}}\left(a-d\right)\text{e}^{-1-\frac{1}{c}}$ |  | |  | |  | |

| Levakovic 2  Kind: asymptotic sigmoidal  Reference: [14] | | | | |
| --- | --- | --- | --- | --- |
| Function | Equation | Constraints | | |
| $g\left(t\right)$ | $a\left(\frac{t^{2}}{1+t^{2}}\right)^{b}$ | $a>0$ | $b>0$ |  |
| $h\left(y\right)$ |  | $a>y$ | $b>0$ |  |
| $g\left(0\right)$ | $0$ |  |  |  |
| $\frac{\text{d}g\left(\text{t}\right)}{\text{d}t}$ | $2abt^{-1+2b}\left(1+t^{2}\right)^{-1-b}$ |  |  |  |
| $t_{\text{inf}}$ | $\frac{\sqrt{-1+2b}}{\sqrt{3}}$ |  |  |  |
| $g\left(t_{\text{inf}}\right)$ | $a\left(1-\frac{3}{2\left(1+b\right)}\right)^{b}$ |  |  |  |
| $\frac{\text{d}g\left(t_{\text{inf}}\right)}{\text{d}t}$ | $3\times2^{-b}\sqrt{3}ab\left(1+b\right)^{-1-b}\left(-1+2b\right)^{-\frac{1}{2}+b}$ |  |  |  |

| Levakovic 3a  Kind: asymptotic sigmoidal  Reference: [14] | | | | |
| --- | --- | --- | --- | --- |
| Function | Equation | Constraints | | |
| $g\left(t\right)$ | $a\left(\frac{t^{2}}{b+t^{2}}\right)^{c}$ | $a>0$ | $b>0$ | $c>0$ |
| $h\left(y\right)$ | $\frac{\sqrt{b}\left(\frac{y}{a}\right)^{\frac{1}{2c}}}{\sqrt{1-\left(\frac{y}{a}\right)^{\frac{1}{c}}}}$ | $a>y$ | $b>0$ | $c>0$ |
| $g\left(0\right)$ | $0$ |  |  |  |
| $\frac{\text{d}g\left(\text{t}\right)}{\text{d}t}$ | $\frac{2abc\left(\frac{t^{2}}{b+t^{2}}\right)^{1+c}}{t^{3}}$ |  |  |  |
| $t_{\text{inf}}$ | $\frac{\sqrt{b\left(-1+2c\right)}}{\sqrt{3}}$ |  |  |  |
| $g\left(t_{\text{inf}}\right)$ | $a\left(1-\frac{3}{2\left(1+c\right)}\right)^{c}$ |  |  |  |
| $\frac{\text{d}g\left(t_{\text{inf}}\right)}{\text{d}t}$ | $3\sqrt{3}a\left(-\frac{1}{2}+c\right)^{c}c\left(1+c\right)^{-1-c}\sqrt{-\frac{1}{b-2bc}}$ |  |  |  |

| Levakovic 3b  Kind: asymptotic sigmoidal  Reference: [14] | | | | |
| --- | --- | --- | --- | --- |
| Function | Equation | Constraints | | |
| $g\left(t\right)$ | $a\left(\frac{t^{b}}{1+t^{b}}\right)^{c}$ | $a>0$ | $b>0$ | $c>0$ |
| $h\left(y\right)$ | $\left(-\left(\frac{y}{a}\right)^{-1/c}\left(-1+\left(\frac{y}{a}\right)^{\frac{1}{c}}\right)\right)^{-1/b}$ | $a>y$ | $b>0$ | $c>0$ |
| $g\left(0\right)$ | $0$ |  |  |  |
| $\frac{\text{d}g\left(\text{t}\right)}{\text{d}t}$ | $abct^{-1+bc}\left(1+t^{b}\right)^{-1-c}$ |  |  |  |
| $t_{\text{inf}}$ | $\left(\frac{-1+bc}{1+b}\right)^{\frac{1}{b}}$ |  |  |  |
| $g\left(t_{\text{inf}}\right)$ | $a\left(\frac{-1+bc}{b+bc}\right)^{c}$ |  |  |  |
| $\frac{\text{d}g\left(t_{\text{inf}}\right)}{\text{d}t}$ | $ab^{-c}\left(1+b\right)^{1+\frac{1}{b}}c\left(1+c\right)^{-1-c}\left(-1+bc\right)^{-\frac{1}{b}+c}$ |  |  |  |

| Levakovic 4a  Kind: asymptotic sigmoidal  Reference: [14] | | | | | | | |
| --- | --- | --- | --- | --- | --- | --- | --- |
| Function | Equation | Constraints | | | | | |
| $g\left(t\right)$ | $d+\left(a-d\right)\left(\frac{t^{2}}{b+t^{2}}\right)^{c}$ | $a>0$ | $b>0$ | | $c>0$ | | $a>d$ |
| $h\left(y\right)$ | $\frac{\sqrt{b}\sqrt{\left(\frac{-d+y}{a-d}\right)^{\frac{1}{c}}}}{\sqrt{1-\left(\frac{-d+y}{a-d}\right)^{\frac{1}{c}}}}$ | $a>y$ | $b>0$ | | $c>0$ | | $y>d$ |
| $g\left(0\right)$ | $d$ |  | |  | |  | |
| $\frac{\text{d}g\left(\text{t}\right)}{\text{d}t}$ | $\frac{2bc\left(a-d\right)\left(\frac{t^{2}}{b+t^{2}}\right)^{1+c}}{t^{3}}$ |  | |  | |  | |
| $t_{\text{inf}}$ | $\frac{\sqrt{b}\sqrt{-1+2c}}{\sqrt{3}}$ |  | |  | |  | |
| $g\left(t_{\text{inf}}\right)$ | $\left(1-\frac{3}{2\left(1+c\right)}\right)^{c}\left(a-d\right)+d$ |  | |  | |  | |
| $\frac{\text{d}g\left(t_{\text{inf}}\right)}{\text{d}t}$ | $3\sqrt{3}\left(-\frac{1}{2}+c\right)^{c}c\left(1+c\right)^{-1-c}\sqrt{-\frac{1}{b-2bc}}\left(a-d\right)$ |  | |  | |  | |

| Levakovic 4b  Kind: asymptotic sigmoidal  Reference: [14] | | | | | | | |
| --- | --- | --- | --- | --- | --- | --- | --- |
| Function | Equation | Constraints | | | | | |
| $g\left(t\right)$ | $a\left(\frac{t^{d}}{b+t^{d}}\right)^{c}$ | $a>0$ | $b>0$ | | $c>0$ | | $d>0$ |
| $h\left(y\right)$ | $\left(-\frac{\left(\frac{y}{a}\right)^{-1/c}\left(-1+\left(\frac{y}{a}\right)^{\frac{1}{c}}\right)}{b}\right)^{-1/d}$ | $a>y$ | $b>0$ | | $c>0$ | | $d>0$ |
| $g\left(0\right)$ | $0$ |  | |  | |  | |
| $\frac{\text{d}g\left(\text{t}\right)}{\text{d}t}$ | $abcdt^{-1+cd}\left(b+t^{d}\right)^{-1-c}$ |  | |  | |  | |
| $t_{\text{inf}}$ | $\left(\frac{b\left(-1+cd\right)}{1+d}\right)^{\frac{1}{d}}$ |  | |  | |  | |
| $g\left(t_{\text{inf}}\right)$ | $a\left(\frac{-1+cd}{d+cd}\right)^{c}$ |  | |  | |  | |
| $\frac{\text{d}g\left(t_{\text{inf}}\right)}{\text{d}t}$ | $abcd\left(\frac{b\left(1+c\right)d}{1+d}\right)^{-1-c}\left(\frac{b\left(-1+cd\right)}{1+d}\right)^{c-\frac{1}{d}}$ |  | |  | |  | |

| Logistic 2b  Kind: asymptotic sigmoidal  References: [4,15–17] | | | | |
| --- | --- | --- | --- | --- |
| Function | Equation | Constraints | | |
| $g\left(t\right)$ | $-1+\frac{\left(1+a\right)\text{e}^{bt}}{a+\text{e}^{bt}}$ | $a>0$ | $b>0$ |  |
| $h\left(y\right)$ | $\frac{\text{Log}\left[\frac{\left(-1+a\right)y}{a-y}\right]}{b}$ | $a>y$ | $b>0$ |  |
| $g\left(0\right)$ | $0$ |  |  |  |
| $\frac{\text{d}g\left(\text{t}\right)}{\text{d}t}$ | $\frac{a\left(1+a\right)b\text{e}^{bt}}{\left(a+\text{e}^{bt}\right)^{2}}$ |  |  |  |
| $t_{\text{inf}}$ | $\frac{\text{Log}\left[a\right]}{b}$ |  |  |  |
| $g\left(t_{\text{inf}}\right)$ | $\frac{1}{2}\left(-1+a\right)$ |  |  |  |
| $\frac{\text{d}g\left(t_{\text{inf}}\right)}{\text{d}t}$ | $\frac{1}{4}\left(1+a\right)b$ |  |  |  |

| Logistic 3  Kind: asymptotic sigmoidal  References: [4,15–17] | | | | |
| --- | --- | --- | --- | --- |
| Function | Equation | Constraints | | |
| $g\left(t\right)$ | $\frac{a}{1+\text{e}^{-b\left(-c+t\right)}}$ | $a>0$ | $b>0$ |  |
| $h\left(y\right)$ | $\frac{bc-\text{Log}\left[-1+\frac{a}{y}\right]}{b}$ | $a>y$ | $b>0$ |  |
| $g\left(0\right)$ | $\frac{a}{1+\text{e}^{bc}}$ |  |  |  |
| $\frac{\text{d}g\left(\text{t}\right)}{\text{d}t}$ | $\frac{ab\text{e}^{b\left(c+t\right)}}{\left(\text{e}^{bc}+\text{e}^{bt}\right)^{2}}$ |  |  |  |
| $t_{\text{inf}}$ | $c$ |  |  |  |
| $g\left(t_{\text{inf}}\right)$ | $\frac{a}{2}$ |  |  |  |
| $\frac{\text{d}g\left(t_{\text{inf}}\right)}{\text{d}t}$ | $\frac{ab}{4}$ |  |  |  |

| Logistic 3z  Kind: asymptotic sigmoidal  References: [4,15–17] | | | | |
| --- | --- | --- | --- | --- |
| Function | Equation | Constraints | | |
| $g\left(t\right)$ | $\frac{a\left(-1+\text{e}^{bt}\right)}{\text{e}^{bc}+\text{e}^{bt}}$ | $a>0$ | $b>0$ |  |
| $h\left(y\right)$ | $\frac{\text{Log}\left[\frac{a+\text{e}^{bc}y}{a-y}\right]}{b}$ | $a>y$ | $b>0$ |  |
| $g\left(0\right)$ | $0$ |  |  |  |
| $\frac{\text{d}g\left(\text{t}\right)}{\text{d}t}$ | $\frac{ab\text{e}^{bt}\left(1+\text{e}^{bc}\right)}{\left(\text{e}^{bc}+\text{e}^{bt}\right)^{2}}$ |  |  |  |
| $t_{\text{inf}}$ | $c$ |  |  |  |
| $g\left(t_{\text{inf}}\right)$ | $\frac{1}{2}a\left(1-\text{e}^{-bc}\right)$ |  |  |  |
| $\frac{\text{d}g\left(t_{\text{inf}}\right)}{\text{d}t}$ | $\frac{1}{4}ab\left(1+\text{e}^{-bc}\right)$ |  |  |  |

| Logistic 4a  Kind: asymptotic sigmoidal  References: [4,15–17] | | | | |
| --- | --- | --- | --- | --- |
| Function | Equation | Constraints | | |
| $g\left(t\right)$ | $d+\frac{a-d}{1+\text{e}^{-b\left(-c+t\right)}}$ | $a>0$ | $b>0$ | $a>d$ |
| $h\left(y\right)$ | $\frac{bc-\text{Log}\left[\frac{-a+y}{d-y}\right]}{b}$ | $a>y$ | $b>0$ | $y>d$ |
| $g\left(0\right)$ | $d+\frac{a-d}{1+\text{e}^{bc}}$ |  |  |  |
| $\frac{\text{d}g\left(\text{t}\right)}{\text{d}t}$ | $\frac{b\left(a-d\right)\text{e}^{b\left(c+t\right)}}{\left(\text{e}^{bc}+\text{e}^{bt}\right)^{2}}$ |  |  |  |
| $t_{\text{inf}}$ | $c$ |  |  |  |
| $g\left(t_{\text{inf}}\right)$ | $\frac{a+d}{2}$ |  |  |  |
| $\frac{\text{d}g\left(t_{\text{inf}}\right)}{\text{d}t}$ | $\frac{1}{4}b\left(a-d\right)$ |  |  |  |

| Lomolino 2  Kind: asymptotic sigmoidal  References: [2,18] | | | | |
| --- | --- | --- | --- | --- |
| Function | Equation | Constraints | | |
| $g\left(t\right)$ | $\frac{a}{1+b^{\text{Log}\left[\frac{1}{t}\right]}}$ | $a>0$ | $b>1$ |  |
| $h\left(y\right)$ | $\left(-1+\frac{a}{y}\right)^{-\frac{1}{\text{Log}\left[b\right]}}$ | $a>y$ | $b>1$ |  |
| $g\left(0\right)$ | $0$ |  |  |  |
| $\frac{\text{d}g\left(\text{t}\right)}{\text{d}t}$ | $\frac{ab^{\text{Log}\left[t\right]}\text{Log}\left[b\right]}{\left(1+b^{\text{Log}\left[t\right]}\right)^{2}t}$ |  |  |  |
| $t_{\text{inf}}$ | $\left(1-\frac{2}{1+\text{Log}\left[b\right]}\right)^{\frac{1}{\text{Log}\left[b\right]}}$ |  |  |  |
| $g\left(t_{\text{inf}}\right)$ | $\frac{a}{1+b^{\text{Log}\left[\left(-1+\text{Log}\left[b\right]\right)^{-\frac{1}{\text{Log}\left[b\right]}}\right]\left(1+\text{Log}\left[b\right]\right)}}$ |  |  |  |
| $\frac{\text{d}g\left(t_{\text{inf}}\right)}{\text{d}t}$ | $\frac{ab^{\text{Log}\left[\left(-1+\text{Log}\left[b\right]\right)^{\frac{1}{\text{Log}\left[b\right]}}\right]}\text{Log}\left[b\right]\left(1+\text{Log}\left[b\right]\right)\left(1-\frac{2}{1+\text{Log}\left[b\right]}\right)^{-\frac{1}{\text{Log}\left[b\right]}}}{1+\text{b}^{\text{Log}\left[\left(-1+\text{Log}\left[b\right]\right)^{\frac{1}{\text{Log}\left[b\right]}}\right]}+\text{Log}\left[b\right]}$ | | |  |

| Lomolino 3  Kind: asymptotic sigmoidal  References: [2,18] | | | | |
| --- | --- | --- | --- | --- |
| Function | Equation | Constraints | | |
| $g\left(t\right)$ | $\frac{a}{1+b^{\text{Log}\left[\frac{c}{t}\right]}}$ | $a>0$ | $b>1$ | $c>0$ |
| $h\left(y\right)$ | $c\left(-1+\frac{a}{y}\right)^{-\frac{1}{\text{Log}\left[b\right]}}$ | $a>y$ | $b>1$ | $c>0$ |
| $g\left(0\right)$ | $0$ |  |  |  |
| $\frac{\text{d}g\left(\text{t}\right)}{\text{d}t}$ | $\frac{ab^{\text{Log}\left[ct\right]}\text{Log}\left[b\right]}{\left(b^{\text{Log}\left[c\right]}+b^{\text{Log}\left[t\right]}\right)^{2}t}$ |  |  |  |
| $t_{\text{inf}}$ | $\left(\frac{b^{\text{Log}\left[c\right]}\left(-1+\text{Log}\left[b\right]\right)}{1+\text{Log}\left[b\right]}\right)^{\frac{1}{\text{Log}\left[b\right]}}$ |  |  |  |
| $g\left(t_{\text{inf}}\right)$ | $\frac{a}{1+b^{\text{Log}\left[\left(-1+\text{Log}\left[b\right]\right)^{-\frac{1}{\text{Log}\left[b\right]}}\right]}\left(1+\text{Log}\left[b\right]\right)}$ |  |  |  |
| $\frac{\text{d}g\left(t_{\text{inf}}\right)}{\text{d}t}$ | $\frac{ab^{\text{Log}\left[c\left(\frac{b^{\text{Log}\left[c\right]}\left(-1+\text{Log}\left[b\right]\right)}{1+\text{Log}\left[b\right]}\right)^{\frac{1}{\text{Log}\left[b\right]}}\right]}\text{Log}\left[b\right]\left(\frac{b^{\text{Log}\left[c\right]}\left(-1+\text{Log}\left[b\right]\right)}{1+\text{Log}\left[b\right]}\right)^{-\frac{1}{\text{Log}\left[b\right]}}}{\left(b^{\text{Log}\left[c\right]}+b^{\text{Log}\left[\left(\frac{b^{\text{Log}\left[c\right]}\left(-1+\text{Log}\left[b\right]\right)}{1+\text{Log}\left[b\right]}\right)^{\frac{1}{\text{Log}\left[b\right]}}\right]}\right)^{2}}$ | | |  |

| Lomolino 4  Kind: asymptotic sigmoidal  References: [2,18] | | | | | | | |
| --- | --- | --- | --- | --- | --- | --- | --- |
| Function | Equation | Constraints | | | | | |
| $g\left(t\right)$ | $\frac{a-d}{1+b^{\text{Log}\left[\frac{c}{t}\right]}}+d$ | $a>0$ | $b>1$ | | $c>0$ | | $a>d$ |
| $h\left(y\right)$ | $c\left(\frac{-a+y}{d-y}\right)^{-\frac{1}{\text{Log}\left[b\right]}}$ | $a>y$ | $b>1$ | | $c>0$ | | $y>d$ |
| $g\left(0\right)$ | $d$ |  | |  | |  | |
| $\frac{\text{d}g\left(\text{t}\right)}{\text{d}t}$ | $\frac{b^{\text{Log}\left[ct\right]}\left(a-d\right)\text{Log}\left[b\right]}{\left(b^{\text{Log}\left[c\right]}+b^{\text{Log}\left[t\right]}\right)^{2}t}$ |  | |  | |  | |
| $t_{\text{inf}}$ | $\left(\frac{b^{\text{Log}\left[c\right]}\left(-1+\text{Log}\left[b\right]\right)}{1+\text{Log}\left[b\right]}\right)^{\frac{1}{\text{Log}\left[b\right]}}$ |  | |  | |  | |
| $g\left(t_{\text{inf}}\right)$ | $d+\frac{a-d}{1+b^{\text{Log}\left[\left(-1+\text{Log}\left[b\right]\right)^{-\frac{1}{\text{Log}\left[b\right]}}\right]}\left(1+\text{Log}\left[b\right]\right)}$ |  | |  | |  | |
| $\frac{\text{d}g\left(t_{\text{inf}}\right)}{\text{d}t}$ | $\frac{b^{\text{Log}\left[c\left(\frac{b^{\text{Log}\left[c\right]}\left(-1+\text{Log}\left[b\right]\right)}{1+\text{Log}\left[b\right]}\right)^{\frac{1}{\text{Log}\left[b\right]}}\right]}\left(a-d\right)\text{Log}\left[b\right]\left(\frac{b^{\text{Log}\left[c\right]}\left(-1+\text{Log}\left[b\right]\right)}{1+\text{Log}\left[b\right]}\right)^{-\frac{1}{\text{Log}\left[b\right]}}}{\left(b^{\text{Log}\left[c\right]}+b^{\text{Log}\left[\left(\frac{b^{\text{Log}\left[c\right]}\left(-1+\text{Log}\left[b\right]\right)}{1+\text{Log}\left[b\right]}\right)^{\frac{1}{\text{Log}\left[b\right]}}\right]}\right)^{2}}$ | | | | |  | |

| Morgan-Mercer-Flodin 2  Kind: asymptotic sigmoidal  References: [2,19–21] | | | | |
| --- | --- | --- | --- | --- |
| Function | Equation | Constraints | | |
| $g\left(t\right)$ | $\frac{at^{b}}{1+t^{b}}$ | $a>0$ | $b>0$ |  |
| $h\left(y\right)$ | $\left(-\frac{-a+y}{y}\right)^{-1/b}$ | $a>y$ | $b>0$ |  |
| $g\left(0\right)$ | $0$ |  |  |  |
| $\frac{\text{d}g\left(\text{t}\right)}{\text{d}t}$ | $\frac{abt^{-1+b}}{\left(1+t^{b}\right)^{2}}$ |  |  |  |
| $t_{\text{inf}}$ | $\left(\frac{-1+b}{1+b}\right)^{\frac{1}{b}}$ |  |  |  |
| $g\left(t_{\text{inf}}\right)$ | $\frac{a\left(-1+b\right)}{2b}$ |  |  |  |
| $\frac{\text{d}g\left(t_{\text{inf}}\right)}{\text{d}t}$ | $\frac{a\left(-1+b\right)^{1-\frac{1}{b}}\left(1+b\right)^{1+\frac{1}{b}}}{4b}$ |  |  |  |

| Morgan-Mercer-Flodin 3a  Kind: asymptotic sigmoidal  References: [2,19–21] | | | | |
| --- | --- | --- | --- | --- |
| Function | Equation | Constraints | | |
| $g\left(t\right)$ | $\frac{at^{c}}{b+t^{c}}$ | $a>0$ | $b>0$ | $c>0$ |
| $h\left(y\right)$ | $\left(-\frac{-a+y}{by}\right)^{-1/c}$ | $a>y$ | $b>0$ | $c>0$ |
| $g\left(0\right)$ | $0$ |  |  |  |
| $\frac{\text{d}g\left(\text{t}\right)}{\text{d}t}$ | $\frac{abct^{-1+c}}{\left(b+t^{c}\right)^{2}}$ |  |  |  |
| $t_{\text{inf}}$ | $0$ |  |  |  |
| $g\left(t_{\text{inf}}\right)$ | $0$ |  |  |  |
| $\frac{\text{d}g\left(t_{\text{inf}}\right)}{\text{d}t}$ | $0$ |  |  |  |

| Morgan-Mercer-Flodin 3b  Kind: asymptotic sigmoidal  References: [2,19–21] | | | | |
| --- | --- | --- | --- | --- |
| Function | Equation | Constraints | | |
| $g\left(t\right)$ | $a-\frac{a}{1+\left(bt\right)^{c}}$ | $a>0$ | $b>0$ | $c>0$ |
| $h\left(y\right)$ | $\frac{\left(-1+\frac{a}{a-y}\right)^{\frac{1}{c}}}{b}$ | $a>y$ | $b>0$ | $c>0$ |
| $g\left(0\right)$ | $0$ |  |  |  |
| $\frac{\text{d}g\left(\text{t}\right)}{\text{d}t}$ | $\frac{ac\left(bt\right)^{c}}{t\left(1+\left(bt\right)^{c}\right)^{2}}$ |  |  |  |
| $t_{\text{inf}}$ | $\frac{\left(\frac{-1+c}{1+c}\right)^{\frac{1}{c}}}{b}$ |  |  |  |
| $g\left(t_{\text{inf}}\right)$ | $\frac{a\left(-1+c\right)}{2c}$ |  |  |  |
| $\frac{\text{d}g\left(t_{\text{inf}}\right)}{\text{d}t}$ | $\frac{ab\left(-1+c\right)^{1-\frac{1}{c}}\left(1+c\right)^{1+\frac{1}{c}}}{4c}$ |  |  |  |

| Morgan-Mercer-Flodin 4  Kind: asymptotic sigmoidal  References: [2,19–21] | | | | | | | |
| --- | --- | --- | --- | --- | --- | --- | --- |
| Function | Equation | Constraints | | | | | |
| $g\left(t\right)$ | $d+\frac{\left(a-d\right)t^{c}}{b+t^{c}}$ | $a>0$ | $b>0$ | | $c>0$ | | $a>d$ |
| $h\left(y\right)$ | $\left(\frac{-bd+by}{a-y}\right)^{\frac{1}{c}}$ | $a>y$ | $b>0$ | | $c>0$ | | $y\geq d$ |
| $g\left(0\right)$ | $d$ |  | |  | |  | |
| $\frac{\text{d}g\left(\text{t}\right)}{\text{d}t}$ | $\frac{bc\left(a-d\right)t^{-1+c}}{\left(b+t^{c}\right)^{2}}$ |  | |  | |  | |
| $t_{\text{inf}}$ | $0$ |  | |  | |  | |
| $g\left(t_{\text{inf}}\right)$ | $d$ |  | |  | |  | |
| $\frac{\text{d}g\left(t_{\text{inf}}\right)}{\text{d}t}$ | $0$ |  | |  | |  | |

| Morgan-Mercer-Flodin 4b  Kind: asymptotic sigmoidal  References: [2,19–21] | | | | | | | |
| --- | --- | --- | --- | --- | --- | --- | --- |
| Function | Equation | Constraints | | | | | |
| $g\left(t\right)$ | $a-\frac{a-d}{1+\left(bt\right)^{c}}$ | $a>0$ | $b>0$ | | $c>0$ | | $a>d$ |
| $h\left(y\right)$ | $\frac{\left(\frac{-d+y}{a-y}\right)^{\frac{1}{c}}}{b}$ | $a>y$ | $b>0$ | | $c>0$ | | $y>d$ |
| $g\left(0\right)$ | $d$ |  | |  | |  | |
| $\frac{\text{d}g\left(\text{t}\right)}{\text{d}t}$ | $\frac{c\left(a-d\right)\left(bt\right)^{c}}{t\left(1+\left(bt\right)^{c}\right)^{2}}$ |  | |  | |  | |
| $t_{\text{inf}}$ | $\frac{\left(\frac{-1+c}{1+c}\right)^{\frac{1}{c}}}{b}$ |  | |  | |  | |
| $g\left(t_{\text{inf}}\right)$ | $\frac{a\left(-1+c\right)+d+cd}{2c}$ |  | |  | |  | |
| $\frac{\text{d}g\left(t_{\text{inf}}\right)}{\text{d}t}$ | $\frac{b\left(-1+c\right)^{\frac{-1+c}{c}}\left(1+c\right)^{1+\frac{1}{c}}\left(a-d\right)}{4c}$ |  | |  | |  | |

| Richards 4a  Kind: asymptotic sigmoidal  References: [22–24] | | | | | | | |
| --- | --- | --- | --- | --- | --- | --- | --- |
| Function | Equation | Constraints | | | | | |
| $g\left(t\right)$ | $a\left(1-b\text{e}^{-ct}\right)^{d}$ | $a>0$ | $b>0$ | | $c>0$ | | $d\geq1$ |
| $h\left(y\right)$ | $-\frac{\text{Log}\left[\frac{1-\left(\frac{y}{a}\right)^{\frac{1}{d}}}{b}\right]}{c}$ | $a>y$ | $b>0$ | | $c>0$ | | $d\geq1$ |
| $g\left(0\right)$ | $a\left(1-b\right)^{d}$ |  | |  | |  | |
| $\frac{\text{d}g\left(\text{t}\right)}{\text{d}t}$ | $abcd\text{e}^{-ct}\left(1-b\text{e}^{-ct}\right)^{-1+d}$ |  | |  | |  | |
| $t_{\text{inf}}$ | $\frac{\text{Log}\left[bd\right]}{c}$ |  | |  | |  | |
| $g\left(t_{\text{inf}}\right)$ | $a\left(\frac{-1+d}{d}\right)^{d}$ |  | |  | |  | |
| $\frac{\text{d}g\left(t_{\text{inf}}\right)}{\text{d}t}$ | $ac\left(\frac{-1+d}{d}\right)^{-1+d}$ |  | |  | |  | |

| Richards 4b  Kind: asymptotic sigmoidal  References: [22–24] | | | | | | | |
| --- | --- | --- | --- | --- | --- | --- | --- |
| Function | Equation | Constraints | | | | | |
| $g\left(t\right)$ | $a\left(1+b\text{e}^{-ct}\right)^{-d}$ | $a>0$ | $b>0$ | | $c>0$ | | $d>0$ |
| $h\left(y\right)$ | $-\frac{\text{Log}\left[-\frac{1-\left(\frac{y}{a}\right)^{-1/d}}{b}\right]}{c}$ | $a>y$ | $b>0$ | | $c>0$ | | $d>0$ |
| $g\left(0\right)$ | $a\left(1+b\right)^{-d}$ |  | |  | |  | |
| $\frac{\text{d}g\left(\text{t}\right)}{\text{d}t}$ | $abcd\text{e}^{cdt}\left(b+\text{e}^{ct}\right)^{-1-d}$ |  | |  | |  | |
| $t_{\text{inf}}$ | $\frac{\text{Log}\left[bd\right]}{c}$ |  | |  | |  | |
| $g\left(t_{\text{inf}}\right)$ | $a\left(\frac{d}{1+d}\right)^{d}$ |  | |  | |  | |
| $\frac{\text{d}g\left(t_{\text{inf}}\right)}{\text{d}t}$ | $ac\left(\frac{d}{1+d}\right)^{1+d}$ |  | |  | |  | |

| Richards 4c  Kind: asymptotic sigmoidal  References: [22–24] | | | | | | | |
| --- | --- | --- | --- | --- | --- | --- | --- |
| Function | Equation | Constraints | | | | | |
| $g\left(t\right)$ | $a\left(1-\text{e}^{-b\left(-c+t\right)}\right)^{d}$ | $a>0$ | $b>0$ | | $t>c$ | | $d\geq1$ |
| $h\left(y\right)$ | $\frac{bc-\text{Log}\left[1-\left(\frac{y}{a}\right)^{\frac{1}{d}}\right]}{b}$ | $a>y$ | $b>0$ | | $d\geq1$ | |  |
| $g\left(0\right)$ | $a\left(1-\text{e}^{bc}\right)^{d}$ |  | |  | |  | |
| $\frac{\text{d}g\left(\text{t}\right)}{\text{d}t}$ | $\frac{abd\left(1-\text{e}^{b\left(c-t\right)}\right)^{d}}{-1+\text{e}^{b\left(-c+t\right)}}$ |  | |  | |  | |
| $t_{\text{inf}}$ | $c+\frac{\text{Log}\left[d\right]}{b}$ |  | |  | |  | |
| $g\left(t_{\text{inf}}\right)$ | $a\left(\frac{-1+d}{d}\right)^{d}$ |  | |  | |  | |
| $\frac{\text{d}g\left(t_{\text{inf}}\right)}{\text{d}t}$ | $ab\left(\frac{-1+d}{d}\right)^{-1+d}$ |  | |  | |  | |

| Richards 4d  Kind: asymptotic sigmoidal  References: [22–24] | | | | |
| --- | --- | --- | --- | --- |
| Function | Equation | Constraints | | |
| $g\left(t\right)$ | $a\left(1+\text{e}^{-b\left(-c+t\right)}\right)^{-d}$ | $a>0$ | $b>0$ | $d\geq0$ |
| $h\left(y\right)$ | $\frac{bc-\text{Log}\left[-1+\left(\frac{y}{a}\right)^{-1/d}\right]}{b}$ | $a>y$ | $b>0$ | $d>0$ |
| $g\left(0\right)$ | $a\left(1+\text{e}^{bc}\right)^{-d}$ |  |  |  |
| $\frac{\text{d}g\left(\text{t}\right)}{\text{d}t}$ | $abd\text{e}^{b\left(c-t\right)}\left(1+\text{e}^{b\left(c-t\right)}\right)^{-1-d}$ |  |  |  |
| $t_{\text{inf}}$ | $c+\frac{\text{Log}\left[d\right]}{b}$ |  |  |  |
| $g\left(t_{\text{inf}}\right)$ | $a\left(\frac{d}{1+d}\right)^{d}$ |  |  |  |
| $\frac{\text{d}g\left(t_{\text{inf}}\right)}{\text{d}t}$ | $ab\left(1+\frac{1}{d}\right)^{-1-d}$ |  |  |  |

| Richards 4e  Kind: asymptotic sigmoidal  References: [22–24] | | | | | | | |  |
| --- | --- | --- | --- | --- | --- | --- | --- | --- |
| Function | Equation | Constraints | | | | | |  |
| $g\left(t\right)$ | $a\left(1+\left(-1+\left(\frac{c}{a}\right)^{1-d}\right)\text{e}^{-bd^{-\frac{d}{1-d}}t}\right)^{\frac{1}{1-d}}$ | $a>0$ | $b>0$ | | $a>c>0$ | | $d>0$ | |
| $h\left(y\right)$ | $\frac{d^{\frac{d}{1-d}}\text{Log}\left[-\frac{\left(\frac{c}{a}\right)^{-d}\left(-c+a\left(\frac{c}{a}\right)^{d}\right)\left(\frac{y}{a}\right)^{d}}{y-a\left(\frac{y}{a}\right)^{d}}\right]}{b}$ | $a>y$ | $b>0$ | | $a>c>0$ | | $d>0$ | |
| $g\left(0\right)$ | $c$ |  | |  | |  | |  |
| $\frac{\text{d}g\left(\text{t}\right)}{\text{d}t}$ | $-\frac{b\left(a-\left(\frac{a}{c}\right)^{d}c\right)d^{\frac{d}{-1+d}}\text{e}^{-bd^{\frac{d}{-1+d}}t}\left(1+\left(-1+\left(\frac{a}{c}\right)^{-1+d}\right)\text{e}^{-bd^{\frac{d}{-1+d}}t}\right)^{\frac{d}{1-d}}}{-1+d}$ | | | | |  | |  |
| $t_{\text{inf}}$ | $\frac{d^{-\frac{d}{-1+d}}\left(\text{Log}\left[\frac{a-\left(\frac{a}{c}\right)^{d}c}{a-ad}\right]\right)}{b}$ |  | |  | |  | |  |
| $g\left(t_{\text{inf}}\right)$ | $ad^{\frac{1}{1-d}}$ |  | |  | |  | |  |
| $\frac{\text{d}g\left(t_{\text{inf}}\right)}{\text{d}t}$ | $ab$ |  | |  | |  | |  |

| Sloboda 4  Kind: asymptotic sigmoidal  References: [14] | | | | | | | |
| --- | --- | --- | --- | --- | --- | --- | --- |
| Function | Equation | Constraints | | | | | |
| $g\left(t\right)$ | $a\text{e}^{-b\text{e}^{-ct^{d}}}$ | $a>0$ | $b>0$ | | $c>0$ | | $d>0$ |
| $h\left(y\right)$ | $-\left(\frac{c}{\text{Log}\left[-\frac{\text{Log}\left[\frac{y}{a}\right]}{b}\right]}\right)^{-1/d}$ | $a>y$ | $b>0$ | | $c>0$ | | $d>0$ |
| $g\left(0\right)$ | $a\text{e}^{-b}$ |  | |  | |  | |
| $\frac{\text{d}g\left(\text{t}\right)}{\text{d}t}$ | $abcd\text{e}^{-b\text{e}^{-ct^{d}}-ct^{d}}t^{-1+d}$ |  | |  | |  | |
| $t_{\text{inf}}$ | no closed form |  | |  | |  | |
| $g\left(t_{\text{inf}}\right)$ | no closed form |  | |  | |  | |
| $\frac{\text{d}g\left(t_{\text{inf}}\right)}{\text{d}t}$ | no closed form |  | |  | |  | |

| von Bertalanffy 2  Kind: asymptotic sigmoidal  References: [25,26] | | | | |
| --- | --- | --- | --- | --- |
| Function | Equation | Constraints | | |
| $g\left(t\right)$ | $a\left(1-\text{e}^{-bt}\right)^{3}$ | $a>0$ | $b>0$ |  |
| $h\left(y\right)$ | $-\frac{\text{Log}\left[1-\frac{y^{1/3}}{a^{1/3}}\right]}{b}$ | $a>\text{y}$ | $b>0$ |  |
| $g\left(0\right)$ | $0$ |  |  |  |
| $\frac{\text{d}g\left(\text{t}\right)}{\text{d}t}$ | $3ab\text{e}^{-3bt}\left(-1+\text{e}^{bt}\right)^{2}$ |  |  |  |
| $t_{\text{inf}}$ | $\frac{\text{Log}\left[3\right]}{b}$ |  |  |  |
| $g\left(t_{\text{inf}}\right)$ | $\frac{8a}{27}$ |  |  |  |
| $\frac{\text{d}g\left(t_{\text{inf}}\right)}{\text{d}t}$ | $\frac{4ab}{9}$ |  |  |  |

| von Bertalanffy 3  Kind: asymptotic sigmoidal  References: [25,26] | | | | |
| --- | --- | --- | --- | --- |
| Function | Equation | Constraints | | |
| $g\left(t\right)$ | $a\left(1-\text{e}^{-b\left(-c+t\right)}\right)^{3}$ | $a>0$ | $b>0$ |  |
| $h\left(y\right)$ | $\frac{bc-\text{Log}\left[1-\frac{y^{1/3}}{a^{1/3}}\right]}{b}$ | $a>y$ | $b>0$ |  |
| $g\left(0\right)$ | $-a\left(-1+\text{e}^{bc}\right)^{3}$ |  |  |  |
| $\frac{\text{d}g\left(\text{t}\right)}{\text{d}t}$ | $3ab\text{e}^{b\left(c-t\right)}\left(-1+\text{e}^{b\left(c-t\right)}\right)^{2}$ |  |  |  |
| $t_{\text{inf}}$ | $c+\frac{\text{Log}\left[3\right]}{b}$ |  |  |  |
| $g\left(t_{\text{inf}}\right)$ | $\frac{8a}{27}$ |  |  |  |
| $\frac{\text{d}g\left(t_{\text{inf}}\right)}{\text{d}t}$ | $\frac{4ab}{9}$ |  |  |  |

| von Bertalanffy 3z  Kind: asymptotic sigmoidal  References: [25,26] | | | | | |
| --- | --- | --- | --- | --- | --- |
| Function | Equation | Constraints | | | |
| $g\left(t\right)$ | $\frac{a\left(3-3\text{e}^{bc}+\text{e}^{2bc}+3\text{e}^{b\left(c-2t\right)}-3\text{e}^{-bt}-\text{e}^{2bc-3bt}\right)}{3-3\text{e}^{bc}+\text{e}^{2bc}}$ | $a>0$ | $b>0$ | | $c\leq0$ |
| $h\left(y\right)$ | $-\frac{\text{Log}\left[e^{-bc}\left(1-\left[-\left(-1+e^{bc}\right)^{3}+\frac{\left(1+\left(-1+e^{bc}\right)^{3}\right)y}{a}\right]^{1/3}\right)\right]}{b}$ | $a>y$ | $b>0$ | | $c\leq0$ |
| $g\left(0\right)$ | $0$ |  | |  | |
| $\frac{\text{d}g\left(\text{t}\right)}{\text{d}t}$ | $\frac{3ab\text{e}^{-3bt}\left(\text{e}^{bc}-\text{e}^{bt}\right)^{2}}{3-3\text{e}^{bc}+\text{e}^{2bc}}$ |  | |  | |
| $t_{\text{inf}}$ | $c+\frac{\text{Log}\left[3\right]}{b}$ |  | |  | |
| $g\left(t_{\text{inf}}\right)$ | $\frac{1}{81}a\left(81-19\text{e}^{-bc}+\frac{19\left(-3+\text{e}^{bc}\right)}{3-3\text{e}^{bc}+\text{e}^{2bc}}\right)$ |  | |  | |
| $\frac{\text{d}g\left(t_{\text{inf}}\right)}{\text{d}t}$ | $\frac{4ab\text{e}^{-bc}}{9\left(3-3\text{e}^{bc}+\text{e}^{2bc}\right)}$ |  | |  | |

| von Bertalanffy 4  Kind: asymptotic sigmoidal  References: [25,26] | | | | | | | |
| --- | --- | --- | --- | --- | --- | --- | --- |
| Function | Equation | Constraints | | | | | |
| $g\left(t\right)$ | $d+\left(a-d\right)\left(1-\text{e}^{-b\left(-c+t\right)}\right)^{3}$ | $a>0$ | $b>0$ | | $a>d$ | | $c\leq0$ |
| $h\left(y\right)$ | $c-\frac{\text{Log}\left[1-\left(\frac{-d+y}{a-d}\right)^{1/3}\right]}{b}$ | $a>y$ | | $b>0$ | | $c\leq0$ | |
| $g\left(0\right)$ | $-a\left(-1+\text{e}^{bc}\right)^{3}+d\text{e}^{bc}\left(3-3\text{e}^{bc}+\text{e}^{2bc}\right)$ |  | |  | |  | |
| $\frac{\text{d}g\left(\text{t}\right)}{\text{d}t}$ | $3b\left(a-d\right)\text{e}^{b\left(c-t\right)}\left(-1+\text{e}^{b\left(c-t\right)}\right)^{2}$ |  | |  | |  | |
| $t_{\text{inf}}$ | $c+\frac{\text{Log}\left[3\right]}{b}$ |  | |  | |  | |
| $g\left(t_{\text{inf}}\right)$ | $\frac{1}{27}\left(8a+19d\right)$ |  | |  | |  | |
| $\frac{\text{d}g\left(t_{\text{inf}}\right)}{\text{d}t}$ | $\frac{4}{9}b\left(a-d\right)$ |  | |  | |  | |

| Weibull 3a  Kind: asymptotic sigmoidal  References: [2,15,27,28] | | | | |
| --- | --- | --- | --- | --- |
| Function | Equation | Constraints | | |
| $g\left(t\right)$ | $a\left(1-\text{e}^{-bt^{c}}\right)$ | $a>0$ | $b>0$ | $c>0$ |
| $h\left(y\right)$ | $\left(-\frac{\text{Log}\left[-\frac{-a+y}{a}\right]}{b}\right)^{\frac{1}{c}}$ | $a>y$ | $b>0$ | $c>0$ |
| $g\left(0\right)$ | $0$ |  |  |  |
| $\frac{\text{d}g\left(\text{t}\right)}{\text{d}t}$ | $abc\text{e}^{-bt^{c}}t^{-1+c}$ |  |  |  |
| $t_{\text{inf}}$ | $\left(-\frac{1-c}{bc}\right)^{\frac{1}{c}}$ |  |  |  |
| $g\left(t_{\text{inf}}\right)$ | $a-a\text{e}^{-1+\frac{1}{c}}$ |  |  |  |
| $\frac{\text{d}g\left(t_{\text{inf}}\right)}{\text{d}t}$ | $a\left(-1+c\right)^{1-\frac{1}{c}}\left(bc\right)^{\frac{1}{c}}\text{e}^{-1+\frac{1}{c}}$ |  |  |  |

| Weibull 3b  Kind: asymptotic sigmoidal  References: [2,15,27,28] | | | | |
| --- | --- | --- | --- | --- |
| Function | Equation | Constraints | | |
| $g\left(t\right)$ | $a\left(1-\text{e}^{-\left(bt\right)^{c}}\right)$ | $a>0$ | $b>0$ | $c>0$ |
| $h\left(y\right)$ | $\frac{\left(-\text{Log}\left[-\frac{-a+y}{a}\right]\right)^{\frac{1}{c}}}{b}$ | $a>y$ | $b>0$ | $c>0$ |
| $g\left(0\right)$ | $0$ |  |  |  |
| $\frac{\text{d}g\left(\text{t}\right)}{\text{d}t}$ | $ab^{c}c\text{e}^{-\left(bt\right)^{c}}t^{-1+c}$ |  |  |  |
| $t_{\text{inf}}$ | $\frac{\left(\frac{-1+c}{c}\right)^{\frac{1}{c}}}{b}$ |  |  |  |
| $g\left(t_{\text{inf}}\right)$ | $a-a\text{e}^{-1+\frac{1}{c}}$ |  |  |  |
| $\frac{\text{d}g\left(t_{\text{inf}}\right)}{\text{d}t}$ | $ab\left(\frac{-1+c}{c}\right)^{\frac{-1+c}{c}}ce^{-1+\frac{1}{c}}$ |  |  |  |

| Weibull 4a  Kind: asymptotic sigmoidal  References: [2,15,27,28] | | | | | | | |
| --- | --- | --- | --- | --- | --- | --- | --- |
| Function | Equation | Constraints | | | | | |
| $g\left(t\right)$ | $d+\left(a-d\right)\left(1-\text{e}^{-bt^{c}}\right)$ | $a>0$ | $b>0$ | | $c>0$ | | $a>d$ |
| $h\left(y\right)$ | $\left(\frac{\text{Log}\left[\frac{a-d}{a-y}\right]}{b}\right)^{\frac{1}{c}}$ | $a>y$ | $b>0$ | | $c>0$ | | $y>d$ |
| $g\left(0\right)$ | $d$ |  | |  | |  | |
| $\frac{\text{d}g\left(\text{t}\right)}{\text{d}t}$ | $bc\left(a-d\right)\text{e}^{-bt^{c}}t^{-1+c}$ |  | |  | |  | |
| $t_{\text{inf}}$ | $\left(-\frac{1-c}{bc}\right)^{\frac{1}{c}}$ |  | |  | |  | |
| $g\left(t_{\text{inf}}\right)$ | $a+\left(-a+d\right)\text{e}^{-1+\frac{1}{c}}$ |  | |  | |  | |
| $\frac{\text{d}g\left(t_{\text{inf}}\right)}{\text{d}t}$ | $\left(-1+c\right)\left(\frac{-1+c}{bc}\right)^{-1/c}\left(a-d\right)\text{e}^{-1+\frac{1}{c}}$ |  | |  | |  | |

| Weibull 4b  Kind: asymptotic sigmoidal  References: [2,15,27,28] | | | | | | | | | |
| --- | --- | --- | --- | --- | --- | --- | --- | --- | --- |
| Function | Equation | Constraints | | | | | | | |
| $g\left(t\right)$ | $a\left(1-\text{e}^{-\left(b\left(-c+t\right)\right)^{d}}\right)$ | $a>0$ | $b>0$ | | | $\text{t}>c$ | | | $d>0$ |
| $h\left(y\right)$ | $\frac{bc+\left(-\text{Log}\left[-\frac{-a+y}{a}\right]\right)^{\frac{1}{d}}}{b}$ | $a>y$ | | | $b>0$ | | | $d>0$ | |
| $g\left(0\right)$ | $a-a\text{e}^{-\left(-bc\right)^{d}}$ |  | |  | | |  | | |
| $\frac{\text{d}g\left(\text{t}\right)}{\text{d}t}$ | $abd\text{e}^{-\left(b\left(-c+t\right)\right)^{d}}\left(b\left(-c+t\right)\right)^{-1+d}$ |  | |  | | |  | | |
| $t_{\text{inf}}$ | $c+\frac{\left(\frac{-1+d}{d}\right)^{\frac{1}{d}}}{b}$ |  | |  | | |  | | |
| $g\left(t_{\text{inf}}\right)$ | $a-a\text{e}^{-1+\frac{1}{d}}$ |  | |  | | |  | | |
| $\frac{\text{d}g\left(t_{\text{inf}}\right)}{\text{d}t}$ | $ab\left(\frac{-1+d}{d}\right)^{\frac{-1+d}{d}}d\text{e}^{-1+\frac{1}{d}}$ |  | |  | | |  | | |

| Extreme Value 2  Kind: asymptotic attenuating  References: [2,7,8] | | | | |
| --- | --- | --- | --- | --- |
| Function | Equation | Constraints | | |
| $g\left(t\right)$ | $a-a\text{e}^{1-\text{e}^{bt}}$ | $a>0$ | $b>0$ |  |
| $h\left(y\right)$ | $\frac{\text{Log}\left[1+\text{Log}\left[\frac{a}{a-y}\right]\right]}{b}$ | $a>y$ | $b>0$ |  |
| $g\left(0\right)$ | $0$ |  |  |  |
| $\frac{\text{d}g\left(\text{t}\right)}{\text{d}t}$ | $ab\text{e}^{1-\text{e}^{bt}+bt}$ |  |  |  |
| $t_{\text{inf}}$ | N.M. |  |  |  |
| $g\left(t_{\text{inf}}\right)$ | N.M. |  |  |  |
| $\frac{\text{d}g\left(t_{\text{inf}}\right)}{\text{d}t}$ | N.M. |  |  |  |

| Gompertz 2  Kind: asymptotic attenuating  References: [4,9–11] | | | | |
| --- | --- | --- | --- | --- |
| Function | Equation | Constraints | | |
| $g\left(t\right)$ | $\frac{a\left(-1+\text{e}^{1-\text{e}^{-bt}} \right)}{\left(-1+\text{e}\right)}$ | $a>0$ | $b>0$ |  |
| $h\left(y\right)$ | $-\frac{\text{Log}\left[\text{Log}\frac{ae}{a+\left(-1+e\right)y}\right]}{b}$ | $a>y$ | $b>0$ |  |
| $g\left(0\right)$ | $0$ |  |  |  |
| $\frac{\text{d}g\left(\text{t}\right)}{\text{d}t}$ | $\frac{ab\text{e}^{1-e^{-bt}-bt}}{-1+e}$ |  |  |  |
| $t_{\text{inf}}$ | N.M. |  |  |  |
| $g\left(t_{\text{inf}}\right)$ | N.M. |  |  |  |
| $\frac{\text{d}g\left(t_{\text{inf}}\right)}{\text{d}t}$ | N.M. |  |  |  |

| Logistic 2a  Kind: asymptotic attenuating  References: [4,15–17] | | | | |
| --- | --- | --- | --- | --- |
| Function | Equation | Constraints | | |
| $g\left(t\right)$ | $2a\left(-\frac{1}{2}+\frac{1}{1+\text{e}^{-bt}}\right)$ | $a>0$ | $b>0$ |  |
| $h\left(y\right)$ | $\frac{\text{Log}\left[\frac{a+y}{a-y}\right]}{b}$ | $a>y$ | $b>0$ |  |
| $g\left(0\right)$ | $0$ |  |  |  |
| $\frac{\text{d}g\left(\text{t}\right)}{\text{d}t}$ | $\frac{ab}{1+\text{Cosh}\left[bt\right]}$ |  |  |  |
| $t_{\text{inf}}$ | N.M. |  |  |  |
| $g\left(t_{\text{inf}}\right)$ | N.M. |  |  |  |
| $\frac{\text{d}g\left(t_{\text{inf}}\right)}{\text{d}t}$ | N.M. |  |  |  |

| Michaelis Menten 2  Kind: asymptotic attenuating  References: [29] | | | | |
| --- | --- | --- | --- | --- |
| Function | Equation | Constraints | | |
| $g\left(t\right)$ | $\frac{at}{b+t}$ | $a>0$ | $b>0$ |  |
| $h\left(y\right)$ | $\frac{by}{a-y}$ | $a>y$ | $b>0$ |  |
| $g\left(0\right)$ | $0$ |  |  |  |
| $\frac{\text{d}g\left(\text{t}\right)}{\text{d}t}$ | $\frac{ab}{\left(b+t\right)^{2}}$ |  |  |  |
| $t_{\text{inf}}$ | N.M. |  |  |  |
| $g\left(t_{\text{inf}}\right)$ | N.M. |  |  |  |
| $\frac{\text{d}g\left(t_{\text{inf}}\right)}{\text{d}t}$ | N.M. |  |  |  |

| Michaelis Menten 3  Kind: asymptotic attenuating  References: [29] | | | | |
| --- | --- | --- | --- | --- |
| Function | Equation | Constraints | | |
| $g\left(t\right)$ | $c+\frac{\left(a-c\right)t}{b+t}$ | $a>0$ | $b>0$ | $a>c$ |
| $h\left(y\right)$ | $\frac{-bc+by}{a-y}$ | $a>y$ | $b>0$ | $a>c$ |
| $g\left(0\right)$ | $c$ |  |  |  |
| $\frac{\text{d}g\left(\text{t}\right)}{\text{d}t}$ | $\frac{b\left(a-c\right)}{\left(b+t\right)^{2}}$ |  |  |  |
| $t_{\text{inf}}$ | N.M. |  |  |  |
| $g\left(t_{\text{inf}}\right)$ | N.M. |  |  |  |
| $\frac{\text{d}g\left(t_{\text{inf}}\right)}{\text{d}t}$ | N.M. |  |  |  |

| Monomolecular 3  Kind: asymptotic attenuating  Reference: [14] | | | | |
| --- | --- | --- | --- | --- |
| Function | Equation | Constraints | | |
| $g\left(t\right)$ | $a\left(1-c\text{e}^{-bt}\right)$ | $a>0$ | $b>0$ | $c>0$ |
| $h\left(y\right)$ | $-\frac{\text{Log}\left[-\frac{-a+y}{ac}\right]}{b}$ | $a>y$ | $b>0$ | $c>0$ |
| $g\left(0\right)$ | $a-ac$ |  |  |  |
| $\frac{\text{d}g\left(\text{t}\right)}{\text{d}t}$ | $abc\text{e}^{-bt}$ |  |  |  |
| $t_{\text{inf}}$ | N.M. |  |  |  |
| $g\left(t_{\text{inf}}\right)$ | N.M. |  |  |  |
| $\frac{\text{d}g\left(t_{\text{inf}}\right)}{\text{d}t}$ | N.M. |  |  |  |

| Negative Exponential 2a  Kind: increasing | | | | |
| --- | --- | --- | --- | --- |
| Function | Equation | Constraints | | |
| $g\left(t\right)$ | $a\left(1-\text{e}^{-bt}\right)$ | $a>0$ | $b>0$ |  |
| $h\left(y\right)$ | $-\frac{\text{Log}\left[-\frac{-a+y}{a}\right]}{b}$ | $a>y$ | $b>0$ |  |
| $g\left(0\right)$ | $0$ |  |  |  |
| $\frac{\text{d}g\left(\text{t}\right)}{\text{d}t}$ | $ab\text{e}^{-bt}$ |  |  |  |
| $t_{\text{inf}}$ | N.M. |  |  |  |
| $g\left(t_{\text{inf}}\right)$ | N.M. |  |  |  |
| $\frac{\text{d}g\left(t_{\text{inf}}\right)}{\text{d}t}$ | N.M. |  |  |  |
| Negative Exponential 2b  Kind: increasing | | | | |
| Function | Equation | Constraints | | |
| $g\left(t\right)$ | $a\left(1-b^{t}\right)$ | $a>0$ | $0<b<1$ |  |
| $h\left(y\right)$ | $\frac{\text{Log}\left[-\frac{-a+y}{a}\right]}{\text{Log}\left[b\right]}$ | $a>y$ | $0<b<1$ |  |
| $g\left(0\right)$ | $0$ |  |  |  |
| $\frac{\text{d}g\left(\text{t}\right)}{\text{d}t}$ | $-ab^{t}\text{Log}\left[b\right]$ |  |  |  |
| $t_{\text{inf}}$ | N.M. |  |  |  |
| $g\left(t_{\text{inf}}\right)$ | N.M. |  |  |  |
| $\frac{\text{d}g\left(t_{\text{inf}}\right)}{\text{d}t}$ | N.M. |  |  |  |

| Negative Exponential 3a  Kind: increasing | | | | |
| --- | --- | --- | --- | --- |
| Function | Equation | Constraints | | |
| $g\left(t\right)$ | $a\left(1-\text{e}^{-b\left(-c+t\right)}\right)$ | $a>0$ | $b>0$ |  |
| $h\left(y\right)$ | $\frac{bc-\text{Log}\left[-\frac{-a+y}{a}\right]}{b}$ | $a>y$ | $b>0$ |  |
| $g\left(0\right)$ | $a-a\text{e}^{bc}$ |  |  |  |
| $\frac{\text{d}g\left(\text{t}\right)}{\text{d}t}$ | $ab\text{e}^{b\left(c-t\right)}$ |  |  |  |
| $t_{\text{inf}}$ | N.M. |  |  |  |
| $g\left(t_{\text{inf}}\right)$ | N.M. |  |  |  |
| $\frac{\text{d}g\left(t_{\text{inf}}\right)}{\text{d}t}$ | N.M. |  |  |  |

| Negative Exponential 3b  Kind: increasing | | | | |
| --- | --- | --- | --- | --- |
| Function | Equation | Constraints | | |
| $g\left(t\right)$ | $a-bc^{t}$ | $a>0$ | $b>0$ | $1>c>0$ |
| $h\left(y\right)$ | $\frac{\text{Log}\left[-\frac{-a+y}{b}\right]}{\text{Log}\left[c\right]}$ | $a>y$ | $b>0$ | $1>c>0$ |
| $g\left(0\right)$ | $a-b$ |  |  |  |
| $\frac{\text{d}g\left(\text{t}\right)}{\text{d}t}$ | $-bc^{t}\text{Log}\left[c\right]$ |  |  |  |
| $t_{\text{inf}}$ | N.M. |  |  |  |
| $g\left(t_{\text{inf}}\right)$ | N.M. |  |  |  |
| $\frac{\text{d}g\left(t_{\text{inf}}\right)}{\text{d}t}$ | N.M. |  |  |  |

| Negative Exponential 4a  Kind: increasing | | | | | | | |
| --- | --- | --- | --- | --- | --- | --- | --- |
| Function | Equation | Constraints | | | | | |
| $g\left(t\right)$ | $d+\left(a-d\right)\left(1-\text{e}^{-b\left(-c+t\right)}\right)$ | $a>0$ | $b>0$ | | $d<a$ | |  |
| $h\left(y\right)$ | $\frac{bc-\text{Log}\left[\frac{a-y}{a-d}\right]}{b}$ | $a>y$ | $b>0$ | | $d<a$ | |  |
| $g\left(0\right)$ | $a+\left(-a+d\right)\text{e}^{bc}$ |  | |  | |  | |
| $\frac{\text{d}g\left(\text{t}\right)}{\text{d}t}$ | $b\left(a-d\right)\text{e}^{b\left(c-t\right)}$ |  | |  | |  | |
| $t_{\text{inf}}$ | N.M. |  | |  | |  | |
| $g\left(t_{\text{inf}}\right)$ | N.M. |  | |  | |  | |
| $\frac{\text{d}g\left(t_{\text{inf}}\right)}{\text{d}t}$ | N.M. |  | |  | |  | |

| \| Rational 2z  Kind: asymptotic attenuating \| \| \| \| \| \| --- \| --- \| --- \| --- \| --- \| \| Function \| Equation \| Constraints \| \| \| \| $g\left(t\right)$ \| $\frac{abt}{1+bt}$ \| $a>0$ \| $b>0$ \|  \| \| $h\left(y\right)$ \| $-\frac{y}{b\left(-a+y\right)}$ \| $a>y$ \| $b>0$ \|  \| \| $g\left(0\right)$ \| 0 \|  \|  \|  \| \| $\frac{\text{d}g\left(\text{t}\right)}{\text{d}t}$ \| $\frac{ab}{\left(1+bt\right)^{2}}$ \|  \|  \|  \| \| $t_{\text{inf}}$ \| N.M. \|  \|  \|  \| \| $g\left(t_{\text{inf}}\right)$ \| N.M. \|  \|  \|  \| \| $\frac{\text{d}g\left(t_{\text{inf}}\right)}{\text{d}t}$ \| N.M. \|  \|  \|  \|   Rational 3a  Kind: asymptotic attenuating | | | | |
| --- | --- | --- | --- | --- | --- | --- | --- | --- | --- | --- | --- | --- | --- | --- | --- | --- | --- | --- | --- | --- | --- | --- | --- | --- | --- | --- | --- | --- | --- | --- | --- | --- | --- | --- | --- | --- | --- | --- | --- | --- | --- | --- | --- | --- | --- | --- | --- | --- | --- |
| Function | Equation | Constraints | | |
| $g\left(t\right)$ | $\frac{c+abt}{1+bt}$ | $a>0$ | $b>0$ | $a>c$ |
| $h\left(y\right)$ | $\frac{c-y}{b\left(-a+y\right)}$ | $a>y$ | $b>0$ | $a>c$ |
| $g\left(0\right)$ | $c$ |  |  |  |
| $\frac{\text{d}g\left(\text{t}\right)}{\text{d}t}$ | $\frac{b\left(a-c\right)}{\left(1+bt\right)^{2}}$ |  |  |  |
| $t_{\text{inf}}$ | N.M. |  |  |  |
| $g\left(t_{\text{inf}}\right)$ | N.M. |  |  |  |
| $\frac{\text{d}g\left(t_{\text{inf}}\right)}{\text{d}t}$ | N.M. |  |  |  |

| Rational 4  Kind: asymptotic attenuating | | | | | | | |
| --- | --- | --- | --- | --- | --- | --- | --- |
| Function | Equation | Constraints | | | | | |
| $g\left(t\right)$ | $d+\frac{c+b\left(a-d\right)t}{1+bt}$ | $a>0$ | $b>0$ | | $a>c$ | | $a>d$ |
| $h\left(y\right)$ | $\frac{c+d-y}{b\left(-a+y\right)}$ | $a>y$ | $b>0$ | | $a>c$ | | $a>d$ |
| $g\left(0\right)$ | $c+d$ |  | |  | |  | |
| $\frac{\text{d}g\left(\text{t}\right)}{\text{d}t}$ | $\frac{b\left(a-c-d\right)}{\left(1+bt\right)^{2}}$ |  | |  | |  | |
| $t_{\text{inf}}$ | N.M. |  | |  | |  | |
| $g\left(t_{\text{inf}}\right)$ | N.M. |  | |  | |  | |
| $\frac{\text{d}g\left(t_{\text{inf}}\right)}{\text{d}t}$ | N.M. |  | |  | |  | |

| Linear 2  Kind: increasing | | | | |
| --- | --- | --- | --- | --- |
| Function | Equation | Constraints | | |
| $g\left(t\right)$ | $b+at$ | $a>0$ |  |  |
| $h\left(y\right)$ | $\frac{-b+y}{a}$ | $a>0$ |  |  |
| $g\left(0\right)$ | $b$ |  |  |  |
| $\frac{\text{d}g\left(\text{t}\right)}{\text{d}t}$ | $a$ |  |  |  |
| $t_{\text{inf}}$ | N.M. |  |  |  |
| $g\left(t_{\text{inf}}\right)$ | N.M. |  |  |  |
| $\frac{\text{d}g\left(t_{\text{inf}}\right)}{\text{d}t}$ | N.M. |  |  |  |

| Exponential 2  Kind: increasing | | | | |
| --- | --- | --- | --- | --- |
| Function | Equation | Constraints | | |
| $g\left(t\right)$ | $a\text{e}^{bt}$ | $a>0$ | $b>0$ |  |
| $h\left(y\right)$ | $\frac{\text{Log}\left[\frac{y}{a}\right]}{b}$ | $a>0$ | $b>0$ |  |
| $g\left(0\right)$ | $a$ |  |  |  |
| $\frac{\text{d}g\left(\text{t}\right)}{\text{d}t}$ | $ab\text{e}^{bt}$ |  |  |  |
| $t_{\text{inf}}$ | N.M. |  |  |  |
| $g\left(t_{\text{inf}}\right)$ | N.M. |  |  |  |
| $\frac{\text{d}g\left(t_{\text{inf}}\right)}{\text{d}t}$ | N.M. |  |  |  |

| Exponential 3  Kind: increasing | | | | |
| --- | --- | --- | --- | --- |
| Function | Equation | Constraints | | |
| $g\left(t\right)$ | $c+a\text{e}^{bt}$ |  |  |  |
| $h\left(y\right)$ | $\frac{\text{Log}\left[\frac{-c+y}{a}\right]}{b}$ | $\frac{-c+y}{a}>0$ | | |
| $g\left(0\right)$ | $c+a$ |  |  |  |
| $\frac{\text{d}g\left(\text{t}\right)}{\text{d}t}$ | $ab\text{e}^{bt}$ |  |  |  |
| $t_{\text{inf}}$ | N.M. |  |  |  |
| $g\left(t_{\text{inf}}\right)$ | N.M. |  |  |  |
| $\frac{\text{d}g\left(t_{\text{inf}}\right)}{\text{d}t}$ | N.M. |  |  |  |

| Quadratic 2  Kind: increasing | | | | |
| --- | --- | --- | --- | --- |
| Function | Equation | Constraints | | |
| $g\left(t\right)$ | $b+at^{2}$ | $a>0$ |  |  |
| $h\left(y\right)$ | $\frac{\sqrt{-b+y}}{\sqrt{a}}$ | $a>0$ | $y>b$ |  |
| $g\left(0\right)$ | $b$ |  |  |  |
| $\frac{\text{d}g\left(\text{t}\right)}{\text{d}t}$ | $2at$ |  |  |  |
| $t_{\text{inf}}$ | N.M. |  |  |  |
| $g\left(t_{\text{inf}}\right)$ | N.M. |  |  |  |
| $\frac{\text{d}g\left(t_{\text{inf}}\right)}{\text{d}t}$ | N.M. |  |  |  |

| Quadratic 2b  Kind: increasing | | | | |
| --- | --- | --- | --- | --- |
| Function | Equation | Constraints | | |
| $g\left(t\right)$ | $\left(b+at\right)^{2}$ |  |  |  |
| $h\left(y\right)$ | $\frac{-b+\sqrt{y}}{a}$ , $-\frac{b+\sqrt{y}}{a}$ | $a>0$ |  |  |
| $g\left(0\right)$ | $b^{2}$ |  |  |  |
| $\frac{\text{d}g\left(\text{t}\right)}{\text{d}t}$ | $2a\left(b+at\right)$ |  |  |  |
| $t_{\text{inf}}$ | N.M. |  |  |  |
| $g\left(t_{\text{inf}}\right)$ | N.M. |  |  |  |
| $\frac{\text{d}g\left(t_{\text{inf}}\right)}{\text{d}t}$ | N.M. |  |  |  |

| Quadratic 3  Kind: increasing | | | | |
| --- | --- | --- | --- | --- |
| Function | Equation | Constraints | | |
| $g\left(t\right)$ | $c+bt+at^{2}$ |  |  |  |
| $h\left(y\right)$ | $\frac{-b\pm\sqrt{b^{2}-4a\left(c-y\right)}}{2a}$ , $\frac{-c+y}{b}$ | $b^{2}-4a\left(c-y\right)\geq0$ | | |
| $g\left(0\right)$ | $c$ |  |  |  |
| $\frac{\text{d}g\left(\text{t}\right)}{\text{d}t}$ | $b+2at$ |  |  |  |
| $t_{\text{inf}}$ | N.M. |  |  |  |
| $g\left(t_{\text{inf}}\right)$ | N.M. |  |  |  |
| $\frac{\text{d}g\left(t_{\text{inf}}\right)}{\text{d}t}$ | N.M. |  |  |  |

| Cubic 2  Kind: increasing | | | | |
| --- | --- | --- | --- | --- |
| Function | Equation | Constraints | | |
| $g\left(t\right)$ | $b+at^{3}$ | $a>0$ |  |  |
| $h\left(y\right)$ | $\frac{\left(-b+y\right)^{1/3}}{a^{1/3}}$ | $a>0$ |  |  |
| $g\left(0\right)$ | $b$ |  |  |  |
| $\frac{\text{d}g\left(\text{t}\right)}{\text{d}t}$ | $3at^{2}$ |  |  |  |
| $t_{\text{inf}}$ | N.M. |  |  |  |
| $g\left(t_{\text{inf}}\right)$ | N.M. |  |  |  |
| $\frac{\text{d}g\left(t_{\text{inf}}\right)}{\text{d}t}$ | N.M. |  |  |  |

| Cubic 2b  Kind: increasing | | | | |
| --- | --- | --- | --- | --- |
| Function | Equation | Constraints | | |
| $g\left(t\right)$ | $\left(b+at\right)^{3}$ | $a>0$ |  |  |
| $h\left(y\right)$ | $\frac{-b+y^{1/3}}{a}$ | $a>0$ |  |  |
| $g\left(0\right)$ | $b^{3}$ |  |  |  |
| $\frac{\text{d}g\left(\text{t}\right)}{\text{d}t}$ | $3a\left(b+at\right)^{2}$ |  |  |  |
| $t_{\text{inf}}$ | N.M. |  |  |  |
| $g\left(t_{\text{inf}}\right)$ | N.M. |  |  |  |
| $\frac{\text{d}g\left(t_{\text{inf}}\right)}{\text{d}t}$ | N.M. |  |  |  |

| Power 2  Kind: increasing | | | | |
| --- | --- | --- | --- | --- |
| Function | Equation | Constraints | | |
| $g\left(t\right)$ | $at^{b}$ | $a>0$ | $b>0$ |  |
| $h\left(y\right)$ | $\left(\frac{y}{a}\right)^{\frac{1}{b}}$ | $a>0$ | $b>0$ |  |
| $g\left(0\right)$ | 0 |  |  |  |
| $\frac{\text{d}g\left(\text{t}\right)}{\text{d}t}$ | $abt^{-1+b}$ |  |  |  |
| $t_{\text{inf}}$ | N.M. |  |  |  |
| $g\left(t_{\text{inf}}\right)$ | N.M. |  |  |  |
| $\frac{\text{d}g\left(t_{\text{inf}}\right)}{\text{d}t}$ | N.M. |  |  |  |

| Power 3  Kind: increasing | | | | |
| --- | --- | --- | --- | --- |
| Function | Equation | Constraints | | |
| $g\left(t\right)$ | $c+at^{b}$ | $a>0$ | | |
| $h\left(y\right)$ | $\left(\frac{-c+y}{a}\right)^{\frac{1}{b}}$ | $\begin{matrix} a>0 & b>0 & c<y \end{matrix}$ | | |
| $g\left(0\right)$ | $c$ |  |  |  |
| $\frac{\text{d}g\left(\text{t}\right)}{\text{d}t}$ | $abt^{-1+b}$ |  |  |  |
| $t_{\text{inf}}$ | N.M. |  |  |  |
| $g\left(t_{\text{inf}}\right)$ | N.M. |  |  |  |
| $\frac{\text{d}g\left(t_{\text{inf}}\right)}{\text{d}t}$ | N.M. |  |  |  |

| Power 3b  Kind: increasing | | | | | | |
| --- | --- | --- | --- | --- | --- | --- |
| Function | Equation | Constraints | | | | |
| $g\left(t\right)$ | $\left(b+at\right)^{c}$ | $b+at>0$ | | | | |
| $h\left(y\right)$ | $\frac{-b+y^{\frac{1}{c}}}{a}$ |  | $a>0$ | |  | |
| $g\left(0\right)$ | $b^{c}$ |  | |  | |  |
| $\frac{\text{d}g\left(\text{t}\right)}{\text{d}t}$ | $ac\left(b+at\right)^{-1+c}$ |  | |  | |  |
| $t_{\text{inf}}$ | N.M. |  | |  | |  |
| $g\left(t_{\text{inf}}\right)$ | N.M. |  | |  | |  |
| $\frac{\text{d}g\left(t_{\text{inf}}\right)}{\text{d}t}$ | N.M. |  | |  | |  |

| Persistence 3a  Kind: increasing | | | | | |
| --- | --- | --- | --- | --- | --- |
| Function | Equation | Constraints | | | |
| $g\left(t\right)$ | $a\text{e}^{-ct}t^{b}$ | $a>0$ | | | |
| $h\left(y\right)$ | $-\frac{b\text{ΠLog}\left[-\frac{c\left(\frac{y}{a}\right)^{\frac{1}{b}}}{b}\right]}{c}$ , $-\frac{b\text{ΠLog}2\left[-\frac{c\left(\frac{y}{a}\right)^{\frac{1}{b}}}{b}\right]}{c}$ | $a>0$ | | $b>c\text{e}\left(\frac{y}{a}\right)^{\frac{1}{b}}$ | |
| $g\left(0\right)$ | 0 |  |  | |  |
| $\frac{\text{d}g\left(\text{t}\right)}{\text{d}t}$ | $a\text{e}^{-ct}t^{-1+b}\left(b-ct\right)$ |  |  | |  |
| $t_{\text{inf}}$ | N.M. |  |  | |  |
| $g\left(t_{\text{inf}}\right)$ | N.M. |  |  | |  |
| $\frac{\text{d}g\left(t_{\text{inf}}\right)}{\text{d}t}$ | N.M. |  |  | |  |

| Persistence 3b  Kind: increasing | | | | |
| --- | --- | --- | --- | --- |
| Function | Equation | Constraints | | |
| $g\left(t\right)$ | $a\text{e}^{-\frac{c}{t}}t^{b}$ | $a>0$ | | |
| $h\left(y\right)$ | $\frac{c}{b\text{ΠLog}\left[-\frac{c\left(\frac{y}{a}\right)^{\frac{1}{b}}}{b}\right]}$ , $\frac{c}{b\text{ΠLog}2\left[-\frac{c\left(\frac{y}{a}\right)^{\frac{1}{b}}}{b}\right]}$ | $a>0$ | $b\neq0$ | $\frac{c\left(\frac{y}{a}\right)^{-\frac{1}{b}}}{b}>-\frac{1}{e}$ |
| $g\left(0\right)$ | 0 |  |  |  |
| $\frac{\text{d}g\left(\text{t}\right)}{\text{d}t}$ | $a\text{e}^{-\frac{c}{t}}t^{-2+b}\left(c+bt\right)$ |  |  |  |
| $t_{\text{inf}}$ | N.M. |  |  |  |
| $g\left(t_{\text{inf}}\right)$ | N.M. |  |  |  |
| $\frac{\text{d}g\left(t_{\text{inf}}\right)}{\text{d}t}$ | N.M. |  |  |  |

| Persistence 4a  Kind: increasing | | | | |
| --- | --- | --- | --- | --- |
| Function | Equation | Constraints | | |
| $g\left(t\right)$ | $at^{b}\text{e}^{-ct}+d$ |  |  |  |
| $h\left(y\right)$ | $-\frac{b\text{ProductLog}\left[-\frac{c\left(\frac{y-d}{a}\right)^{\frac{1}{b}}}{b}\right]}{c}$ , $-\frac{b\text{ProductLog}2\left[-\frac{c\left(\frac{y-d}{a}\right)^{\frac{1}{b}}}{b}\right]}{c}$ | $a>0$ | $y>d$ | $b>c\text{e}\left(\frac{y-d}{a}\right)^{\frac{1}{b}}$ |
| $g\left(0\right)$ | 0 |  |  |  |
| $\frac{\text{d}g\left(\text{t}\right)}{\text{d}t}$ | $ab\text{e}^{-ct}t^{-1+b}-ac\text{e}^{-ct}t^{b}$ |  |  |  |
| $t_{\text{inf}}$ | N.M. |  |  |  |
| $g\left(t_{\text{inf}}\right)$ | N.M. |  |  |  |
| $\frac{\text{d}g\left(t_{\text{inf}}\right)}{\text{d}t}$ | N.M. |  |  |  |

| Persistence 4b  Kind: increasing | | | | |
| --- | --- | --- | --- | --- |
| Function | Equation | Constraints | | |
| $g\left(t\right)$ | $at^{b}\text{e}^{\frac{-c}{t}}+d$ |  |  |  |
| $h\left(y\right)$ | $\frac{c}{b\text{ProductLog}\left[\frac{c\left(\frac{y-d}{a}\right)^{-1/b}}{b}\right]}$ , $\frac{c}{b\text{ProductLog}2\left[\frac{c\left(\frac{y-d}{a}\right)^{-1/b}}{b}\right]}$ | $a>0$ | $b\neq0$ | $y>d$ |
|  |  | $\frac{c\left(\frac{y-d}{a}\right)^{-1/b}}{b}>-1/e$ | | |
| $g\left(0\right)$ | 0 |  |  |  |
| $\frac{\text{d}g\left(\text{t}\right)}{\text{d}t}$ | $ac\text{e}^{-\frac{c}{t}}t^{-2+b}+ab\text{e}^{-\frac{c}{t}}t^{-1+b}$ |  |  |  |
| $t_{\text{inf}}$ | N.M. |  |  |  |
| $g\left(t_{\text{inf}}\right)$ | N.M. |  |  |  |
| $\frac{\text{d}g\left(t_{\text{inf}}\right)}{\text{d}t}$ | N.M. |  |  |  |

**References for Table S1**

1. Mielke PW, Johnson ES (1974) Some generalized beta distributions of the second kind having desirable application features in hydrology and meteorology. Water Resources Research 10: 223–226.

2. Tjørve E (2003) Shapes and functions of species-area curves: a review of possible models. Journal of Biogeography 30: 827–835.

3. Lynch HJ, Fagan WF (2009) Survivorship curves and their impact on the estimation of maximum population growth rates. Ecology 90: 1116–1124.

4. Ratkowsky DA (1990) Handbook of nonlinear regression models. New York, N.Y.: Marcel Dekker.

5. Birch CPD (1999) A new generalized logistic sigmoid growth equation compared with the Richards growth equation. Annals of Botany 83: 713–723.

6. Fekedulegn D, Siurtain MPM, Colbert JJ (1999) Parameter estimation of nonlinear growth models in forestry. Silva Fennica 33: 327–336.

7. Tjørve KMC, Tjørve E (2010) Shapes and functions of bird-growth models: how to characterise chick postnatal growth. Zoology 113: 326–333. doi:10.1016/j.zool.2010.05.003.

8. Williams MR (1995) An extreme-value function model of the species incidence and species-area relations. Ecology 76: 2607–2616.

9. Winsor CP (1932) The Gompertz Curve as a Growth Curve. Proceedings of the National Academy of Sciences 18.

10. Verhagen AMW (1960) Growth curves and their functional form. Australian and New Zealand Journal of Statistics 2: 122–127.

11. Franses PH (1994) Fitting a Gompertz curve. The Journal of the Operational Research Society 45: 109–113. doi:10.1038/sj/jors/0450111.

12. He F, Legendre P (2002) Species diversity patterns derived from species-area models. Ecology 83: 1185–1198.

13. He F, Legendre P (1996) On species-area relations. American Naturalist 148: 719–737.

14. Zeide B (1993) Analysis of growth equations. Forest Science 39: 594–616.

15. Wilson DL (1994) The analysis of survival (mortality) data: fitting Gompertz, Weibull, and logistic functions. Mechanisms of Ageing and Development 74: 15–33.

16. Verhulst PF (1838) Notice sur la loi que la population suit dans son accroisement. Mathématiques et Physiques 10: 113–121.

17. Tsoularis A, Wallace J (2002) Analysis of logistic growth models. Mathematical Biosciences 179: 21–55.

18. Lomolino MV (1985) Body size of mammals on islands: the island rule reexamined. The American Naturalist 125: 310–316.

19. Morgan PH, Mercer LP, Flodin NW (1975) General model for nutritional responses of higher organisms. Proceedings of the National Academy of Sciences 72: 4327–4331.

20. López S, Prieto M, Dijkstra J, Dhanoa MS, France J (2004) Statistical evaluation of mathematical models for microbial growth. International Journal of Food Microbiology 96: 289–300. doi:10.1016/j.ijfoodmicro.2004.03.026.

21. Carrillo M, González JM (2002) A new approach to modelling sigmoidal curves. Technological Forecasting and Social Change 69: 233–241. doi:10.1016/S0040-1625(01)00150-0.

22. Richards FJ (1959) A flexible growth function for empirical use. Journal of Experimental Botany 10: 290–301. doi:10.1093/jxb/10.2.290.

23. Leberg PL, Brisbin, Jr. IL, Smith MH, White GC (1989) Factors affecting the analysis of growth patterns of large mammals. Journal of Mammalogy 70: 275–283.

24. Tjørve E, Tjørve KMC (2010) A unified approach to the Richards-model family for use in growth analyses: why we need only two model forms. Journal of Theoretical Biology 267: 417–425. doi:10.1016/j.jtbi.2010.09.008.

25. Bertalanffy L von (1957) Quantitative laws in metabolism and growth. The Quarterly Review of Biology 32: 217–231.

26. Zullinger EM, Ricklefs RE, Redford KH, Mace GM (1984) Fitting Sigmoidal Equations to Mammalian Growth Curves. Journal of Mammalogy 65: 607–636.

27. Weibull W (1951) A statistical distribution function of wide applicability. Journal of Applied Mechanics 18: 293–297.

28. Yang RC, Kozak A, Smith JHG (1978) The potential of Weibull-type functions as flexible growth curves. Canadian Journal of Forest Research 8: 424–431.

29. López S, France J, Gerrits WJ, Dhanoa MS, Humphries DJ, et al. (2000) A generalized Michaelis-Menten equation for the analysis of growth. Journal of Animal Science 78: 1816–1828.
