# Supplementary material for: Revisiting the Estimation of Dinosaur Growth Rates
Source: PLoS One. 2013 Dec 16;8(12):e81917. doi: 10.1371/journal.pone.0081917 (PMC3864909; doi:10.1371/journal.pone.0081917)
Supplement: Text S1 — Attempting to replicate prior results. (DOCX) [file pone.0081917.s023.docx]

**Text S1. Attempting to replicate prior results**

Several prior dinosaur studies publish a set of age-mass data points, or age-bone dimension data points, and they report the regression equation resulting from least squares regression curve fit to those data points. In principle, it should be easy to replicate the fit by using standard nonlinear regression techniques to fit a curve with the same functional form to the published data set. This should yield the same parameter values as the best fit parameters published in the original paper.

In the course of comparing results of this paper to prior work, it became obvious that some of the published regression equations did not match the best fits to the data. Similarly, one would expect that properties derived from the published regression equation, such as the maximum growth rate (maximum value of the first time derivative of the growth curve), should also be easy to replicate. This also was problematic in many cases.

Finally, one would expect that published figures plot the data sets and regression equations called for in their captions. This also turned out to be an issue in some cases.

For the purpose of this paper replicating a result means that I was able to calculate it from the available data, with the method of analysis described in the original study. A failure to replicate means that my attempt at replication produced numerical values that were significantly different than those presented in the original study, either at the numerical precision originally reported, or via some other metric.

In some cases I also attempted methods of analysis corresponding to possible interpretations of the original study methodology, where there was an ambiguity. As described below, in some cases I also tried to anticipate possible typographical errors in regression equations.

When a study discloses least squares regression as the method of curve fitting, normal statistical practice sets the expectation that all of the numerical parameter values in the resulting regression equations were found by the least-squares regression procedure, strictly as a function of the data points.

One explicitly does not expect that any parameters were set other than by a least squares regression algorithm. If some of the parameter values were determined by another procedure, it might explain why they could not be replicated using least squares regression. In several cases there is some ambiguity in the disclosed method, either because of uncertainty in the model used for regression, or whether some other procedure was used. In those cases I have attempted several variations to account for the ambiguity.

**Models to Fit**

In order to replicate the published results, one should fit the same growth function models. The task in replicating a result is not to question whether the choice of model (say logistic or von Bertalanffy) is the best choice, but simply to confirm that one can get the same parameter values for the same functions.

Note that the mathematical definitions of the growth functions in the original papers may differ from the functions of the same name (e.g. logistic) used elsewhere in this paper (for example, Table S1.)

In the case of *Tyrannosaurus, Gorgosaurus, Albertosaurus, Psittacosaurus mongoliensis, Massospondylus,* and *Syntarsus*, the reported regression equations in the original paper [20,25], are four-parameter logistic functions, which I will refer to here as fit function A:

$$m_{\text{A}}\left(t\right)= \frac{a }{1- e^{-b\left(t-c\right)}}+d$$

Note however that references [20,25] do not explicitly give the logistic equation in symbolic form such as $m_{\text{A}}\left(t\right)$ above. Instead they report the final regression equations with numerical values inserted. For example, the *Tyrannosaurus* regression function is given in [25] as

$$mass= \frac{5551 }{1- e^{-0.57\left(age -16.1\right)}}+5$$

Mathematically this is equivalent to $m_{\text{A}}\left(t\right)$ with age identified with $t$ and with parameter values $a=5551, b=0.57, c=16.1, d=5$. However in reference [25], each of the reported regression equations has $d=5$; in contrast in reference [20], where the reported fit to each taxon has different values for $d$.

The parameter $d$ has particular influence on the value of the growth curve for neonates. Using the reported regression equations from reference [25], the neonate (age $t=0$) masses are 5.57 kg for *Tyrannosaurus*, 15.99 kg for *Gorgosaurus* and 7.82 kg for *Albertosaurus*. It is unclear why the smaller taxa would have much larger neonatal masses than for *Tyrannosaurus*, or why *Gorgosaurus* has a mass that is over twice that of *Albertorsaurus*, even though the known adult specimens are about the same size. Unfortunately neonate mass is poorly constrained by fossil data. No hatchlings for large tyrannosaurs have been discovered, nor have any eggs definitively been identified with those taxa. Large fossil eggs classified as *Macroelongatoolithus xixiaensis* have been suggested as being laid by large theropods [119], but the identification of their associated egg layer is very tentative.

The coincidence that $d=5$ for all of the taxa in [25] suggests a possible ambiguity; perhaps the regression was not performed with function A and instead was based on function B.

$$m_{\text{B}}\left(t\right)= \frac{a }{1- e^{-b\left(t-c\right)}}+5$$

To account for this possibility, I also tried replicating the regression results using function B. Note that function B would not be a possibility for reference [20] which has different values of the parameter $d$ for each taxon.

I also tried versions $\text{A}_{2} \text{and} \text{B}_{2}$ which are functions A and B, respectively, both with parameter $a$ fixed to the same value, *a*_pub_, as the regression equations published in the original study for the taxon in question.

$$m_{\text{A}_{2}}\left(t\right)= \frac{a_{\text{pub}} }{1- e^{-b\left(t-c\right)}}+d$$

$$m_{\text{B}_{2}}\left(t\right)= \frac{a_{\text{pub}} }{1- e^{-b\left(t-c\right)}}+5$$

Given the difficulty I had in replicating the results with other choices I thought that it might be informative to try a fit with the value of $a$ set to the same value published in the original studies.

In the case of *Psittacosaurus mongoliensis*, the regression equation reported in reference [24], is a three-parameter logistic function of the form:

$$m_{\text{C}}\left(t\right)= \frac{a }{1- e^{-b\left(t-c\right)}}$$

I also tried a version, C_2_, which is function C with $a= a_{\text{pub}}$, where $a_{\text{pub}}$ is the published value for the regression in the original paper for the taxon in question.

$$m_{\text{C}_{2}}\left(t\right)= \frac{a_{\text{pub}} }{1- e^{-b\left(t-c\right)}},$$

The fit to *P. lujiatunensis* was specified in the original paper [18] as

$$m_{\text{specified}}\left(t\right)= \frac{37.38 e^{0.55 t}}{37.38+ e^{0.55 t}-1}$$

So the fit was made using the two parameter logistic function, which will be referred to as function D.

$$m_{\text{D}}\left(t\right)= \frac{a e^{b t}}{a+ e^{b t}-1}$$

Given the difficulty that I had in replicating the fit, I also considered the possibility that a typographical error might have been made, and the equation specified in the paper might actually be

$$\frac{37.38 e^{0.55 t}}{37.38+ e^{0.55 t}}-1$$

In order to consider that possibility, and other possible typographical errors, the logistic functions E and F and G were also fit to the data.

$$m_{\text{E}}\left(t\right)= \frac{a e^{b t}}{a+ e^{b t}}+c, m_{\text{F}}\left(t\right)= \frac{a e^{b t}}{a+ e^{b t}}-1, m_{\text{G}}\left(t\right)= \frac{a e^{b t}}{a-c + e^{b t}}$$

I also considered a four-parameter logistic function, function A.

Note that function $m_{\text{D}}\left(t\right)$ is the closest functional form to the reported equations in [18], but an argument could also be made for function $m_{\text{G}}\left(t\right)$ with $c=1$.

I also tried versions of these functions with the value of $a= a_{\text{pub}}$, where $a_{\text{pub}}= 37.38$ is the published value for the regression in the original paper for *P. lujiatunensis.* This gives a series of functions $\text{A}_{2}, \text{C}_{2}, \text{D}_{2}, \text{E}_{2}, \text{F}_{2}, \text{ and G}_{2}$ which have $a= a_{\text{pub}}$. The functions $\text{A}_{2}, \text{ and C}_{2}$ appear above, the others are as follows.

$$m_{\text{D}_{2}}\left(t\right)= \frac{37.38 e^{b t}}{37.38+ e^{b t}-1},\text{ }m_{\text{E}_{2}}\left(t\right)= \frac{37.38 e^{b t}}{37.38+ e^{b t}}+c$$

$$m_{\text{F}_{2}}\left(t\right)= \frac{37.38 e^{b t}}{37.38+ e^{b t}}-1, m_{\text{G}_{2}}\left(t\right)= \frac{37.38 e^{b t}}{37.38-c + e^{b t}}$$

Again, I have no specific information to suggest that the original study did set $a= a_{\text{pub}}$, but in order to explore all possibilities I tried these functions.

Fits to *Alamosaurus*, *Apatosaurus, Janenschia* and the Northampton sauropod were made using the three parameter von Bertalanffy function specified in the original paper [37], which will be function H.

$$m_{\text{H}}\left(t\right)= a\left(1-e^{-b\left(t-c\right)}\right)^{3}$$

**Data Sets**

In the case of some references, the age-mass data set used for model fitting is explicitly presented in the paper, but in other cases, there is some ambiguity regarding the data set.

In the case of *Massospondylus*, and *Syntarsus* the paper presenting growth rates [20] states that the underlying data was obtained from prior work by Chinsamy on *Syntarsus* [5] and *Massospondylus* [6]. Those papers discuss ages of specimens, but do not present either mass or bone dimension. Reference [20] explicitly references [5] and [6] for mass estimates as well as ages, but mass estimates do not appear in those papers, nor are they available now (or previously) from the original author (Chinsamy-Turan, Unpublished Data). Femur length data are available from the author (as I acquired more recently) (Chinsamy-Turan, Unpublished Data), and this presumably is where the investigators of reference [20] obtained the data previously. Since DME was used in estimating masses for other taxa in reference [20], it seemed appropriate to use it to determine masses from femur lengths.

The crucial missing datum to do this is the mass of the individual with the largest femur length. This is implicitly contained in the plotted figures of reference [20], so a high resolution digital scan was used with image processing software (Adobe Photoshop CS6) to measure the height of the largest data point from the x-axis, which was then scaled relative to similar measurements of the tick marks on the y-axis. Once this mass was determined, the rest of the masses could then be obtained by DME scaling based on the published femur-length age pairs. Measuring the mass from the plots in this fashion could introduce a small error in the age-mass pairs used for these taxa, but I estimate that it is much less than$1\%$, because the pixel count of the digital scan was high (500 – 1100 pixels for the measured y-values), and I estimate the positional errors to be plus or minus one pixel.

Note however, that this assumes that the plots were created by plotting software that accurately represents that data points. If the plots were not created by plotting software, or were edited after production by a graphics program (for example, Adobe Illustrator), then that may potentially alter the scale or introduce nonlinearities. As will be discussed in more detail below, subsequent work with the plots shows that this does not appear to be problematic, at least with respect to determining the scaling mass for DME.

As an additional check, I used image processing algorithms in commercial mathematical software (Wolfram Research, Mathematica 9.01) to recover the data points as plotted from the plots in reference [20], as will be discussed below.

In the case of *Psittacosaurus lujiatunensis,* the growth rate study [18] contains age-femur length data, but omits mass data. A data set of 80 data points in Table 1 of Reference [18], as age-femur length pairs. The paper specifies that these were converted to age-mass pairs by DME scaling, based on the specimen with 201 mm femur length (LPM R00117). Unfortunately the mass of LPM R00117 was not published, but I was able to obtain its mass using a digital scan of Figure 6 of reference [18] in the manner discussed above. This mass was then used to scale to other masses DME.

The most straightforward interpretation of the data set to be used for regression analysis would be the full data set of 80 points from Table 1, scaled via DME from the femur length and mass of LPM R00117. This is the basis for the data set *Psittacosaurus l*1. However there are several other possible ambiguities that led me to consider alternative data sets.

Some of the specimens (26 data points) in Table 1 of reference [18] had ages determined histologically (i.e. by counting LAGs), others were assigned ages using linear regression with femur length as the independent variable. Reference [18] is silent on whether this distinction was used to choose a subset of Table 1 for regression, but since that is a possibility, I also constructed *Psittacosaurus l*3 data set that only contains the 26 histologically aged specimens.

Close examination of Figure 6 of reference [18], as will be discussed in detail below, indicated that the plotted data is neither *Psittacosaurus l*1, nor *Psittacosaurus l*3*.* Using image processing software (Wolfram Research, Mathematica 9.01) the data points, as-plotted, were recovered as *Psittacosaurus l*5.

One final issue with the data must be addressed; the issue of multiple identical data points. The data set as published in Table 1 has multiple specimens with the same age and size – in one case, there are 34 identically sized and aged specimens. It is not possible to tell from the original plot whether the data set used in [18] used for fitting included all of the data points, or only one point from each age-size class.

Philosophically, one can take different positions on how to treat the multiple data points. The argument to include all of the data is that it uses all of the data available to us. The argument to use only one data point to represent each age-size pair is that we will overweight the sizes that, by luck of the find, happen to occur in multiple copies. Since the multiple specimens appear to be nestlings in the same nest, they may have less diversity than the same number of specimens sampled from different nests and sites.

Larger specimens tend to come from different sites unless found in a bone bed or other multiple specimen assemblage. Although we are fortunate to have a specimen that has 34 identical hatchlings, including all of them will simply over-weight the curve fit to that lucky find rather than a true representation of what would have occurred in life.

Each position has merit, and ideally one would try both cases to understand the sensitivity to the choice. Since the original paper was ambiguous about this, I prepared two versions of each data – one with all of the points, the other with only one point for each distinct age-mass pair.

This gives us a total of 6 data sets. *P. lujiatunensis* 1 is the full data set, while *P. lujiatunensis* 2 is the version with only one point for each distinct age-mass pair. *P. lujiatunensis* 3 is the subset that was histologically aged, and *P. lujiatunensis* 4 is the version of the subset that was histologically aged with only one point for each distinct age-mass pair. *P. lujiatunensis* 5 is the recovered data set, made to conform to what appears to be plotted in Figure 6 of reference [18], and *P. lujiatunensis* 6 is the version of the recovered data set with only one point for each distinct age-mass pair.

The age-mass data points used in attempting to replicate the results are in Table S7.

**Fitting Methodology**

Nonlinear regression was used to fit the models and data, using the “NonlinearModelFit[ ]” function of Mathematica 9.01. Each fit was performed multiple times with different local and global minimization algorithms. Where different algorithms produced different fits, the best fit was chosen using the Akaike information criterion. As starting guesses for the fits, I used the parameters from the published regression equation. This should give a high likelihood of replicating the original result. Comparison of the results for the fits I obtained to the published results was done in three ways. First, one can directly compare the parameters of the best fit to the parameters of the published regression equations. This is the most direct comparison and deals directly with the issue of replication of the results. As discussed above there are several cases where alternative models were chosen, and it is not possible to directly compare parameters between different models. In those cases one can compare biological parameters like the maximum asymptotic size $g\left(\infty\right)$ or maximum growth rate . Third, the results were compared graphically by plotting the data points, the best fit that I found, and the reported regression equation. The attempt to replicate the result was done for the fits in references [18,20,24,25] and [37].

**Results from Attempted Replication**

In no case was a fit entirely the same as in the original paper to within the numerical precision reported in the original results. Some small differences are to be expected because of numerical precision issues that differ between computer systems or software implementations. However, one would expect these to be very small. The actual results did not fulfill this expectation, as most are different by more than $\pm10\%$. The full results appear in Table S4 A, which is a superset of Table 3 found in the main paper.

The process of calculating a best fit using nonlinear regression involves complicated numerical algorithms. It is possible that differences in the implementation of these algorithms may explain some of the differences, as will be discussed below. Fortunately, it is easy to verify which fit is best. Least squares regression seeks to find the fit for which the sum of the square of the errors is smallest. Calculating the sum of the square of the errors is a very simple calculation; the fit with the smallest value is best. Table S10 is a simple spreadsheet that does this calculation to show that the fits I found by attempted replication are in fact better fits than the originals.

Only two (*Albertosaurus* and *Psittacosaurus mongoliensis*) of the eight fits (25%) from the Erickson *et al.* papers [18,20,24,25] can be replicated to within $\pm10\%$ for the two biological parameters of interest. Many of the fits differ by 30% or more; in several cases by more than a factor of 10.

Even the taxa which can be replicated to within $\pm10\%$ are not perfect. The fit found for *Albertosaurus* using functions A and B is significantly different than the original published fits, even though it does replicate the particular parameters used here as a metric to within $\pm10\%$. The same is true for *Psittacosaurus mongoliensis*; the two papers that cover it [20,24] produce regression equations that differ from each other, and both differ from the best fits that I found (see Figure S1). However, when measured as deviation for the two biological factors found in Table S4 A, they are within $\pm10\%$. Even if we allow for differences in numerical tolerance this should be much tighter.

The situation for the curve fits from reference [37] is somewhat different. In that paper, Lehman and Woodward do not perform regression at all. They choose the parameter $a$ based on published maximum mass estimates from other papers. They then plot a two parameter von Bertalanffy function (i.e. the three parameter equation above with $c=0$), with several choices of the $b$ parameter varying starting age for their age-mass data. Varying the starting age is equivalent to choosing a parameter $c$ in the three-parameter von Bertalanffy function shown above. To attempt to replicate their fit, I used the three-parameter curve with values for $a$, $b$ and $c$ from their paper (for example, Table 2).

Note however that Lehman and Woodward do not promise best fits, so this is not really a failure to replicate their results. Indeed they explicitly say, “a high degree of precision in curve fitting is unwarranted”, citing uncertainties in mass estimation and other factors. This is why they present several different parameter scenarios, without calling them best fits. Several scenarios are chosen to bracket the data points from above or below rather than show a best fit. This is quite different than the approach taken in references [18,20,24,25], which explicitly present “best” fit results.

Given this, it is perhaps surprising that several of their fit scenarios for *Apatosaurus* and *Alamosaurus* have a very close correspondence with the best fits that I find. The plots that Lehman and Woodward make of their fits appear to be qualitatively similar to my plots (see Figure S3 and Tables 5, S4). The results for *Janenschia* and the Northampton sauropod do not closely match. Altogether, 3 out of 10, or 33% of the fits presented in reference [37] can be replicated to within $\pm10\%$, consistent with the original fits being designed primarily as upper, lower and midrange values – the midrange values are approximate best fits, the bounds are not.

**Possible Explanations**

It is difficult to explain my failure to replicate the published results. One possibility is a difference in the computational implementation of least squares regression when applied to a nonlinear curve such as logistic or others. Nonlinear regression seeks to minimize the square of the difference between the curve and the data points. The numerical minimization algorithms used to do this are notoriously prone to getting “stuck” on a local minimum and failing to find a global minimum.

Some numerical optimization algorithms contain features designed to detect and recover from a local minimum. If the algorithm used in the attempted replication were better able to escape from a local minimum than the algorithm used in the original papers, then the replicated analysis might find a better fit, while the original regression could have been stuck. This could conceivably be an explanation of the failure to replicate the results, at least in some cases.

Some of the fits appear to be ill-posed, in that they are fitting data which is substantially linear with a sigmoidal model. This is true, for example, of fits to *Gorgosaurus* growth data. By the methods of this paper, I find the best fit to *Gorgosaurus* is linear (Table S8). When co-linear points are used to fit a sigmoidal curve, it often creates parameter values which are very large (see for example, Table S3). This could potentially exacerbate differences in numerical precision and other implementation details of the regression algorithm. However this could not be a complete explanation because it is not the case for *Tyrannosaurus* or *Syntarsus*.

Nonlinear regression algorithms typically need to have “good” starting values to converge on the answer [102,103]. To account for this issue, the published fit parameters were used as the starting point for the attempted replicated fit. If the published fits were at a local minimum, then the replicated analysis should get stuck in the same place. Indeed, starting with the same fit ought to have made replication easier – rather than starting from an unknown start, we are starting with the answer and simply trying to confirm it. Despite this, the results could not be replicated.

The analysis was first done using only local minimization algorithms in the nonlinear regression model. However, I also tried global minimization algorithms. This covered the case where my algorithm got stuck on a local minimum, but not the case where the original paper had found a better, global minimum. Neither the use of the original fit parameters as the starting point, nor the use of multiple different global minimization methods allowed the results to be replicated.

I will review the details of the attempted replication on a paper by paper basis, in chronological order.

**Replication Issues with Regression in Erickson, Rogers and Yerby 2001 [20]**

Reference [20] states “A sigmoidal equation was used to model the growth of each species and least-squares regression analysis was used to fit the curves to the data”. Elsewhere in the same paper (the caption for Figure 2 in reference [20]), it states that “Note that the largest animals in the growth series are among the largest specimens known for each taxon and the growth asymptotes were set accordingly^14^.” Where the footnote 14 corresponds to reference [24] of this paper .

The interpretation of this statement is unclear to me. Since it might shed some light on my failure to replicate the results, I considered several alternative interpretations of the statement.

The first interpretation is that the statement is simply reinforcing the previous statement about use of least squares regression, and offers nothing new beyond that. Reference [24] uses least squares regression and its results can be replicated (at least approximately). By referencing that paper it would appear to reinforce the earlier statement.

A second interpretation of the statement is that maximum asymptotic size was simply set equal to the size of the largest specimen rather than being fit by regression. This interpretation would be a deviation from least squares regression as conventionally practiced, and thus might explain why I was unable to replicate them using that method.

Note that there are several reasons to doubt this interpretation.

First, this is not the methodology of reference [24], so the interpretation would be inconsistent with the footnote. The following quote from reference [24] makes this clear “The absence of a complete plateauing to the growth asymptote by the largest specimens used in this analysis may be attributable to these animals not being of maximal adult size”. This is also obvious from the plotted figure, and from the reported regression equation, which has a maximum asymptotic size of 25.2 kg, while the largest specimen has mass 19.87 kg. Reference [24] clearly did not set the maximum asymptotic value of the regression equation to the size of the largest individual.

Second, this would contradict the earlier statement in the paper. Third, we know that this was not done for *Psittacosaurus mongoliensis*, because it was not done in reference [24] and its results are at least approximately consistent with a best fit. This interpretation would suggest that differing methodologies were used for the taxa in Figure 2 of [20].

Fourth, and finally, judging from the plotted figures (Figure 2 of reference [20]), the maximum asymptotic size (i.e. the parameter $a$ ) has clearly not been set to the largest specimen in the 5 of the 6 taxa studied in the plots (Figure 2 of reference [20]). Only *Apatosaurus excelsus* has a plotted growth curve that passes through the largest point. All other taxa have a plotted growth curve that does *not* pass through the largest point. So, if we can take the plots to be an accurate representation of the fitting process used, they would appear to refute the interpretation that the statement means that the maximum asymptotic size was simply set to the largest value. Unfortunately, as will be discussed below, it is not clear how much one can entirely rely on the plotted figures, because there are serious issues with reproducibility of some of the plots.

A different interpretation of the “set accordingly” statement is that the value of parameter $a$ was not determined by regression, and instead was fixed “according” to some other method or procedure. Since the plotted figures show that it was not set to the largest value, we could test whether it was simply set to the published value in the paper. Given the ambiguity in the statement under consideration I proceeded to test it by performing regressions with function $\text{A}_{2}$.

If I could recover the published value of parameters $b$ and $c$ using regression on function $\text{A}_{2}$ then it would suggest that the interpretation of the statement was about fixing the value of parameter $a$ to the published value. However, this turned out not to be the case for either *Massospondylus* or *Syntarsus*. For *Psittacosaurus mongoliensis* it was unnecessary to test this because the result could be replicated (albeit not exactly) with function A.

One way to interpret this result is that the “set accordingly” statement is not simply related to setting the asymptotic value $a$ by itself. Setting $a$ does not allow us to replicate the values for $b$ and $c$. So whatever the “set accordingly” statement means it apparently must involve an analysis procedure that would impact more than just parameter $a$.

As a result of my failure to replicate the results, I cannot make any conclusions about the interpretation of the “set accordingly” statement. No interpretation that I tried seems to work.

My conclusion is that reference [20] clearly states that it uses least squares regression, but the published results for the three taxa that I could check can only be replicated (albeit only approximately) using that methodology for *Psittacosaurus mongoliensis*. The other results cannot be replicated using the stated methodology. This may, or may not, be related to some interpretation of the statement in the caption of Figure 2 of reference [20], but I was not able to find an interpretation of that statement which allowed replication, or even partial replication of the results.

In the case of *Syntarsus*, an attempt to replicate the plotted figures shows that the failure to replicate is likely related to the data set used. This will be discussed more below.

**Replication Issues with Regression in Erickson, Mackovicky, Currie, Norrell and Yerby 2004 [25]**

In the case of reference [25], in the methods section it states “All vertebrates show logistic (S-shaped) growth patterns during post-parturition/hatching development. Hence a logistic equation and least squares regression analysis were used to describe the relationship between the data.”

In this passage using the term logistic to describe S-shaped curves is not consistent with normal usage; the usual term for S-shaped curves is sigmoidal, of which logistic curves are but one of many types. As discussed in the main paper there is substantial literature devoted to growth curves that does not agree that all vertebrates follow sigmoidal curves, or even that they all follow asymptotic curves. However, the salient aspect of the statement for the purpose of determining the fitting methodology is that it very clearly states that least squares regression was used to fit logistic curves to the data points.

Unfortunately, of the four taxa in [25], the reported results can only be replicated for *Albertosaurus*. Even there, the fit obtained differs in some parameters, so it can only be approximately replicated.

Note also that both the original fit, and the replication best fit that I found, would be considered invalid by normal statistical criteria due to overfitting, because a four-parameter model is fit to four data points. If a two or three parameter model (i.e. functions $\text{A}_{2}$ and $\text{B}_{2}$) were used, it would still be considered overfit.

*Daspletosaurus* has only three data points for a four-parameter model, so I did not attempt to replicate it. *Gorgosaurus* and *Tyrannosaurus* cannot be replicated.

Note that my failure to replicate these results occurred for all of the functions tested, A, B, $\text{A}_{2}$ and $\text{B}_{2}$. Fixing the parameters $a$ and $d$ to the values in the published regression equations did not improve replication for parameters $b$ and $c$. As a result, it does not appear to be the case that fixing parameters $a$ and $d$, either separately or together, is an explanation of my failure to replicate the results.

**Replication Issues with Regression in Erickson, Mackovicky, *et al.* 2009 [18]**

Like the other papers, the regression result from [18] cannot be replicated. The published replication result is not a best fit, nor can one replicate the result using the variations on the functions attempted. In reference [18] the methods state “Sigmoidal growth equations were fit using least squares regression and used to describe these data.” This is clear and unambiguous but the method cannot be used to replicate the results. There are a number of irregularities with the plotted figure in this paper, as will be discussed more below.

**Reproducibility of Maximum Growth Rate Calculation**

Given a curve fit (equivalently, a regression equation) it is straightforward to calculate the peak growth rate. This is usually a simple formula based on the parameters of the model. As an example, for logistic function A shown above, the inflection point occurs at $t_{\text{inf}}=c$. The growth rate at the inflection point, which is the maximum growth rate, is then . This can be derived using elementary calculus.

Despite this simple relationship, I cannot reconcile the growth rates published in some of the papers with the regression equations published in the same study. As an example, for *Tyrannosaurus* reference [25] reports $a=5551$ and $b=0.57.$ This gives . Yet reference [25] quotes the growth rate as 767 kg/year. For other examples, see Table 3 in the main paper. While the difference in these values is not enormous, the fact that there is any difference at all is troubling given how simple the calculation should be.

I tried to reconcile this several ways. One idea is that the maximum rate was evaluated at specific ages. In the case of *Tyrannosaurus*, the inflection point is a non-integer age $t_{\text{inf}}=16.1$. This will be true in general — there is no reason to restrict the parameter c in logistic function A to integer values. I checked to see if the growth was evaluated at integer values, as is done for Table 5, which calculates the growth rate at data points (which are by definition integer ages) rather than at the inflection point. Unfortunately, this idea does not explain the discrepancy. For *Tyrannosaurus*, the growth rate of 767 kg/year occurs for $t=15.48$ and $t=17.72$years, using the parameters in the quoted regression equation.

Another attempt at replicating the results that I tried is that the maximum growth rate calculated in the paper was not really a rate, but was instead the amount of growth experienced in the year with the highest growth. This would be equivalent to averaging the growth rate over a year rather than evaluating it at a point in time.

Unfortunately this does not work either. According to the regression equation published in [25], *Tyrannosaurus* gains 763.6 kg in its 16^th^ year (i.e. mass at age $t=16$ minus mass at age $t=15)$, and 775.8 kg in its 17^th^ year. Neither of these numbers match the published figure of 767 kg/year. It also shows that the value of 767 kg/year is quite low — even averaging the growth rate over an entire year puts it (for 17^th^ year) at a larger value. So, if the published rate was calculated by averaging it would have to be over an interval of time longer than one year.

In most cases the published maximum growth rate is smaller than the figure calculated from the regression equation, but in the case of *Psittacosaurus mongoliensis*, in reference [20], the published maximum growth rate of 5.82 kg/year is higher than the rate calculated from the regression equation in the same paper of 5.5 kg/year. Of course, it is not possible for the average over a time period to be higher than the peak rate. This shows that time averaging could not be the source of the discrepancy, at least for this taxon.

Reference [24] quotes a maximum daily growth rate of 12.5 grams/day for *Psittacosaurus mongoliensis*, with a divisor for calculating annual growth of 374 days/year (see below for further discussion). This corresponds to a maximum annual growth rate of 4.675 kg/year. The regression equation for the same taxon in [24] gives a maximum growth rate of 4.4 kg/year.

This problem exists for every taxon in references [20,24,25], including taxa such as *Shuvuuia* or *Daspletosaurus* which have too few data. Reference [18] does not quote a numerical value for maximum growth rate (but appears to plot it in a figure), so there is no comparison to be made.

I have no explanation of the discrepancy between the published regression equations and the maximum growth rates. The regression equation and maximum growth rate appear in the same figure (Figure 2 of [20]), or in the figure and caption (Figure 2 of [25]).

**Reproducibility of Maximum Daily Growth Rate Calculation**

After calculating the maximum growth rates on an annual basis, several of the papers examined here then calculate the maximum growth rates on a daily basis, with units of grams/day. This is done in order to facilitate comparison to daily growth rates for extant animals.

In principle this should be a straightforward change of units: multiply by 1000 to convert kilograms to grams and then divide by the number of days in a year, which is 365.25 (within 2 decimal places of precision). That is because the growth recorded by LAG based age-size data occurs over the duration of the year, which in absolute time units has not significantly changed since the Mesozoic [45].

It is well known that the rotation rate of the earth has slowed due to tidal friction with the moon, and other effects. Within geologic timescales the number of rotations of the earth per orbital period, i.e. the number of solar days in a year has varied considerably. Evidence of the length of the day is recorded in various geological phenomena collectively known as rythmites, including growth lamina in fossil corals, mollusk shells, stromatolites or non-biological geological sedimentation phenomena [46-49,120]. Estimates from these methods range from a high count of 511 to 466 days per year 2.45 billion years ago [49] to 371 days per year 65 million years ago [120].

Reference [24] takes the following approach to calculating daily growth rates: “To allow comparison with the maximal growth rates for extant taxa, the rates were then converted to daily values by dividing each by 374 days, the length of an aptian/albian year (Wells, 1963)”. Similarly, reference [20] states, “Exponential stage growth rates were converted to daily growth rates using the appropriate number of day in the Mesozoic era”. Each quote references the classic paper of Wells on geochronometry using fossil corals [120]. References [18,25] do not explicitly discuss this methodology, but appear to follow it, as will be shown below.

If each Mesozoic solar day were the same value, in absolute time, as a present solar day (i.e. 86,440 seconds), and the larger number of days in the year represented a longer year, then this method of conversion would be correct.

Unfortunately, that is not the case. The time duration of a year (orbital period of the Earth) is constant. The reason that there are more solar days per Mesozoic year is because those days are proportionally shorter than solar days at present [46,47,49]. As a result, using different Mesozoic days/year divisors for each dinosaur taxa in effect converts each rate into different units. As a result the rates cannot be compared to each other unless they have the same divisor (same geologic age). They also should not be compared to extant animals. The fundamental issue is that “day” is not a fixed, scientific unit of time.

The length of a solar day and Earth’s rotation has environmental consequences captured in the rythmites. However, the LAG deposits found in dinosaur bones are intrinsically an annual phenomenon, which tell us the year-to-year growth. No histological argument in the literature has established that growth in dinosaurs is related to the Earth’s rotation.

As a matter of principle this renders all of the daily growth rate calculations in [18, 24,25] invalid for comparison with extant taxa, or with each other, if the rates come from taxa with different geologic ages. Such comparisons are the express purpose the daily growth rates are given in the papers.

Unfortunately this is not the only problem in the daily growth rates. Even setting aside the approach to calculating daily growth rates, there appear to be other internal inconsistencies. Given the published figures for annual growth rate and daily growth rate, simple arithmetic allows the recovery of the days-per-year divisor. The data of Wells [120] can be inverted with piecewise linear interpolation to recover a date from the days-per-year divisor. In order to check this with more recent research results, I also used the quadratic curve fit of Tenchov [121] which is based on data from Wells and five other more recent studies.

The maximum annual growth rate for *Shuvuuia deserti* is presented in [20] as 1.27 kg/year, while the daily growth rate is 3.4 grams/day. The correct daily conversion of the annual rate using a divisor of 365.25 is 3.48 grams/day. While that is very close, the numbers do not match within the numerical precision reported.

*Shuvuuia* has a divisor of 373.5 days/year, which via the Wells data corresponds to a geologic age of 94.9 mya; the Tenchov correlation places the geologic age at 111.5 mya. This is problematic because *Shuvuuia* is found only in the Campanian of Mongolia, and should have an age no more than 83.6 mya.

*Psittacosaurus mongoliensis* as presented in [20] has an annual growth rate of 5.82 kg/year, while the daily growth rate is presented as 12.5 grams/day. The correct daily conversion of the annual rate using a divisor of 365.25 is 15.9 grams/day or 27% higher than the published figure. The divisor used for conversion is 465.6 days/year which is way too high for the Mesozoic. The Wells data ends at 424 days/year, corresponding to 600 mya. The Tenchov correlation has a maximum of 448 days/year which occurs at 1.535 bya. Williams [49] presents rythmite data with 466 days at 2.45 bya. Clearly none of these ages would be appropriate for *Psittacosaurus*.

In [24] no annual growth rate is given for *Psittacosaurus mongoliensis*, but the divisor is given as 374 days/year. This corresponds to a geologic age under Wells of 100.5 mya, and under Tenchov of 116.0 days/year, which seems reasonable for the taxon. The daily growth rate is quoted as 12.5 grams/day, which is the same value as that presented in [20]. Using the published divisor we can calculate the effective maximum annual growth rate as 4.675 kg/year. The correct daily growth rate with a divisor of 365.25 is 12.8 grams/day.

*Syntarsus* has a claimed annual growth rate of 9.14 kg/year, while the daily growth rate is claimed to be 23.9 grams/day. The correct daily conversion of the annual rate using a divisor of 365.25 is 25.02 grams/day. The divisor used for conversion is 382.43 days/year which corresponds to an age of 187.8 mya via Wells and 198.5 mya via Tenchov. The Wells-based age appears to be correct for the taxon.

*Massospondylus* is presented as having an annual growth rate of 34.6 kg/year, while the corresponding daily growth rate is presented as 90.3 grams/day. The correct daily conversion of the annual rate using a divisor of 365.25 is 94.7 grams/day. The divisor used for conversion is 383.17 days/year which corresponds to an age of 192.0 mya via Wells and 206.0 mya via Tenchov. These ages seem appropriate for the taxon, which is known from the Early Jurassic [6].

*Maiasaura* is presented as having an annual growth rate of 1042 kg/year, while the corresponding daily growth rate is presented as 2793 grams/day. The correct daily conversion of the annual rate using a divisor of 365.25 is 2852 grams/day. The divisor used for conversion is 373.08 days/year which corresponds to an age of 89.5 mya via Wells and 107.2 mya via Tenchov. These ages are too old for the taxon, which is known from the Campanian [11].

Finally, the annual growth rate for *Apatosaurus* is 5466 kg/year and the daily growth rate is 14,460 grams/day. The correct daily conversion of the annual rate using a divisor of 365.25 is 14,965 grams/day. The divisor used for conversion is 378.01 days/year which corresponds to an age of 147.0 mya via Wells and 154.6 mya via Tenchov. These ages seem reasonable for the taxon.

In reference [25], the quoted annual growth rate for *Tyrannosaurus* is 767 kg/year, and the quoted daily growth rate is 2.07 kg/day. The correct conversion to daily growth rate from annual using a divisor of 365.25 is 2.10 kg/day. The implied divisor is 370.5 days/year, which corresponds to an age of 59.7 mya under Wells, and 83.3 mya under Tenchov. The Wells figure would make *Tyrannosaurus* younger than the KT boundary, which is inappropriate. The Tenchov age, on the other hand, is too old for *Tyrannosaurus* which is known from the Maastrichtian of North America.

Reference [25] does not list specific daily growth rates by name for the other taxa, but instead says that “The maximal growth rates for the three smaller tyrannosaurid taxa ranged from 0.31 to 0.48 kg d^-1^”. It seems safe to assume that the higher figure belongs to the tyrannosaurid in the paper other than *Tyrannosaurus* with the highest annual growth rate, which is *Gorgosaurus*. It has a quoted annual growth rate of 180 kg/year. The daily growth rate implies a divisor of 375 days/year which gives an age under Wells data of 112.3 mya; under Tenchov it is 1.5 mya. These ages are too old for a taxon from the Late Cretaceous.

The lower bound daily growth rate of 0.31 kg/day must therefore correspond to *Albertosaurus* which has a quoted annual growth rate of 114 kg/year. This gives a divisor of 28.1 mya under Wells and 57.7 mya under Tenchov, both of which are younger than the KT boundary and thus inappropriate for the taxon. No daily growth rate is quoted for the remaining taxon, *Daspletosaurus*.

Reference [18] calculates maximum daily growth rate for a small inset graph, but does not reveal the value in the paper so it is not possible to attempt replication.

In summary, the correct calculation of daily growth rate for the purpose of comparison with specimens from other geologic time periods differs from the numerical values presented in each of the papers. This is due to the use of the number of Mesozoic days in a year. In addition several of the taxa have divisors that appear to correspond to a geologic age that is much older or younger than is appropriate for the taxon.

**Reproducibility of the Plots**

Every growth paper includes figures that plot the growth data. This is part of a longstanding tradition; the visual indication of data point scatter and curve fit has always been used in science as a way to communicate results. The plots of the paper are a primary result, and ought to be just as reproducible as any result from the paper. In each of the cases studied here, the regression equations are found either in the figure itself, or in the figure caption, so it seems reasonable to expect that the plotted figure gives us direct insight into the analysis process.

I embarked on a study of the plots after discovering the replication problems. I plotted the published regression equations, and my attempted best-fit curves. The published regression equations in some cases visually appeared to be poor fits when I re-plotted them (see Figures S1, S2). Yet, the fits looked good in the figures plotted in the original papers. This discrepancy led me to look more closely at the plotted figures. Unfortunately, in several cases the plots in the figures cannot be replicated – the data set, curves, or both do not match what they purport to plot.

Attempts to replicate the plots from references [20,24,25] are shown in Figure S1 and S2. Apart from the two cases where the match is fairly close (*Albertosaurus* and *Psittacosaurus mongoliensis*), the various curves are visually poor fits to each other, and in some cases to the data points. In the original papers, the plotted curves were generally much better fits to the data sets.

Attempts to replicate the plots from reference [37] are shown in Figure S3, where the published regression equation curves are in red, while the attempts at replicating them are in black. These plots are very much what I would expect from the discussion in [37] which describes the results as low, medium and high bounds, which is visually apparent in Figure S3.

To explore the most problematic cases further, digital scans of the plots for *Psittacosaurus lujiatunensis* [18], *Massospondylus* [20], *Syntarsus* [20] and *Tyrannosaurus* [25] were edited to remove extraneous material. The plot for *Gorgosaurus* and *Albertosaurus* [25] was originally plotted on the same scale with *Tyrannosaurus*, and as a result their plots were too small to make this procedure effective. I did not attempt to analyze the plots for other taxa, although a similar analysis could be done for at least some of them.

The resulting images were then used as the backgrounds for plots set up to overlay plots of the regression equation on top of them. This allowed me to overlay the as-plotted data points recovered by image processing (see above) and the various curve fits made in attempting replication of the results. Each of these taxa is discussed below in its own section.

***Massospondylus* Plot Issues in [20]**

The original scan and overlaid variations are plotted in Figure S4. Panel A shows the original plot from [20], registered so data and functions can be plotted on top of it. Panel B shows the result of plotting data recovered by applying image processing to find the centers of the data points. The data points are then transformed from pixel coordinates to age-mass data pairs. These data pairs are then plotted using the registered overlay mechanism. This helps test the registration of the plot. It is not perfect but is more than adequate for the purpose.

Simple inspection of the data points shows that the points plotted in [20] are very different from the data set reported in the original source paper [6]. Reference [6], supplemented with additional data supplied by Chinsamy-Turan (Unpublished Data), comprises the source that the *Massospondylus* data set that I used, and which is also referenced by [20] as its source. This data set has 17 points, yet there are only 9 data points plotted in the *Massospondylus* plot of [20] (and thus in panels A and B of Figure S4). Clearly, the two data sets are not the same.

I used DME based on the largest specimen (age 15 years) with a mass of 245 kg to estimate masses for the data from [6] and Chinsamy-Turan. I will refer to the resulting age-mass data set as the Chinsamy-Turan *Massospondylus* data set. It is plotted in blue in panel C. Due to DME scaling, it matches original data for the plot from [20] at the point labeled a. Every data point where there is a unique size for a given age is found in both data sets.

However, there are also situations like that labeled c in panel C, where there are two or more data points in the Chinsamy-Turan *Massospondylus* data set that have the same age, but different sizes. In those cases the points plotted in [20] have a single size for that age, which is not the same as any of the points in the Chinsamy-Turan *Massospondylus* data set.

Although I cannot be certain, the result appears to be consistent with the *Massospondylus* data set having been filtered to identify cases where there is more than one size for a given age, and then to take the mean of the disparate sizes. That appears to be what is happening at the point labeled c. More generally the mean of the points with the same age appears to be a reasonable approximation for the data set plotted in [20] and panels A and B.

Filtering of the data prior to curve fitting is not normal in least squares regression. The mean of two data values is a linear function of the values. The least squares error function is a nonlinear function of the values and the curve. In general, least squares regression will give different curve fits for a data set and a mean-filtered data set. Pre-filtering using the mean will decrease the visual scatter on the plot, and increase the coefficient of determination, $R^{2}$.

The best fit of logistic function A to the full Chinsamy-Turan *Massospondylus* data set (scaled via DME) is plotted in blue, the best fit to function $\text{A}_{2}$ is in dashed magenta. The best fit for A and $\text{A}_{2}$ to the data set which is actually plotted (obtained via image processing – see panel B), is shown in green and dashed black. Within the span of the graph shown in C these look quite similar but the curves diverge strongly over large time scales. The blue curve (best fit to full Chinsamy-Turan *Massospondylus* data set) has a maximum asymptotic size parameter$a=4521733$, while the green curve (best fit to recovered data set as plotted) has$a=548$.

The red curve plotted in C is the regression equation reported in [20]. It is not a close match to either the original data, nor to the full Chinsamy-Turan *Massospondylus* data set (see the area labeled b on C). Its parameters are also very different, with$a=297$.

My conclusions are: the *Massospondylus* data set plotted in [20] is of unknown origin, but is consistent with mean filtering of points with the same age applied to the original data from [6]. The *Massospondylus* regression equation from [20] is also of unknown origin. It does not closely approach the data set, and it does not match the best fits to either the Chinsamy-Turan *Massospondylus* data set or the data points that it is plotted with. The plotted curve for *Massospondylus* in Figure 2 of [20] does not match a least squares regression fit to either the Chinsamy-Turan *Massospondylus* data set or the data points that it is plotted with, so it is also of unknown origin.

***Syntarsus* Plot Issues in [20]**

Figure S5 shows the plots for *Syntarsus*, both the original from [20] in A, the recovered data points B and the overlay with fits C, in a similar manner to *Massospondylus*.

The original source for [20], and the current work is by Chinsamy [5]. I supplemented the age data in [5] with femur length data from Chinsamy-Turan (Unpublished Data). I then scaled the mass estimates via DME. The maximum mass came from the highest mass specimen in the original plot (20.2 kg). I will refer to this as the Chinsamy-Turan *Syntarsus* data set. It is plotted in blue on C of Figure S5.

As with *Massospondylus*, it is immediately apparent that the data set plotted in [20] is quite different from the Chinsamy-Turan *Syntarsus* data set. Six data points are in the original plot (see panel A and B); there are 10 specimens in the Chinsamy-Turan *Syntarsus* data set. However, unlike *Massospondylus*, there does not seem to be any simple filtering relationship. The data set plotted in [20] has a specimen at age 7, labeled a, but the data set in [5] only has specimens up to age 6. Chinsamy-Turan *Syntarsus* data set has several cases of multiple points with the same age, this does not occur in the data set plotted in [20]. In a few cases, such as that labeled b in panel C, it seems possible that a mean was used to filter multiple data points for the same age. However, that is not true for points near the area labeled c in panel C. In that region, the points plotted in [20] (black diamonds) have no obvious relationship to the Chinsamy-Turan *Syntarsus* data points plotted in blue, near the region labeled c.

My conclusions are: the *Syntarsus* data set plotted in [20] is of unknown origin. The *Syntarsus* regression equation from [20] appears to be a best-fit to the plotted data set. It seems unrelated to the best fits for the Chinsamy-Turan *Syntarsus* data set. The curve plotted for *Syntarsus* in Figure 2 of reference [20] appears to be a best fit to the plotted data set. This strongly suggests that the modified data set, as plotted in the figure, was also the data set used in creating the regression equation.

It thus provides a potential explanation of why I was unable to replicate the regression result – I used the Chinsamy-Turan data set, while [20] appears to have used the data set plotted in the figure. So it would appear that I could have replicated the *Syntarsus* result had I been using the same data set.

Note that the issues that arise for *Syntarsus* are therefore quite different from those for *Massospondylus*. The relationship between the Chinsamy-Turan *Syntarsus* data set and the data set that is plotted do not appear to be a simple mean filtering of data points that have multiple sizes for a given age, indeed there seems to be no relationship between the two data sets at many points. In the case of *Syntarsus*, the published regression equation can be replicated for the recovered data set, but cannot be replicated for the Chinsamy-Turan data set. In contrast, for *Massospondylus*, I cannot replicate the regression equation using either the Chinsamy-Turan data set, or from the recovered data set.

This suggests that there may be multiple causes for the failure to replicate. In the case of *Syntarsus*, the problem appears to be the data set. Other taxa appear to have issues with the fitting procedure rather than the data set. *Massospondylus* appears to have problems with both the data set and the fitting procedure.

***Tyrannosaurus* Plot Issues in [25]**

Figure S6 shows the plot of *Tyrannosaurus* from [25]. Panel A shows a digital scan of the original plot data, with extraneous material removed. Panel B shows the original plot with the published data points plotted as an overlay.

In this case there is a strong correspondence between the published age-mass data and the data points in the plot. There is a puzzling discrepancy at the points labeled a, where the published points and the originally plotted points have slightly different ages. Since ages in this data set are integers, this is strange; the points in the original plot appear to have ages of about 1.7 and 22.28 years. While puzzling as to how it could have occurred, these small data point discrepancies are probably not significant compared to the other issues.

Panel C shows the age-mass data from [25] plotted in blue, along with the best fit using the logistic function A above in blue, the best fit to logistic function B in dashed green, and the regression equation from [25] in red.

The best fit (blue) curve is quite different than the regression equation (red curve). As an example, consider the area labeled b, where the blue curve substantially differs from the red curve. There is no difference, at least within the scope of this graph, between the fits of function A, and function B (blue curve, dashed green curve).

In addition the regression equation (red curve) is not the same as the curve plotted in [25] (black curve). This difference is most pronounced near the area labeled c. The difference is far larger than the correspondence found between features overlaid in other plots and appears not be related to any overlay registration issues.

This is particularly puzzling, and is not found in the cases of *Massospondylus* and *Syntarsus*. Apparently the curve plotted in Figure 2 of reference [25] does not match the regression equation, which appears in its own caption. I have no explanation as to how this could occur.

My conclusions are: the *Tyrannosaurus* data set plotted in Figure 2 of [25] does not match the published data perfectly, with two points that appear to have non-integer ages. The *Tyrannosaurus* regression equation from [25] cannot be replicated. It does not match the data well, nor does it match the best-fits to the data set. The curve plotted in Figure 2 of reference [25] also does not match the regression equation published in the caption of the original figure and is thus of unknown origin.

***Psittacosaurus lujiatunensis* Plot Issues in [18]**

Figure S7 shows the original plot, and several data set variations for *P. lujiatunensis*. Panel A is the original curve as plotted. Panel B shows the full data set (in red) from Table 1 of [18] (as discussed above), scaled via DME so that the specimen with the longest femur (the point labeled a in panel B) has a mass of 26.8 kg. As a result, the full data set and the one in the original plot match at that point.

The full data set and the data set in the original plot should match everywhere, but they do not. There are numerous points that appear in the full data set but not in the original plot – these appear as red points that are not associated with black points, as in the examples labeled b. Panel C tries to correct this by using the subset of points from Table 1 of [18] that were histologically aged.

This improves the correspondence but there are still issues. Several points, including the examples labeled c, are not correctly registered with their counterparts. In addition, there are points (labeled d) that do not have a corresponding point in the original plot, and points, such as that labeled e, that are in the original plot but are not in the histologically aged subset. In panel D, ad-hoc modifications were made to the data set to make it correspond better to the original plot. I do not know of any scientific justification for these modifications; they were made simply to match the original graph. Since the adjustments were both positive and negative in the y-direction (i.e. either smaller or larger mass) they could not be due to the DME scaling, or to overlay graph registration. The conclusion that I draw is that the points plotted in the original plot (Figure 6 of [18]) seem related to the data set published in the paper, but with some significant exceptions which are of unknown origin.

The regression equation from [18] (plotted in red) does not closely approach any of the data sets, nor does it resemble any of the best fits to functions D, E, F or G. It clearly has a different asymptotic value than the regression equation. It is of unknown origin.

In the case of *Tyrannosaurus*, the plotted curve differs from the published regression equation by a small, but significant amount. In the case of *P. lujiatunensis*, the plotted curve in the original figure bears little resemblance to the published regression equation.

The curve plotted in the original plot (thick dark blue line) is also mysterious. It does not match the regression equation, nor does it match any of the best fits to the various data sets. The curve that it is closest to (but still does not match) is the best fit regression for logistic function G, so it is possible that it comes from a fit to function G, or something similar.

The best fit lines also differ from each other. The values for the maximum asymptotic value $g\left(\infty\right)$ for the various fits to *P. lujiatunensis* data sets range from a low of 32.6 to a high of 88.4 in a set of closely related logistic functions. The reason for the disparity is that the data set is not actually a good fit for an asymptotic curve – the best fit found in the paper is to an increasing function. As a result, the values of $g\left(\infty\right)$ are mathematical artifacts, much like the examples in Figure 3 of the main paper, or Table S3.

Note that in addition to the function A, C, D, E, F and G, I also tried the variations with the versions of the functions $\text{A}_{2}, \text{C}_{2}, \text{D}_{2}, \text{E}_{2}, \text{F}_{2}, \text{and} \text{G}_{2}$, which have the value of the parameter $a$ fixed to equal the published regression equation. These functions are not able to replicate either the curve plotted in the figure, or the original regression equation. Thus, it would appear that fixing the parameter $a$, is not a way to replicate the results.

**Additional References for Text S1**

119. Qian M, Jiang Y, Jiang Y, Zhang Y, Chen R, et al. (2008) New evidence on fossil eggs of Cretaceous Tyrannosaurs in eastern China. Jiangsu Geol.

120. Wells JW (1963) Coral growth and geochronometry. Nature 197: 948–950.

121. Tenchov GG (2007) Rhytmites aid to reconstruct the evolution of the earth–moon system for the last 2.5 Ga. Bulg J Phys 34: 307–315.
