# Supplementary material for: Revisiting the Estimation of Dinosaur Growth Rates
Source: PLoS One. 2013 Dec 16;8(12):e81917. doi: 10.1371/journal.pone.0081917 (PMC3864909; doi:10.1371/journal.pone.0081917)
Supplement: Text S2 — Impact of the choice of independent variable. (DOCX) [file pone.0081917.s024.docx]

**Text S2. Impact of the Choice of Independent Variable**

In ordinary least squares (OLS) Model I regression, the independent variable is assumed to have zero error, whereas the dependent variable is assumed to have random errors. In the case of dinosaur growth curves, the variable that has zero (or very small) error is the bone size (for example, femur length or circumference of a LAG), whereas the age is estimated.

Previous work on dinosaur growth modeling uses age as the independent variable, thus violating the OLS assumption. In order to see what impact this has on estimates, I performed a Monte Carlo simulation experiment (see Supporting Figure S9). A logistic curve having maximum asymptotic growth *a* = 20 was chosen as the basis. A set of annually spaced samples from ages *t* = 2 to *t* = 20 was used to create a series of synthetic data sets for use in Monte Carlo simulations. Each heteroscedastic Monte Carlo experiment created 500 randomly perturbed sample sets by adding a normally distributed error of mean 0 and standard deviation $\sigma=\alpha t$, where *t* is the age and $\alpha$ is the proportional error in the age. Simulations were performed with $\alpha=0.10$. After the error was added, the resulting age was then rounded to the nearest integer value. A second family of simulations was made with homoscedastic errors. The same curve was sampled with normally distributed error with mean 0 and standard deviation $\sigma=1$year.

Throughout this study, I follow the statistical convention of referring to random variation as “error.” It is worth noting that the term covers all sources of variation in the age–size relationship. That includes variation that occurred within life that would cause a specimen to reach different sizes at the same age: environment, genetic variation, resources, climate and others. It also includes both variation due to the process of estimating age histologically, which could reflect differences in the remodeling of bone that obscures LAGs, and also variation due to the retrocalculation process (in the whole-bone method) or the alignment of specimens of different ages (in the longitudinal method).

As discussed in the main text, we lack good information on the probabilistic distribution of error in these dinosaur data sets. Further study of the histological process of age estimation is required to understand the likely distribution of errors, as well as even more basic error characteristics, such as whether the errors tend to be heteroscedastic or homoscedastic. We also must consider biological sources of error in the age–size relationship, such as highly plastic growth (which is typically dependent on random environmental factors).

As a result of these uncertainties, we cannot know whether these error models accurately match the error distribution in the dinosaur data sets. Nevertheless, these simulations illustrate in a general manner the impact of a small estimation error in age. As discussed in the paper, the few error estimates that exist are much larger, tend to be heteroscedastic and range from 10% to 15% ($\alpha=0.10, 0.15$).

As described elsewhere in this study, the sigmoidal curves were then fit to the 500 synthetic data sets. Both direct fit (using age as independent variable) and reverse fit (using age as dependent variable) were used to fit logistic curves. The results are shown in Fig. S9. Considerably greater variance is seen in the estimates made by using the direct fit. The ratio of standard deviations of the direct fit to the reverse fit is 2.08 for heteroscedastic errors and 4.09 for homoscedastic errors.

The clear implication of this analysis is that an incorrect choice of the variable for regression increases the standard error enormously. This finding is completely consistent with normal statistical practice, which respects the assumption that the independent variable is the error-free measure.
