# Supplementary material for: Revisiting the Estimation of Dinosaur Growth Rates
Source: PLoS One. 2013 Dec 16;8(12):e81917. doi: 10.1371/journal.pone.0081917 (PMC3864909; doi:10.1371/journal.pone.0081917)
Supplement: Text S3 — Impact of the choice of sigmoid curve. (DOCX) [file pone.0081917.s025.docx]

Text S3. Impact of the Choice of Sigmoid Curve

Supporting Figure S9 shows histograms and statistical measures for estimates of *a* in the Monte Carlo experiment described in Text S2, but using different analyzing curves than those originally used to sample the data. In this experiment, we know that $a=20$ in the original sampled curve. The point of the Monte Carlo simulation is to see how well we can estimate $a$ by using different curves.

The fact that the results have a wide range in the standard deviation for estimates (by a factor of three to seven) shows that the choice of curve is very important to the accuracy of the final result. Other curves (not plotted) have even higher standard deviations. The value of the means is also affected by the choice of curve. The use of attenuating curves to analyze a sigmoid creates an enormous range of values for both the mean and the standard deviation.

In this experiment, we know *a priori* that the samples were drawn from a logistic curve (which is sigmoid). In a real experiment, we do not know *a priori* which growth curve dinosaurs actually followed. The Monte Carlo experiment shows that our choice of model can have a pronounced effect on both the biological estimates and their accuracy. This finding reinforces the importance of testing multiple sigmoid curves and using an objective model selection criterion, such as the Akaike information criterion with correction (AIC_c_), to choose among them.
