# Supplementary material for: Revisiting the Estimation of Dinosaur Growth Rates
Source: PLoS One. 2013 Dec 16;8(12):e81917. doi: 10.1371/journal.pone.0081917 (PMC3864909; doi:10.1371/journal.pone.0081917)
Supplement: Text S4 — R 2 is not an adequate goodness-of-fit measure. (DOCX) [file pone.0081917.s026.docx]

Text S4. *R*^2^ Is Not an Adequate Goodness-of-Fit Measure

Virtually all prior studies of dinosaur growth quote the coefficient of determination, *R*^2^, for the fits that they produce. In most of these papers [18,20,24,25], that is the only goodness-of-fit metric reported. It is well known, however, that this measure is not adequate for that task, which is why AIC_c_ was developed [43].

Particularly when fitting growth curves, it is very easy to obtain high values of *R*^2^ for fits that are of low utility or even meaningless. Most growth curves are sufficiently flexible that they can be adjusted to fit well-ordered data with an impressive-looking *R*^2^ that approaches unity. The fits of asymptotic curves to linear or cubic data demonstrate this well (see Table S3). The high values of *R*^2^ make it appear that these asymptotic curves are excellent fits to both linear and cubic data sets, but that is misleading.

Of course, for many purposes the fits in Table S3 are acceptable. If our only goal is to interpolate values near existing data points, for example, then these fits perform that limited function reasonably well. The coefficient of determination, after all, measures the proximity of the fit curve to the data points.

However, the limitation of this measure is that it provides an inadequate basis on which to compare fits of various types. In particular, if you wish to test whether a data set has support for asymptotic behavior, then you must compare both increasing and asymptotic curves. The corrected Akaike information criterion (AIC_C_) is a metric better suited for this task than the coefficient of determination.
