# Supplementary material for: Revisiting the Estimation of Dinosaur Growth Rates
Source: PLoS One. 2013 Dec 16;8(12):e81917. doi: 10.1371/journal.pone.0081917 (PMC3864909; doi:10.1371/journal.pone.0081917)
Supplement: Text S5 — Impact of finite sample size. (DOCX) [file pone.0081917.s027.docx]

Text S5. Impact of Finite Sample Size

In practice, measured data points are rarely spaced regularly across the range of age and size. For many dinosaur taxa, specimens of very young juveniles are rare, possibly due to taphonomic factors. Very old individuals are intrinsically rare in any wild population because they are the survivors of considerable mortality earlier in life [32,101]. Moreover, dinosaur fossils are rare, and those that are available for histological analysis of age are rarer still, so comparatively few data points exist for any taxa (see Table S6).

To study the effects of a finite sample, samples were drawn from the logistic curve shown in Fig. S8 by using a normal distribution in age, having a mean of 11.5 and a standard deviation of 2.875 years. These values were selected so that most samples fall in the central part of the sigmoid curve, as typically occurs with observed data. Each Monte Carlo experiment was based on an array of 500 data sets, each containing the same number of points, $N,$sampled from the curve. Experiments were conducted with $N=5$to *N* = 20 data points per sample. All sampled ages were rounded to the nearest integer value.

A control set was sampled at random intervals from the curve with no error. Additional experiments were run in which the age was affected by normally distributed heteroscedastic error having a mean of 0 and a standard deviation of $\sigma=\alpha t$, where *t* represents the age and $\alpha$ is the proportional error rate, chosen as $\alpha=0.05, 0.10, 0.15$ across the experiments. A third set of experiments was run in which homoscedastic errors having a mean of 0 and a standard deviation chosen from $\sigma=0.25, 0.5, 1$ years were added to the age.

Throughout this study, I follow the statistical convention of referring to random variation as “error.” It is worth noting that the term covers all sources of variation in the age–size relationship. That includes variation that occurred within life that would cause a specimen to reach different sizes at the same age: environment, genetic variation, resources, climate and others. It also includes both variation due to the process of estimating age histologically, which could be due to differences in the remodeling of bone that obscures LAGs, and also variation due to the retrocalculation process (in the whole-bone method) or the alignment of specimens of different ages (in the longitudinal method).

A set of 15 asymptotic curves was chosen to fit the sampled data. Five of the choices have two parameters (Extreme Value 2, Logistic 2a, Michaelis Menton 2, Rational 2z, von Bertalanffy 2), five have three parameters (Extreme Value 3a, Logistic 3, Michaelis Menton 3, Rational 3a, von Bertalanffy 3) and five have four parameters (Logistic 4a, Richards 4d, Sloboda 4, Rational 4, von Bertalanffy 4).

A set of 12 increasing curves was also chosen, including five curves having two parameters (Cubic 2b, Quadratic 2b, Linear 2, Cubic 2, Power 2), five having three parameters (Quadratic 3, Power 3, Power 3b, Exponential 3 and Persistence 3a), and two having four parameters (Persistence 4a, Persistence 4b). After fitting, those curves yielding the best fits ($\text{ΔAIC}_{c}=0$) were identified and tallied.

When the control set contained five samples, an increasing curve was the best fit 48.6% of the time (Fig. S10). It may seem surprising that samples drawn without error from a logistic curve could be best fit by increasing curves. But this result makes sense for at least two reasons. First, random selection of the age of the samples ensures that many of the points to be fit fall within the central, nearly linear portion of the curve. Increasing curves tend to provide the best fit unless multiple points in the same sample set fall within all three regions of an asymptotic curve. Second, limiting the number of samples to five guarantees that $\text{ΔAIC}_{c}=\infty$ for any curves having three or more parameters. Because a five-point sample can only be fit usefully by two-parameter curves, which may not be flexible enough to match the logistic curve, an increasing curve often produces the best result. (This result also shows the value of testing multiple functions. If the experiment had excluded two-parameter asymptotic curves, then none of the five-point samples would have been fit by an asymptotic curve.)

A control set containing six samples was best fit by increasing curves only 5.8% of the time. With seven samples, this fraction dropped to 2.8%, and with 20 samples it fell further to only 0.4%. Increasing the number of samples drawn from the logistic curve clearly increases the likelihood that the best fit will be asymptotic. This result strongly demonstrates the point, discussed in the main text, that a small number of samples can make an asymptotic curve appear linear. Here I find that this is quite likely, even with zero error, if the data set contains only five samples.

Adding heteroscedastic error to the ages in the samples increases the likelihood that an increasing curve is a best fit. With five-point samples and $\alpha= 0.10$, the fraction of increasing fits jumps to 52.2%, and it does not drop with increasing sample size in the same way that the zero-error case does. With seven-point samples, increasing curves are found to provide best fits 41% of the time; with 10-point samples, 27.8 %; and with 20-point samples, 12.4%.

Something similar occurs with homoscedastic errors. When experimental sets were constructed having a constant error and a standard deviation of 0.5 years at each age, five-point samples were best fit by increasing functions 47.4% of the time, seven-point samples 36.6% of the time, 10-point samples 24.6% of the time, and 20-point samples 9.2% of the time.

Fig. S10 plots the change in the fraction of increasing fits against sample size and error rate. The results from these simulations show that increasing curves can, in some cases, provide the strongest fits for finite samples of an asymptotic curve. The probability of this outcome is affected both by the sample size and by the amount and kind of noise in the data set.
