# Supplementary material for: Revisiting the Estimation of Dinosaur Growth Rates
Source: PLoS One. 2013 Dec 16;8(12):e81917. doi: 10.1371/journal.pone.0081917 (PMC3864909; doi:10.1371/journal.pone.0081917)
Supplement: Text S6 — Hutchinson et al. 2011 and DME. (DOCX) [file pone.0081917.s028.docx]

Text S6. Hutchinson *et al.* 2011 and DME

Hutchinson *et al.* [41] used an innovative new approach to estimating mass of dinosaurs by digitally reconstructing a 3-D model of the animal and then calculating its volume and mass. They stated that one of their goals was “to apply these techniques to *Tyrannosaurus* to revisit estimates of its growth rate and the reliability of DME.”

Such as reevaluation is possible because their method offers an independent way to estimate the mass of specimens in an ontogenetic sequence. Hutchinson *et al.* present three models for *T. rex* masses: minimal (Min), maximal (Max) and the average of the minimal and maximal model masses (Ave). To apply DME to these scenarios, one uses the mass $m_{\text{max}}$ of the specimen having the longest femur length $l_{\text{max}}$,. Then the DME estimates for the masses of the other specimen are calculated from their femur lengths by

$m_{\text{DME}}\left(l\right)= m_{\text{max}}\left(\frac{l}{l_{\text{max}}}\right)^{3}= m_{\text{Sue}}\left(\frac{l}{l_{\text{Sue}}}\right)^{3}$.

In the case of the estimates of Hutchinson *et al.*, the largest specimen is FMNH PR 2081 (“Sue”), $m_{\text{Sue}}$ is the mass of the Sue specimen in the scenario, and $l_{\text{Sue}}$ is the Sue femur length of 1.312 meters. Due to this scaling relationship, the DME estimate for Sue in each scenario will always match the Hutchinson estimate.

Table S9 presents the results. The Min, Max and Ave scenarios are from Hutchinson *et al.* [41]. The Hybrid scenario uses the Ave values for all specimens except Sue, which has the Min mass. This scenario is not an official scenario in Hutchinson *et al.*, but it is used in that paper in constructing their growth curve. It may be relevant if the very large mass of Sue is considered to be excessive or due in part to taphonomic distortion of the specimen.

The results show that DME scaling gives very different estimates than the method of Hutchinson *et al.* The smallest specimen (BMR P2002.4.1, “Jane”) has a DME estimate that is between 316% and 322% of the estimate of Hutchinson *et al.*, depending on the scenario. The other specimens have a DME estimate that is typically 140% to 160% of the Hutchinson *et al.* estimate, although in one case it is as low as 114%. In the Hybrid scenario, the results are a bit better, but the Jane mass is still too high by more than a factor of two.

The reason is shown graphically in Fig. S11. We can fit two different models to the Hutchinson *et al.* mass estimates in each scenario. The first is a DME-like function that has an arbitrary scaling power $p$:

$m_{\text{Fit}1}\left(l\right)= m_{\text{Sue}}\left(\frac{l}{l_{\text{Sue}}}\right)^{p}$.

The second is more general, two-parameter power law:

$m_{\text{Fit}2}\left(l\right)= \alpha l^{p}$ .

The results are shown in Fig. S11, in which the function $m_{\text{Fit}1}\left(l\right)$ is plotted in black and $m_{\text{Fit}2}\left(l\right)$ is in red. Because there are only five data points (two of which are nearly on top of one another), these fits should be interpreted as the scaling between the minimum and maximum specimens (Jane to Sue), rather than as a true scaling law. With only five data points, one cannot say with certainty whether this power law would hold up at the smallest sizes. In Erickson *et al.* [25], a *T. rex* specimen with a femur length of 25.2 cm is included; the Jane femur is over three times as long at 78.8 cm. It would be very instructive if the Hutchinson *et al.* method could be applied to a very small specimen.

Nevertheless, $m_{\text{Fit}1}\left(l\right)$ delivers remarkably consistent scaling exponents, $p=5.34$ to $p=5.38$, across the three main scenarios. This consistency suggests that the scaling exponent may fundamentally be determined by the rules for 3-D flesh construction. The Hybrid scenario yields a lower value of $p=4.5$.

DME scales mass as femur length cubed. This large difference in exponent explains why DME does not match the Hutchinson *et al.* results; its scaling with femur size is fundamentally different. As a result, DME scaling applied to the Hutchinson *et al.* data set generates results that are inconsistent with Hutchinson’s estimates for the four specimens they study. This does not mean that DME is wrong, of course; it is unknown whether the true mass of *T. rex* matches the Hutchinson *et al.* results, which are significantly higher than previous estimates of *T. rex* mass. But clearly the DME and Hutchinson *et al.* results cannot both be correct.

The Hutchinson paper did not present results on DME in this manner. Instead, they followed an indirect procedure that created two age–mass curves. The first curve was constructed by using selected versions of their masses, and age estimates obtained from various source studies for these specimens. The second age–mass curve was made by using the ages and masses obtained from DME scaling. They then fit a four-parameter logistic model to each and compared the curves, asking whether the DME results fit within the 95% confidence band of their results.

That basic procedure is complicated by several problematic details. Of the five specimens examined by Hutchinson *et al.*, age estimates were only available for four: Jane, which was aged by LAG counting by Hutchinson *et al.*; MOR 555 from [80]; and Sue from [41]. With only four specimens, fitting a four-parameter model is an extreme case of overfitting. Under normal statistical practice, such a fit would not be accorded any validity.

In any event, a clerical error in the calculation of the growth curve was responsible for the DME curve lying inside the 95% confidence band (Makovicky, Unpublished Data). This error led Hutchinson *et al.* to report that DME provided a “robust first approximation” for estimating body mass. Correction of the clerical error makes it clear that the DME curve lies outside the 95% confidence band and changes their conclusion. This correction brings the Hutchinson *et al.* results in line with the finding here that DME is not consistent with the Hutchinson ontogenetic scaling.
