# Supplementary material for: Revisiting the Estimation of Dinosaur Growth Rates
Source: PLoS One. 2013 Dec 16;8(12):e81917. doi: 10.1371/journal.pone.0081917 (PMC3864909; doi:10.1371/journal.pone.0081917)
Supplement: Text S7 — Discussion of growth models and fitting. (DOCX) [file pone.0081917.s029.docx]

Text S7. Discussion of Growth Models and Fitting

Mathematical models have long been used in biology and other sciences to model phenomena that grow. The collection of 62 asymptotic functions used in this study (see Table S1) is not intended to be fully comprehensive but provides reasonable coverage of the range of models that are commonly in use.

Some widely used growth models are defined by differential equations that do not admit closed-form solutions. These require a much more computationally intensive approach to curve fitting and are not included here. Many of the functions are mathematically equivalent to one another under nonlinear transformations and changes of variable. Indeed, one of the reasons that there are so many functions listed is that many of them are simply different mathematical parameterizations of a much smaller set of underlying functions.

Differences in the parameterization are important in the context of curve fitting because the numerical properties of the nonlinear optimization algorithms used in nonlinear least squares regression are often dependent on the parameterization [103,122,123]. As a result, it is often the case that one parameterization will work better with a given data set than another parameterization of the same mathematical function. As an extreme example, the Richards function is a general form of many of the other functions—including logistic, negative exponential, von Bertalanffy, Chapman Richards and (in a limiting case) Gompertz. In principle, one could just fit the Richards function and ignore the others. In practice, however, that approach does not work very well because the Richards function can be numerically ill-behaved and could fail to fit data that could easily be fit by a logistic or von Bertalanffy function.

A second reason that a highly generalized model like Richards may not be superior is that model selection criteria, such as the corrected Akaike information criterion (AIC_c_) used in this study, compare models based in part on the number of parameters in the model. The Richards model has four parameters, whereas its more specific, subset forms (such as logistic) have just three parameters; the extra free parameter of Richards effectively interpolates among the various subset models. Because of its extra parameter, the Richards model sometimes receives an inferior value of ΔAIC_c_ when compared to one of its own subset models.

Another issue is that the literature may present the identical function and parameterization under several different names. The names used here are widely used, but many have synonyms or aliases.

An additional complication is that growth functions are typically specified for fitting with time as the independent variable. As discussed in the main paper text, and in supporting text, this study used size (typically, bone dimension) as the independent variable. Instead of fitting with the function directly, the fits were thus performed with the inverse function. Although simple in principle, using inverse functions introduces some numerical difficulties because they frequently involve functions, such as square root or log, which require positive arguments. As a result, the curve-fitting procedures sometimes yielded results that are complex numbers.

Numerical precisions issues are important because the best fit in some cases has large parameters—up to $10^{55}$ for one of the fits to a line found in Table S3. Because the functions often use exponentials or high powers, it is surprisingly easy to generate numerical underflow or overflow, even when using double precision (64-bit) arithmetic.

Curve fitting was performed by using the function NonlinearModelFit in Mathematica 9.01. Details of numerical algorithms were found to affect the results. Version 9.0.1 of the software gave improved answers for some of the cases in the study when compared to Mathematica 8.0, due to improvements in the algorithms or implementation of NonlinearModelFit.

The starting point from which the nonlinear regression begins is very important for the success of the procedure. I used functions from the literature to guess appropriate starting values. In difficult cases, multiple starting guesses were compared. Multiple attempts were made using NonlinearModelFit with methods that performed both local and global optimization. The corrected Akaike information criterion (AIC_c_) was used to rank the results of different guesses and algorithms to find the best fit [96].

Some of the global optimization methods, such as simulated annealing, are stochastic in nature, and therefore the results can vary from one run to the next. In practice, the result of numerical nonlinear regression is not definitive—one cannot, in general, prove that the result that is found is the absolute best possible fit—so results here should be interpreted simply as the best fits found in the attempts made in the study. All numerical nonlinear regression studies share this property [102,103].

Additional References for Text S7

122. Ratkowsky DA (1990) Handbook of Nonlinear Regression Models. New York, N.Y.: Marcel Dekker.

123. Tjørve KMC, Tjørve E (2010) Shapes and functions of bird-growth models: how to characterise chick postnatal growth. Zoology 113: 326–333.
